# Phased high-quality genome of the gymnosperm Himalayan Yew assists in paclitaxel pathway exploration

**DOI:** 10.1093/gigascience/giaf026

**Published:** 2025-04-04

**Authors:** Zhenzhu Li, Hang Zong, Xiaonan Liu, Xiao Wang, Shimeng Liu, Xi Jiao, Xianqing Chen, Hao Wu, Zhuoya Liu, Zhongkai Wang, Yongqiang Wang, Yi Liu, Botong Zhou, Zihe Li, Qiuhui Du, Jing Li, Jian Cheng, Jie Bai, Xiaoxi Zhu, Yue Yang, Guichun Liu, Li Zhang, Huifeng Jiang, Wen Wang

**Affiliations:** New Cornerstone Science Laboratory, Shaanxi Key Laboratory of Qinling Ecological Intelligent Monitoring and Protection, School of Ecology and Environment, Northwestern Polytechnical University, Xi’an, Shaanxi 710072, China; New Cornerstone Science Laboratory, Shaanxi Key Laboratory of Qinling Ecological Intelligent Monitoring and Protection, School of Ecology and Environment, Northwestern Polytechnical University, Xi’an, Shaanxi 710072, China; Key Laboratory of Systems Microbial Biotechnology, Tianjin Institute of Industrial Biotechnology, Chinese Academy of Sciences, Tianjin 300308, China; Cooperative Innovation Center of Industrial Fermentation (Ministry of Education & Hubei Province), Key Laboratory of Fermentation Engineering (Ministry of Education), Hubei Key Laboratory of Industrial Microbiology, National “111” Center for Cellular Regulation and Molecular Pharmaceutics, Hubei University of Technology, Wuhan 430068, China; Key Laboratory of Systems Microbial Biotechnology, Tianjin Institute of Industrial Biotechnology, Chinese Academy of Sciences, Tianjin 300308, China; Jiaxing Synbiolab Biotechnology Co., Ltd., Jiaxing 314006, China; Jiaxing Synbiolab Biotechnology Co., Ltd., Jiaxing 314006, China; Jiaxing Synbiolab Biotechnology Co., Ltd., Jiaxing 314006, China; Jiaxing Synbiolab Biotechnology Co., Ltd., Jiaxing 314006, China; Jiaxing Synbiolab Biotechnology Co., Ltd., Jiaxing 314006, China; New Cornerstone Science Laboratory, Shaanxi Key Laboratory of Qinling Ecological Intelligent Monitoring and Protection, School of Ecology and Environment, Northwestern Polytechnical University, Xi’an, Shaanxi 710072, China; New Cornerstone Science Laboratory, Shaanxi Key Laboratory of Qinling Ecological Intelligent Monitoring and Protection, School of Ecology and Environment, Northwestern Polytechnical University, Xi’an, Shaanxi 710072, China; Sanjie Institute of Forage, Yangling, 712100, China; New Cornerstone Science Laboratory, Shaanxi Key Laboratory of Qinling Ecological Intelligent Monitoring and Protection, School of Ecology and Environment, Northwestern Polytechnical University, Xi’an, Shaanxi 710072, China; New Cornerstone Science Laboratory, Shaanxi Key Laboratory of Qinling Ecological Intelligent Monitoring and Protection, School of Ecology and Environment, Northwestern Polytechnical University, Xi’an, Shaanxi 710072, China; New Cornerstone Science Laboratory, Shaanxi Key Laboratory of Qinling Ecological Intelligent Monitoring and Protection, School of Ecology and Environment, Northwestern Polytechnical University, Xi’an, Shaanxi 710072, China; Jiaxing Synbiolab Biotechnology Co., Ltd., Jiaxing 314006, China; Key Laboratory of Systems Microbial Biotechnology, Tianjin Institute of Industrial Biotechnology, Chinese Academy of Sciences, Tianjin 300308, China; Key Laboratory of Systems Microbial Biotechnology, Tianjin Institute of Industrial Biotechnology, Chinese Academy of Sciences, Tianjin 300308, China; Key Laboratory of Systems Microbial Biotechnology, Tianjin Institute of Industrial Biotechnology, Chinese Academy of Sciences, Tianjin 300308, China; Key Laboratory of Systems Microbial Biotechnology, Tianjin Institute of Industrial Biotechnology, Chinese Academy of Sciences, Tianjin 300308, China; College of Traditional Chinese Medicine of Jinggangshan University, Ji’an 343009, China; State Key Laboratory of Genetic Resources and Evolution, Kunming Institute of Zoology, Chinese Academy of Sciences, Kunming 650201, China; Chinese Institute for Brain Research (CIBR), Beijing 102206, China; Key Laboratory of Systems Microbial Biotechnology, Tianjin Institute of Industrial Biotechnology, Chinese Academy of Sciences, Tianjin 300308, China; New Cornerstone Science Laboratory, Shaanxi Key Laboratory of Qinling Ecological Intelligent Monitoring and Protection, School of Ecology and Environment, Northwestern Polytechnical University, Xi’an, Shaanxi 710072, China

**Keywords:** gymnosperm, *Taxus wallichiana*, phased high-quality genome, paclitaxel, 2-oxoglutarate/Fe(II)-dependent dioxygenase (ODD)

## Abstract

**Background:**

*Taxus wallichiana* is an important species for paclitaxel production. Previous genome versions for *Taxus* spp. have been limited by extensive gaps, hindering the complete annotation and mining of paclitaxel (known as Taxol commercially) synthesis pathway-related genes.

**Results:**

Here, we present the first phased high-quality reference genome of *T. wallichiana*, which significantly improves assembly quality and corrects large-scale assembly errors present in previous versions. The 2 haplotypes are 9.87 Gb and 9.98 Gb in length, respectively, and all 24 chromosomes were assembled with telomeres at both ends. Based on this high-quality genome (TWv1), we inferred that the candidate sex chromosome of *T. wallichiana* is chr12, and its sex determination system may follow a ZW model. Particularly, we identified and experimentally validated a batch of 2-oxoglutarate/Fe(II)-dependent dioxygenases (ODDs), which may be key C4β–C20 epoxidases in the paclitaxel synthesis pathway.

**Conclusions:**

This study not only provides a valuable data resource for gene mining in the biosynthetic pathways of secondary metabolites, such as paclitaxel, but also offers the highest-quality reference genome of gymnosperms to date for the identification of sex chromosomes, facilitating comparative genomic studies among gymnosperms.

## Introduction

The Himalayan yew (*Taxus wallichiana*, NCBI:txid147273) is a gymnosperm species endemic to the regions east of the Himalayas in China. It has long been utilized as a medicinal plant due to its higher paclitaxel (known as Taxol commercially) content compared to other yew species [1]. Gymnosperm genomes are notoriously large and complex, characterized by numerous gene families, repetitive sequences, transposons, genome rearrangements, and abundant unique noncoding sequences, all contributing to genome expansion and complexity [2, 3]. Previously, 3 haploid genomes of the genus *Taxus* have been published [4–6], including *Taxus wallichiana* (*T. wallichiana*) from our group [4]. However, these genomes were assembled using either second-generation sequencing or third-generation sequencing technologies with relatively high error rates, resulting in a large number of unfilled gaps (11,130, 8,004, and 12,092 gaps, respectively) and many assembly errors. These assembly issues and unphased assembly pose challenges for the identification of key metabolite biosynthetic enzymes and sex chromosomes.

Gymnosperms typically exhibit dioecy, with sex determination in dioecious taxa relying on heteromorphic chromosomes in male and female plants [7]. However, in most gymnosperms, sex differentiation is at an early stage of formation and is driven by epigenetic control, such as differences in cytosine methylation between the sexes [8]. Dioecy has independently arisen multiple times during the evolution of gymnosperms [9], leading to the development of various sex determination systems. Currently, it is still difficult to uncover and explain the exact mechanisms behind the origin of these systems. For instance, the sex chromosomes of the 3 previously published *Taxus* genomes have yet to be identified. Obtaining a high-quality genome of the Himalayan yew will facilitate more in-depth studies on the sex differentiation of gymnosperms.

Paclitaxel is a renowned drug for treating breast cancer, ovarian cancer, and lung cancer [10–13]. However, the content of paclitaxel in yew is extremely low, even in the Himalayan yew (about 0.001%) [14]. In addition, the long growth cycle and scarce resources of yew trees have severely limited the availability of paclitaxel on a large scale from the natural sources. On the other hand, the synthesis pathway of paclitaxel is assumed to be very complex [15], and comprehensively, excavation of synthesis enzymes has been hindered by incomplete yew genome assemblies. Therefore, to obtain a high-quality yew genome and elucidate the paclitaxel synthesis pathway in yew has been a critical step in identifying enzymes for the paclitaxel synthesis through synthetic biology.

For a long period, only 14 enzymatic reactions in the paclitaxel biosynthetic pathway were resolved [16]. Notably, a new epoxidase, 2-oxoglutarate/Fe(II)-dependent dioxygenase (ODD), has been discovered [17]. It is hypothesized to catalyze the first step of taxadiene oxidation, specifically responsible for C4β–C20 epoxidation. However, due to the inability to isolate any epoxidase products in large quantities for nuclear magnetic resonance structural identification, the catalytic process of this epoxidase remains speculative [17]. In plants, ODDs are nonheme iron proteins that are soluble and localized in the cytoplasm. ODD enzymes are involved in various biological processes, including the biosynthesis of specialized metabolites such as plant hormones and flavonoids [18]. Oxygenation/hydroxylation reactions catalyzed by dioxygenases are particularly important in paclitaxel biosynthesis research. Recently, Zhao et al. [19] discovered that the single enzyme CYP725A4 with C5 hydroxylation function can catalyze 2 consecutive epoxidation events, leading to the formation of an oxetane ring. Jiang et al. [20] identified a bifunctional cytochrome P450 enzyme TOT1, which can directly convert the olefin part into an epoxide and an oxetane ring, respectively, but this enzyme cannot function as an isomerase to convert the epoxide ring into the oxetane ring. These pieces of evidence suggest that ODDs and CYP450s play key roles in the upstream biosynthesis steps of paclitaxel and indicate the presence of different epoxidases in yews, which may imply the existence of multiple catalytic synthesis pathways for paclitaxel precursors, hence exhibiting substrate promiscuity.

In this study, we obtained the first phased high-quality genome of a gymnosperm species, the Himalayan yew, and assembled the longest telomere-to-telomere (T2T) chromosome to date. This is not only the highest-quality genome reported for yew so far but also the highest-quality genome assembled across the entire gymnosperm phylum. Through comprehensive annotation and analysis of this genome, we inferred candidate sex chromosomes in the Himalayan yew and identified a set of crucial ODD enzymes, which exhibit C4β–C20 epoxidase activity as validated by our intensive experiments. The high-quality assembly and in-depth analysis of the *T. wallichiana* genome presented in this study provide valuable resources for identifying key enzymes involved in paclitaxel biosynthesis and offer important references for understanding the complex genomes of gymnosperms.

## Materials and Methods

### Plant materials

To investigate the genome of *T. wallichiana*, fresh leaves were collected from a female Himalayan yew tree, estimated to be at least 50 years old, cultivated at the Kunming Institute of Botany, Chinese Academy of Sciences. For RNA sequencing (RNA-seq), fresh leaves and fruits were collected from the same tree.

#### Sequencing

Sample collection was based on precise handling protocols aimed at extracting high-molecular-weight genomic DNA from Himalayan yew tissues. Initially, DNA extraction was performed using the CTAB method, followed by purification with the QIAGEN Genomic kit (catalog number 13343; QIAGEN) to ensure the DNA was suitable for conventional sequencing analysis. Library preparation followed PacBio’s standard protocol for HiFi target libraries, using a 15-kb preparation scheme. Sequencing was performed on a PacBio Sequel II instrument using Sequencing Primer V2 and the Sequel II Binding Kit 2.1 at Haorui Genomics. For Nanopore sequencing, genomic DNA (gDNA) samples were extracted from Himalayan yew young leaves using the QIAGEN Genomic DNA extraction kit (catalog number 13323; QIAGEN). DNA purity was measured with a NanoDrop One UV-Vis spectrophotometer (Thermo Fisher Scientific), with OD260/280 ratios between 1.8 and 2.0 and OD260/230 ratios between 2.0 and 2.2. DNA libraries were subsequently loaded into the preassembled flow cells of the Nanopore PromethION sequencer (RRID:SCR_017987; Oxford Nanopore Technologies) for sequencing.

#### Genome assembly and quality assessment

Using the hifiasm software (Hifiasm 0.24.0-r702, RRID:SCR_021069) [21, 22], we integrated ONT UL sequencing data, HiFi sequencing data, and Hi-C sequencing data to perform the phased assembly of the *T. wallichiana* genome (parameter: hifiasm -l 0). Using BWA software (BWA 0.7.17-r1198-dirty, RRID:SCR_010910) [23, 24], we aligned the Hi-C data to the genome, which is merged by 2 haplotype assemblies. The HapHiC pipeline [25, 26] was then employed to scaffold the contigs to the chromosome level, followed by manual correction of any assembly errors. The completeness of the genome was assessed using BUSCO (BUSCO 5.3.2, RRID:SCR_015008) [27, 28]. Merqury software (Merqury 1.3, RRID:SCR_022964) [29, 30] was used to evaluate the genome’s completeness and the error rate of each chromosome and the overall error rate using HiFi reads. HiFi reads and ONT UL reads were aligned to the reference genome using minimap2 software (Minimap2 2.26-r1175, RRID:SCR_018550) [31, 32] to assess depth distribution and to evaluate haplotype depth distribution based on HiFi reads.

#### Genome annotation

We used EDTA (EDTA 2.0.1, RRID:SCR_022063) [33, 34] to construct a high-quality, nonredundant repeat sequence library. Gene prediction was performed by BRAKER3 (BRAKER3 3.0.8, RRID:SCR_018964) [35–40] using the soft-masked genome. Annotation was performed using protein sequences from 20 gymnosperms and about 300 Gb of transcriptome data (Supplementary Table S1). Transcriptome sequence alignment and assembly were performed using HISAT (HISAT 2.2.1, RRID:SCR_015530) [41–43]. The annotation results were evaluated using BUSCO (BUSCO 5.3.2, RRID:SCR_015008) [27, 28] with the Gymnosperm_odb10 and embryophyta_odb10 datasets. All protein-coding genes were retrieved and functionally annotated by blast searches against databases, including UniProtKB/Swiss-Prot, UniProtKB/TrEMBL, NR, and KEGG. They were also subjected to GO annotation and protein family annotation by InterProScan (InterProScan 5.45–80.0, RRID:SCR_005829) [44, 45].

#### Mining ODD enzymes

ODD gene family proteins were identified across the entire genome using tblastn [46]. We downloaded a publicly available dataset containing 40 transcriptome samples, including different cell lines with high and low paclitaxel yields, as well as 5 tissue types. Transcriptome data alignment and quantification were performed using HISAT (HISAT 2.2.1, RRID:SCR_015530) [43, 47] and StringTie (StringTie 2.1.7, RRID:SCR_016323) [48, 49]. Next, we calculated the expression correlation matrix between ODD and paclitaxel synthesis-related genes. Clustering was applied to this matrix, and ODD genes within the cluster containing the highest number of known paclitaxel synthesis genes were selected as candidate genes for subsequent experimental validation.

#### ODD enzyme activity assay

The ODD genes to be validated were codon-optimized for *Saccharomyces cerevisiae* and then cloned into the yeast expression vector Ycplac22 using the Gibson Assembly method. This constructed expression vector was subsequently transformed into our preengineered *S. cerevisiae* chassis strain [4] for cytoplasmic taxa-4(5),11(12)-diene(taxadiene) production, where it was expressed and functionally analyzed. Using the Gibson Assembly technique, we assembled the YCPlac22 vector, a bidirectional terminator, a bidirectional strong promoter, and the candidate ODD gene sequences together. The recombinant plasmids were sequenced for validation and then transformed into host cells producing taxadiene.

To detect the synthesis of taxadiene and the activity of ODD enzymes, the *S. cerevisiae* strain was first cultured in 3 mL of defective medium in test tubes for 48 hours at 30°C and 800 rpm. The seed culture was then inoculated into 40 mL of fresh medium at a ratio of 1:50. After 10 hours of cultivation, 5 mL of n-dodecane and 2 mL of 40% glucose were added to initiate 2-phase fermentation, promoting product separation and accumulation. This cultivation process continued for 4 days at 30°C and 220 rpm. Subsequently, the upper organic phase was collected by centrifugation at 3,600 rpm for 10 minutes for gas chromatography–mass spectrometry (GC-MS) analysis.

For GC-MS analysis, samples were analyzed using a Thermo Scientific TRACE 1600 coupled with a TSQ 9000 triple quadrupole mass spectrometer. A 2-µL sample was injected into a TG-1MS GC column (30 m × 0.25 mm × 0.25 µm). The initial column temperature was set to 80°C and held for 1 minute, followed by an increase to 200°C over 7 minutes. The injection port and transfer line temperatures were set to 300°C and 290°C, respectively, to ensure efficient sample injection and transfer.

#### Mechanistic analysis of ODD enzymes

Structure preparation: All ODD enzyme structures in this study were modeled using OpenFold (OpenFold 2.0) [50, 51] with default parameters. The top-ranked structure based on predicted Local Distance Difference Test (pLDDT) scores was selected for further analysis. Substrate molecule structures were obtained from PubChem (CID: 167825), and the coenzyme alpha-ketoglutarate structure was also sourced from PubChem (CID: 51).

Molecular docking: All molecular docking procedures were performed using the Watvina method (Watvina, RRID:SCR_026282) [52]. AutoDock Tools (AutoDock Tools 1.5.6, RRID:SCR_012746) [53–55] was used to prepare the substrate molecules and protein receptors. The docking box was defined as a cubic box with a side length of 40 nm. The docking energy range was set to 5 kcal/mol, with an exhaustiveness parameter of 12 and a maximum of 100 output conformations. Reasonable conformations were identified based on the distance between the substrate reactive site and the iron-oxo (FeO) center being within 5 Å and a negative docking score. From these reasonable conformations, the most appropriate one was selected for further structural analysis based on manual assessment.

Molecular dynamics simulation: Molecular dynamics simulations were conducted using Gromacs (Gromacs 2023.2, RRID:SCR_014565) [56–58]. The AMBERff14SB force field parameters were applied. Small molecules were converted into GROMACS-compatible ITP-format topology files using the AmberTools toolkit (AmberTools 24, RRID:SCR_018497) [59, 60], and the GAFF force field was used to parameterize ligand atoms [61], with parameters generated by the Antechamber tool (Amber 20, RRID:SCR_014230) [59, 62]. The TIP3P model was used for water molecules. The protein–ligand complex was placed in a cubic periodic water box with a minimum boundary distance of 10 Å. Sodium (Na^+^) and chloride (Cl^−^) ions were added to neutralize the system. Long-range electrostatic interactions were handled using the Ewald method. The system’s energy was minimized using the steepest descent method for a maximum of 5,000 steps. Subsequently, the system was equilibrated with 100 ps of NVT simulation followed by 100 ps of NPT simulation. The production dynamics simulation was then run at 300 K and 1 bar pressure with periodic boundary conditions, for a duration of 100 ns with a time step of 2 fs. Energy, trajectory, and structural data were collected every 1 ps. Root Mean Square Deviation (RMSD), RMSF, and other analyses were performed using the built-in trajectory analysis modules of Gromacs (Gromacs 2023.2, RRID:SCR_014565) [56–58].

## Results

### Phased high-quality genome of *T. wallichiana* (Twv1)

The PacBio Revio platform and the Oxford Nanopore Technologies (ONT) ultra-long (UL) sequencing technology were employed to conduct further sequencing of the same individual of the Himalayan yew as previously reported by Cheng et al. [4]. This effort yielded a total of approximately 722 Gb (72.2×) of HiFi reads, approximately 875 Gb (87.5×) of ONT UL reads, and 1,077.1 Gb (∼100×) Hi-C reads. Among these, the N50 length of HiFi reads exceeded 16 kb, while the N50 length of ONT reads approached 54 kb (Supplementary Tables S2, S3, S4). Through the integration and assembly of these data using the hifiasm software (Hifiasm 0.19.8-r602, RRID:SCR_021069) [21, 22], we obtained a phased assembly with 2 haptypes.

**Figure 1:**
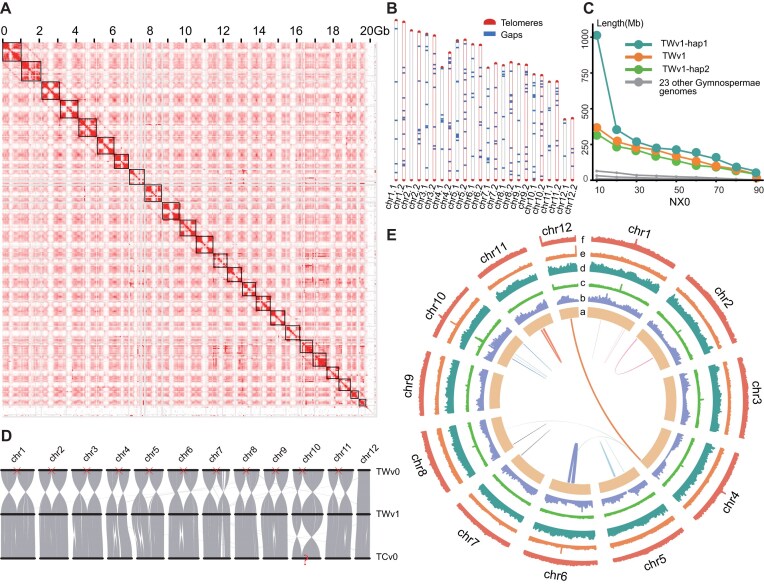
Overview of the Himalayan yew genome TWv1 assembly. (A) Hi-C chromatin interaction heatmap of the TWv1 assembly. Each black box represents a single chromosome. Chromosome numbers correspond to those in panel D. (B) Distribution of gaps and telomere sequences across the 12 chromosomes in the TWv1-hap1 genome. Chromosome 2 is assembled to telomere-to-telomere (T2T) completeness. (C) Comparison of NX0 (N10–N90) between the Himalayan yew and 23 other gymnosperm genomes. (D) Collinearity comparison of different versions of the yew reference genomes. Red crosses indicate 11 intrachromosomal assembly errors present in TWv0, while red question marks denote a potential “inversion” assembly error in the TCv0 chromosome. (E) TWv1 Circos graph. From the innermost to the outermost circle (a–f) are (a) chromosome length, (b) gene number distribution, (c) GC content, (d) LTR/Gypsy distribution, (e) LTR/Copia distribution, and (f) DNA transposon distribution. The lines in the center of the circle indicate collinear regions.

Utilizing the Hi-C data, we anchored the contigs of both haplotypes onto 24 chromosomes, achieving an anchoring rate of 97.5%. The continuity of the Hi-C heatmap indicates that the genome has no large-scale assembly errors and that the 2 haplotypes were correctly phased (Fig. 1A, Table 1). As a result, we obtained a high-quality haplotype-resolved genome, TWv1, with a total genome size of approximately 20.3 Gb, consisting of 24 chromosomes and a contig N50 length of 169.4 Mb (Table 1). Each chromosome contains complete telomeres at both ends, and the TWv1 genome includes only 201 gaps (Fig. 1B, Supplementary Table S5). The genome sizes of haplotype 1 (TWv1-hap1) and haplotype 2 (TWv1-hap2) are 9.87 Gb and 9.98 Gb, respectively.

**Table 1:** Assembly statistics of *Taxus* genus *TWv0, TWv1, TCv0*, and *TYv0* genomes

Assembly	TWv0	TWv1	TWv1-hap1	TWv1-hap2	TCv0	TYv0
QV (consensus quality value)	19.3585	59.5658	60.1364	60.7947	\	\
Completeness	52.39%	99.36%	\	\	\	\
Contig N50 (Mb)	8.6	169.4	213.7	136.6	2.44	2.89
Genome size (bp)	11,119,083,473	20,348,291,365	9,868,113,160	9,979,816,178	10,232,176,133	10,737,203,084
gap numbers	8,004	201	85	116	12,092	11,130

Multiple approaches were employed to assess the completeness and continuity of the TWv1 genome. A completeness assessment using the *k*-mer–based method performed by Merqury software (Merqury 1.3, RRID:SCR_022964) [29, 30] revealed a genome quality value (QV) of approximately 59.6, with QV values for individual chromosomes ranging from 57.22 to 64.06 (Table 1; Supplementary Table S6). The overall completeness is 99.36%, which corresponds to approximately 1 base error per 1 Mbp (Table 1). For the BUSCO assessment of genome completeness, both haplotypes achieved over 95% completeness in the Gymnosperm_odb10 dataset, while in the Embryophyta_odb10 dataset, both haplotypes reached approximately 90%. This difference may be attributed to the lower suitability of the Embryophyta_odb10 dataset for gymnosperms (Supplementary Table S7) [63]. The Hi-C heatmap also demonstrated the continuity of the TWv1 genome assembly, further confirming the high precision of the assembly quality (Fig. 1A). The TWv1 genome assembly corrected 12 large-scale assembly errors present in earlier versions, including 11 intrachromosomal assembly errors in *T. wallichiana* (as TWv0 in this study), where the 2 arms of the chromosomes exhibited “inversion” assembly errors (Fig. 1D) [4]. Additionally, a potential “inversion” assembly error was identified in the chromosome of *Taxus chinensis var. mairei* (TCv0) [5], with coordinates consistent with those in TWv0. However, due to the different species and the inability to identify telomeric sequences in TCv0, this could also be attributed to interspecies differences [5]. In conclusion, we have successfully completed the first phasing of the Himalayan yew genome and addressed its haplotype assembly issues. We have also significantly reduced the number of gaps in the Himalayan yew genome. Compared to previous versions (TWv0), the new high-quality haplotype genome TWv1-hap1 has only 85 remaining gaps, and TWv1-hap2 has only 116 gaps, which is a substantial improvement over the TCv0 and *Taxus yunnanensis* (TYv0) genomes [64], which recorded 12,092 and 11,130 gaps, respectively [5, 64]. Comparative analysis of contig N50 lengths showed that the assembly quality of TWv1 far surpasses that of 23 other gymnosperm genomes (Fig. 1C; Supplementary Table S1), with contig N50 lengths 19.7 times, 69.4 times, and 58.6 times greater than those of TWv0, TCv0, and TYv0, respectively (Table 1). Importantly, the genome of Twv1 has identified all 24 telomeres (Supplementary Table S8). Since chr2 of haplotype 1 has no gaps and the telomeres are complete, its assembly has reached the T2T level. This chromosome, with a length of 1.01 Gb, is the longest T2T chromosome reported to date.

We also obtained high-precision annotation for the TWv1 genome. Using EDTA software (EDTA 2.0.1, RRID:SCR_022063) [33, 34], we annotated the composition and precise locations of repetitive sequences in Himalayan yew. In the TWv1 genome, the repetitive sequences of TWv1-hap1 and TWv1-hap2 account for 8.3 and 8.4 Gb, respectively, making up 84% of the total genome. Among these, long terminal repeat (LTR) elements are the most abundant type of repetitive sequence, comprising 69% of all repetitive sequences in both haplotypes and representing 58% of the total genome in TWv1-hap1 and TWv1-hap2. DNA transposons account for more than 19% of both haplotype genomes (Supplementary Tables S9, S10). Additionally, we predicted 37,766 and 38,579 protein-coding genes in the TWv1-hap1 and TWv1-hap2 genomes, respectively. The average coding sequence length is 1,119 bp and 1,111 bp, with each gene containing an average of 4.44 and 4.38 exons and average exon lengths of 252.27 bp and 253.34 bp, respectively (Supplementary Tables S9, S10). We performed a detailed analysis of the GC content, gene numbers, LTR-Gypsy/Copia distribution, and transposable element (TE) distribution in the TWv1-hap1 genome using 500-kb windows on each of the 12 chromosomes. This information was comprehensively plotted into a circos diagram of the TWv1-hap1 genome (Fig. 1E).

### Genetic polymorphism analysis and sex differentiation in Himalayan yew

**Figure 2:**
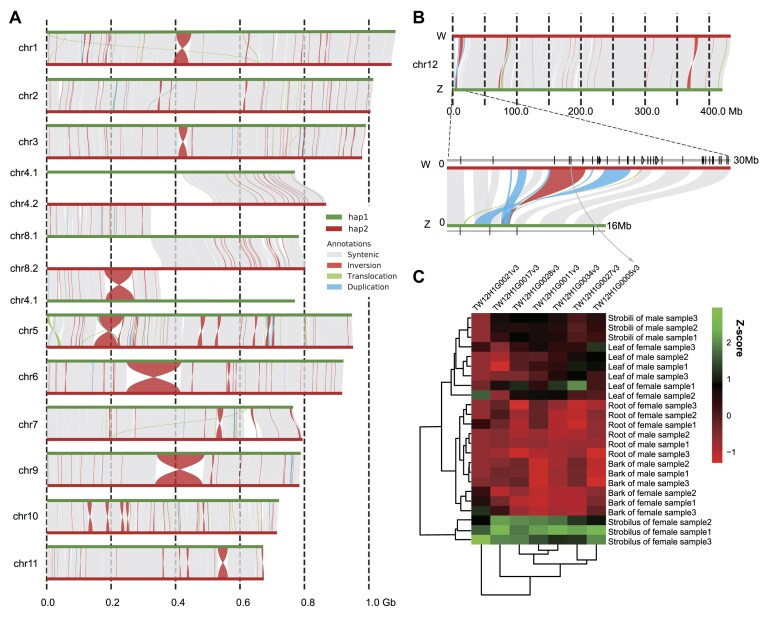
Structural variations in TWv1. (A) Structural variations between the 2 haplotypes of chr1–chr11. Gray lines represent collinear regions, red lines represent insertions, green lines represent translocations, and blue lines represent duplications. (B) Structural variations and nonhomologous regions between the Z/W haplotypes of the sex chromosome chr12. (C) Heatmap of gene expression differences in the nonhomologous regions of the Z/W chromosomes.

To investigate the genetic polymorphism of Himalayan yew, we identified structural variations (length >50 bp) between the 2 haplotype genomes in TWv1 (Fig. 2). The analysis revealed that the total length of all structural variations accounts for 13.4% of the entire genome, including 64 duplications, 66 translocations, and 267 inversions (Supplementary Tables S11, S12). We found 4 ultra-large structural variations greater than 100 Mb in length between the 2 haplotypes, including a 366-Mb interchromosomal translocation between chr4 and chr8, as well as inversions of 169 Mb and 148 Mb on chr6 and chr9, respectively (Fig. 2A, Supplementary Fig. S1, Supplementary Table S13). These events may pose certain obstacles to homologous chromosome recombination during meiosis in this individual. Additionally, chromosome length is a characteristic of variation between haplotypes. To avoid misjudgment of chromosome length due to assembly errors, we evaluated the collapse regions of the genome based on the mapping depth of HiFi reads, using a 100-Kb window (Supplementary Table S14). Statistics indicate that a total of 218 Mb of regions in the genome exhibit collapse, and they only distributed in chr4, chr7, chr8, with a collapse rate of 1.07% (Supplementary Fig. S2). The total length of collapsed sequences in TWv1-hap1 is 42 Mb, with a collapse rate of only 0.42%. By summing the collapsed lengths and assembled lengths of each chromosome, we predicted the actual lengths of Himalayan yew chromosomes. Comparisons of lengths between all pairs of homologous chromosomes show that the length differences between haplotypes range from 0.12% to 1.71%. Aside from the chromosomal translocations observed in chr4 and chr8, the largest length difference is found in chr12, with a sequence length difference of 7.2 Mb, accounting for 1.71% and 1.68% of the chr12 of 2 haplotypes, respectively. This length difference is caused by the 0- to 28-Mb region of chr12.2 and the 0- to 16-Mb region of chr12.1 (Fig. 2B, Supplementary Table S14). According to previous karyotype studies [65], this pair of chromosomes also exhibits length differences and was considered the candidate sex chromosomes. Given that the individual assembled in this study is female, as indicated by its yield of seeds (Supplementary Fig. S3), this result suggests that the sex determination in Himalayan yew might follow the ZW model, with chr12.1 and chr12.2 representing the candidate Z and W chromosomes, respectively (Fig. 2B). The identification of a Dof zinc finger protein family gene, which has been found being involved in flowering control [66, 67], in the highly variable regions between the 2 homologous chromosomes further suggests they may be the sex chromosomes. However, more direct evidence is needed to validate this hypothesis.

To further investigate sex differentiation in Himalayan yew, we compared the Z and W chromosomes and identified structural variations between homologous chromosomes. We found that the 0- to 30-Mb region of the W chromosome and the 0- to 16-Mb region of the Z chromosome contain 10.0 Mb (33.3%) and 4.0 Mb (25.0%) of unaligned regions, respectively. In these nonhomologous regions, the Z and W chromosomes have undergone 5 duplications of approximately 4.0 Mb and inversions of 3.7 Mb and 1.0 Mb, respectively (Fig. 2B, Supplementary Tables S11, S12). Using existing transcriptome data of roots, leaves, bark, male flowers, and female flowers from the *Taxus* spp. in the database [5], we identified 7 genes in these regions that are expressed and exhibit sex-specific expression in the flower (Fig. 2C). Interestingly, the TW12H1G0005v3 gene on the W chromosome is highly expressed only in the flower of female individuals but is lowly expressed or not expressed in male individuals (Fig. 2C). The TW12H1G0005v3 gene has been identified as belonging to the Dof zinc finger protein family—CDF2 (cycling Dof factor 2). CDF proteins are a unique class of transcription factors in the plant DOF family. Studies have shown that CDF transcription factors play a crucial role in photoperiod response for flowering control in *Arabidopsis* [66, 67]. These results suggest that TW12H1G0005v3 may be related to sex differentiation.

### Mining ODD enzymes in Himalayan yew

**Figure 3:**
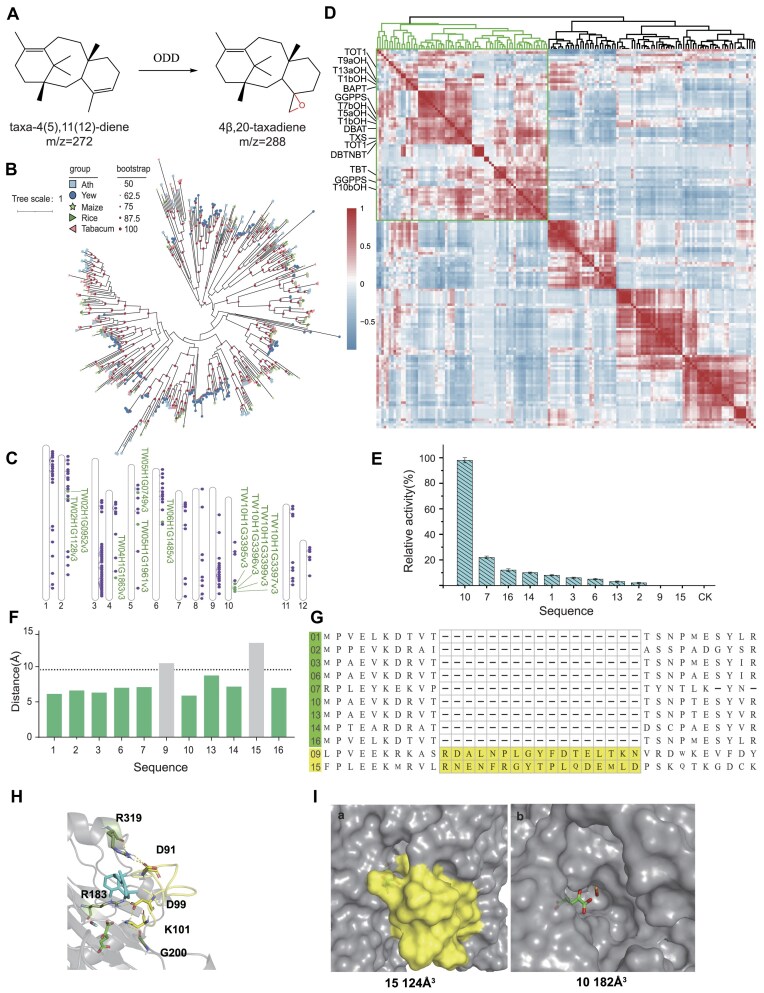
Discovery and activity analysis of ODD enzymes. (A) Predicted epoxidation function of ODD. (B) ODD gene family tree (showing the distribution and evolutionary relationships of the identified genes). (C) Chromosomal distribution of paclitaxel synthesis-related ODD enzymes. Purple circles indicate all ODDs in TWv1, and green circles indicate candidate genes screened by coexpression analysis. (D) Heatmap of coexpression of genes related to the paclitaxel synthesis pathway. (E) Relative activity assay of the epoxidation function of the 11 candidate genes. (F) Comparison of catalytic distance and activity of the 11 candidate genes. Green indicates catalytic activity; gray indicates no catalytic activity. (G) Multiple sequence alignment analysis of the 11 candidate genes. (H) Analysis of hydrogen bond formation in loop 86–101 of the catalytic pocket of sequence 15. (I) (a) Catalytic pocket of sequence 15, and (b) catalytic pocket of sequence 10.

ODDs play a critical role in the biosynthesis of a wide range of specialized metabolites in plants. Based on amino acid sequence similarity, ODDs are typically classified into 3 main subfamilies: DOXA, DOXB, and DOXC. The DOXA class serves as the prototype of ODDs and is involved in the alkylation and oxidative demethylation of nucleic acids and histones. The DOXB class is conserved across all plant taxa and is involved in the proline 4-hydroxylation in cell wall protein synthesis. The DOXC class ODD enzymes are of particular interest due to their role in the specialized metabolism of various plant chemicals, including phytohormones and flavonoids. Most ODDs in terrestrial plants belong to the DOXC class [18]. In conjunction with previous studies and the powerful and unique functions of ODD enzymes [17], we hypothesize that ODD enzymes may catalyze the epoxidation of taxadiene, the initial taxane substrate in the paclitaxel biosynthesis pathway (Fig. 3A). To this end, we conducted a comprehensive classification and mining of the ODD family genes in the Himalayan yew genome. We identified the DOXC members in the Himalayan yew ODDs and constructed a gene family tree along with Arabidopsis, maize, rice, and tobacco (Fig. 3B). The Himalayan yew has 158 genes belonging to the ODD DOXC family, with many genes formed by tandem repeat expansions (Fig. 3C, Supplementary Table S15). The expansion of these genes in the Himalayan yew suggests their potential role in specific phenotypes, such as paclitaxel synthesis.

To further identify candidate genes and validate our hypothesis, we utilized multitissue transcriptome data from public databases. This dataset includes multiple cell lines with high and low Taxol production, as well as 5 types of tissues (bark, root, leaf, strobilus, and strobili) from both male and female trees, totaling 40 samples. We calculated the gene expression similarity matrix for ODD DOXC family genes and known paclitaxel biosynthesis-related genes across these samples (Fig. 3D). Notably, the DOXC family genes and paclitaxel synthesis-related genes formed a coexpression module comprising 16 known synthesis genes and 70 ODD family genes. We hypothesize that these genes possess potential taxadiene epoxidase activity. Using multiple sequence alignment and phylogenetic tree analysis, along with consideration of different evolutionary clades and the chromosomal distribution of these genes, we selected 11 genes as candidates for experimental validation (Fig. 3C, Supplementary Table S16).

### Activity validation and analysis of ODD enzymes

To investigate the functional roles of the selected ODD genes in the paclitaxel biosynthesis pathway, we introduced 11 candidate genes into the yeast cytosolic taxadiene production chassis we previously constructed [4]. GC-MS activity validation results showed that 9 out of the 11 genes exhibited potential C4β–C20 epoxidation activity (Fig. 3E, Supplementary Fig. S4), while sequence 9 and sequence 15 did not display any catalytic activity.

To analyze the epoxidation activity of the ODD enzymes, we performed molecular dynamics simulations on the 11 experimentally validated enzymes. Since our previously constructed yeast cytoplasmic taxadiene production chassis primarily produces endotaxadiene (taxa–4 (5), 11 (12)—diene), we further studied the catalytic distance of endotaxadiene (from C11 to Fe in FeO) within the catalytic cavity. The results indicated that enzymes with catalytic activity generally had shorter catalytic distances, while those without catalytic activity showed longer catalytic distances (Fig. 3F). Subsequently, we performed multiple sequence alignment of the 11 enzymes and analyzed their 3-dimensional structures. We found that the catalytic cavities of sequences 9 and 15, which lacked epoxygenase activity, had an additional 17 amino acids (in yellow) compared to the other sequences with catalytic activity (Fig. 3G). This extra sequence might be the reason for the different catalytic activities.

By comparing the catalytic cavities of sequences 10 and 15, we discovered that the insertion of these 17 amino acids in sequence 15 appeared as loop 84–101 protruding into the catalytic cavity in the 3-dimensional structure (Fig. 3H). This loop formed hydrogen bonds with other residues in the catalytic cavity, specifically D91-R319 (63.2%), D99-R183 (96.5%), and K101-G200 (86.3%). These hydrogen bonds stabilized the position of the loop, and the loop occupied space within the catalytic cavity, making it difficult for the substrate to bind with the coenzyme and FeO within the cavity. Additionally, the presence of this loop likely hindered the entry and exit of the substrate from the catalytic cavity. In summary, we hypothesize that the lack of epoxygenase activity in sequences 9 and 15 is likely due to the presence of this loop, which obstructs further interaction between the substrate and ODD. We also measured the catalytic pocket sizes of sequences 10 and 15 (Fig. 3I), finding that the catalytic pocket of sequence 15, which lacked catalytic activity, was only 124Å³ due to the loop insertion, whereas the catalytic pocket of sequence 10, which did not have the loop, was 182Å³, further supporting our hypothesis.

### Collinearity and P450 analysis of TWv1

**Figure 4:**
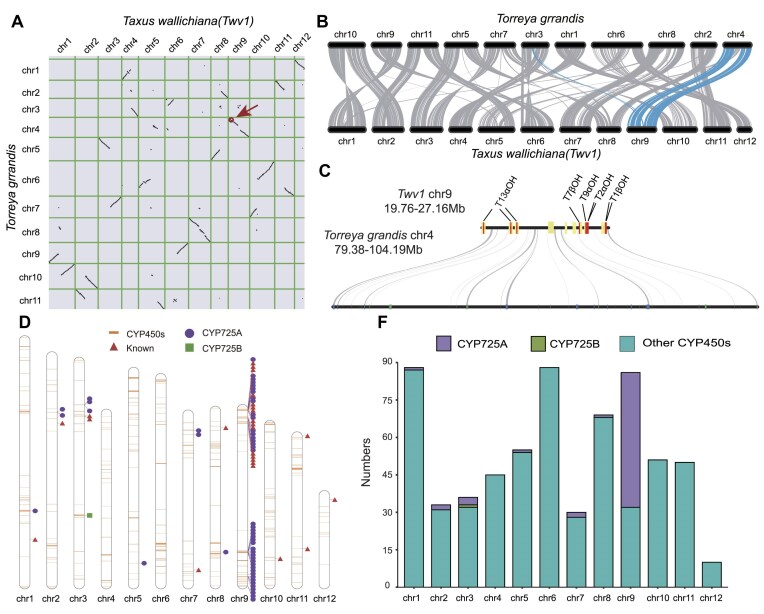
Collinearity and P450 cluster analysis of TWv1. (A) Chromosomal collinearity between TWv1 and *T. grandis*. Collinear regions between TWv1 and *T. grandis* are indicated by black dots. The red arrows indicate the locations of the paclitaxel biosynthesis gene clusters. (B) Collinear graph between TWv1 and *T. grandis*. Gray lines represent collinear regions between TWv1 and *T. grandis*, and blue lines represent collinear regions between chr9 of TWv1 and *T. grandis*. (C) Collinear region of the gene cluster on chr9 of TWv1 and chr4 of *T. grandis*. Yellow lines on chr9 of TWv1 indicate enzymes belonging to the CYP725A subfamily, and red lines indicate enzymes involved in paclitaxel biosynthesis. (D) Distribution of P450 gene clusters across the 12 chromosomes. Orange lines indicate all P450 enzymes in TWv1, red triangles indicate characterized enzymes, purple circles indicate enzymes belonging to the CYP725A subfamily, and green squares indicate enzymes belonging to the CYP725B subfamily. (E) Histogram of P450 numbers across the 12 chromosomes. Purple bars represent enzymes belonging to the CYP725A subfamily, green bars represent enzymes belonging to the CYP725B subfamily, and blue bars represent all other CYP450 enzymes outside the CYP725 family.

*Taxus* spp. is the only large-scale source of the anticancer drug paclitaxel [1]. *Torreya grandis* (*T. grandis*), a gymnosperm of the Cephalotaxaceae family, is closely related to *Taxus*. Recent research shows that *T. grandis* separated from *T. wallichiana* around 68.5 million years ago [68], and *T. grandis* produces little to no paclitaxel [69]. Comparative studies among closely related species are one method to explore the evolutionary history of genes. To investigate the evolutionary trajectory of the paclitaxel biosynthesis pathway, we examined the collinearity relationship between the positions of CYP450 genes in *T. wallichiana* (TWv1) and *T. grandis* (Fig. 4A, 4B). In *T. wallichiana*, the paclitaxel biosynthesis gene cluster is concentrated in the 19.76- to 27.16-Mb region of chr9. This region shows good collinearity with the 79.38- to 104.19-Mb region of chr4 in *T. grandis*. However, there are no direct homologs of the CYP450 genes within the paclitaxel biosynthesis gene cluster in *T. grandis* (Fig. 4C). This suggests that the emergence of the paclitaxel biosynthesis gene cluster occurred after the divergence of these 2 species, which is after 68.5 million years ago.

The expansion of the CYP725A subfamily may have played a crucial role in the evolution of paclitaxel biosynthesis. Utilizing high-quality genomic data, our research group performed a comprehensive analysis of the classification of all CYP450 genes in *T. wallichiana* by comparing them to the CYP450 database using standard sequence similarity cutoffs [5] (Fig. 4D) and precisely identified their copy numbers (Fig. 4E, Supplementary Table S17). We found that 84% of the CYP725A subfamily members (54 out of 64) and 10 key paclitaxel biosynthesis genes (such as TXS, T5αOH, T10βOH, T13αOH, T2αOH, T7βOH, DBAT, T9αOH, T1βOH, and TOT1) are predominantly clustered in specific regions on chr9, showing significant aggregation (Fig. 4D, Supplementary Table S18).

## Discussion

Gymnosperms, as a unique plant lineage, typically have very large genomes, often exceeding 1 billion base pairs per haploid genome, which poses significant challenges for genome assembly [9]. The large genome size not only increases the data requirements for sequencing but also results in a higher proportion of repetitive sequences and complex structural variations. These factors complicate the assembly process, increasing the error rate and uncertainty. Additionally, large genomes require more computational resources and more complex algorithms to handle the data. Therefore, despite advances in sequencing technology, the assembly of gymnosperm genomes remains a complex and arduous task. For example, the genomes of the gymnosperms *Cycas panzhihuaensis* and *Ginkgo biloba* are both in the range of 10–12 Gb and have over 70% repeat sequences [3, 70]. In this study, the repetitive sequences in *T. wallichiana* (TWv1) account for 84%. We overcame the obstacles of large genome size and high levels of repetitive elements to generate the first phased high-quality gymnosperm genome for *T. wallichiana*, providing valuable genomic resources for future gymnosperm research.

Dioecy is a major characteristic of gymnosperms, present in 667 out of 1,033 species (64.6%) [71]. Dioecy has evolved repeatedly from monoecy in gymnosperms, with 10–13 independent evolutions in the *Pinopsida* alone [72, 73]. Sex chromosomes have been studied in 6 species (0.6% of the total) in the genera *Cycas, Zamia, Stangeria, Ephedra, Podocarpus*, and *Ginkgo* [74]. In *Cycas revoluta*, males exhibit significant sex chromosome size differences, with the 22nd median chromosome being much shorter than the 21st submetacentric chromosome, while in females, both chromosomes are submetacentric and of equal length, showing an XX/XY type of sex determination [75]. Early studies on the sex determination of *G. biloba* reported both XY and ZW sex chromosome systems [76–80]. Therefore, further research is needed to clarify the exact sex determination system in *G. biloba*. In this study, we inferred the candidate sex chromosomes of *T. wallichiana* based on chromosome length and sex-biased expression data. Although we have made some preliminary progress, further in-depth research is needed to resolve the sex determination mechanism of *T. wallichiana*.

ODDs play an irreplaceable role as oxygenases widely involved in biosynthetic processes in plants [18], but their role in *T. wallichiana* is not yet fully understood. This study analyzed candidate ODDs screened from *T. wallichiana*. The experimental results demonstrated that certain ODD genes in *T. wallichiana* are capable of epoxidizing endotaxadiene. Molecular dynamics simulations further supported the catalytic roles of these genes in forming epoxides within the paclitaxel biosynthetic pathway. Whether ODDs can further promote the formation of the oxetane ring in taxane based on epoxides requires more in-depth research in the future. Cytochrome P450s (CYP450s) play crucial roles in the biosynthesis of the diterpene compound paclitaxel in the *Taxus* spp. However, the paclitaxel biosynthetic pathway and its enzymes are very complex, and some specific CYP450 enzymes remain unclear. Based on high-quality genomic data, this study conducted an in-depth analysis of the CYP450 gene family in *T. wallichiana*. We successfully identified and annotated several CYP450 genes potentially involved in the paclitaxel biosynthetic pathway. Not only did we clarify the gene clusters related to paclitaxel biosynthesis on the chromosomes, but we also identified potential candidate gene clusters. This achievement provides a solid foundation for subsequent verification of these genes’ precise roles in paclitaxel synthesis.

In recent years, many studies have conducted transcriptome-wide identification of CYP450s involved in terpenoid biosynthesis [81], including CYP716A47 related to ginsenoside biosynthesis [82], CYP76AH1 catalyzing the conversion of miltiradiene in tanshinone biosynthesis [83], and the recently identified CYP725A4, CYP725A37, and CYP725A55, which can catalyze the formation of the oxetane ring in taxane [15, 19, 20]. It is widely recognized that the *Taxus* spp. is the only large-scale source of paclitaxel. However, there has been long-standing controversy regarding whether other closely related species in gymnosperms (such as *T. grandis* and *Ginkgo*) can produce paclitaxel. Due to the difficulty of obtaining high-quality gymnosperm genomes, there has been a lack of genetic evidence to support existing experiments or hypotheses. In this study, collinearity analysis between the high-quality genome of *T. wallichiana* (TWv1) and the recently published genome of *T. grandis* provided evidence explaining why *T. grandis* produces little to no paclitaxel [69]. Furthermore, future comparative studies with other species from the same family and gymnosperms will provide a crucial foundation for understanding the genomic evolution and sex determination mechanisms in gymnosperms.

## Supplementary Material

giaf026_Supplemental_File

giaf026_GIGA-D-24-00293_Original_Submission

giaf026_GIGA-D-24-00293_Revision_1

giaf026_GIGA-D-24-00293_Revision_2

giaf026_Response_to_Reviewer_Comments_Original_Submission

giaf026_Response_to_Reviewer_Comments_Revision_1

giaf026_Reviewer_1_Report_Original_Submission Xuepeng Sun -- 9/4/2024

giaf026_Reviewer_1_Report_Revision_1 Xuepeng Sun -- 1/10/2025

giaf026_Reviewer_2_Report_Original_Submission Aleksey Zimin -- 9/11/2024

giaf026_Reviewer_2_Report_Revision_1 Aleksey Zimin -- 1/3/2025

## Data Availability

All sequencing data supporting the findings of this study, as well as the genome assemblies, are available at the National Center for Biotechnology Information (NCBI) under accession number PRJNA1146068. The metabolite detection data in this study are available at the NIH Common Fund’s National Metabolomics Data Repository (NMDR) website, the Metabolomics Workbench, where it has been assigned Project ID PR002330. The data can be accessed directly via their Project DOI [84]. All supporting data and materials are available in the *GigaScience* database, GigaDB [85].

## Additional Files

**Supplementary Fig. S1**. Collinear features of TWv1.

**Supplementary Fig. S2**. Genomic features of TWv1.

**Supplementary Fig. S3**. The yew sequenced in our study.

**Supplementary Fig. S4**. Gas chromatography–mass spectrometry (GC-MS) results.

**Supplementary Table S1**. Twenty-three other Gymnospermae genomes used in Fig. 1C.

**Supplementary Table S2**. Summary of sequencing data.

**Supplementary Table S3**. Summary of HiFi sequencing data.

**Supplementary Table S4**. Summary of nanopore sequencing data.

**Supplementary Table S5**. Gap status of Twv1.

**Supplementary Table S6**. Assembly quality of TWv1.

**Supplementary Table S7**. BUSCO analysis of TWv1 genome completeness.

**Supplementary Table S8**. Survey results of telomeric sequence.

**Supplementary Table S9**. Gene annotation.

**Supplementary Table S10**. Summary of transposable elements.

**Supplementary Table S11**. Structural variation (SV) in the Twv1 genome.

**Supplementary Table S12**. Structural variation (SV) statistics.

**Supplementary Table S13**. Large-scale chromosomal structural variation.

**Supplementary Table S14**. Collapse status of Twv1.

**Supplementary Table S15**. Distribution and quantity of major ODDs in Twv1.

**Supplementary Table S16**. Eleven sequences selected through coexpression network analysis.

**Supplementary Table S17**. Statistical analysis of CYP450s in Twv1.

**Supplementary Table S18**. Mapping of characterized enzymes in the biosynthetic pathway of paclitaxel on the Himalayan yew genome.

## Abbreviations

BLAST: Basic Local Alignment Search Tool; bp: base pairs; BUSCO: Benchmarking Universal Single-Copy Orthologs; CDF2: cycling Dof factor 2; CDS: coding sequence; CYP450: cytochrome P450 enzyme; Embryophyta_odb10: Embryophyta OrthoDB v10 (a database of orthologous genes for land plants); Gb: gigabase pairs; gDNA: genomic DNA; GO: Gene Ontology; Gymnosperm_odb10: Gymnosperm OrthoDB v10 (a database of orthologous genes for gymnosperms); Hi-C: high-throughput chromosome conformation capture; Kb: kilobase pairs; KEGG: Kyoto Encyclopedia of Genes and Genomes; LINE: long interspersed nuclear element; LTR: long terminal repeat; Mbp/Mb: megabase pairs; Mya: million years ago; Nr: NCBI’s nonredundant database; ODD: 2-oxoglutarate/Fe(II)-dependent dioxygenase; ODD: 2-oxoglutarate/Fe(II)-dependent dioxygenase; ONT UL: Oxford Nanopore Technologies Ultra Long; PacBio HiFi: Pacific Biosciences high fidelity; QV: quality value; RNA-seq: RNA sequencing; SINE: short interspersed nuclear element; SNP: single nucleotide polymorphism; SNR: signal-to-noise ratio; snRNA: small nuclear RNA; SV: structural variation; T2T: telomere to telomere; TE: transposable element; TRF: tandem repeat; UniProtKB/Swiss-Prot: Universal Protein Knowledgebase/Swiss-Prot; UniProtKB/TrEMBL: Universal Protein Knowledgebase/Translated European Molecular Biology Laboratory.
