# Supplementary material for: Phased high-quality genome of the gymnosperm Himalayan Yew assists in paclitaxel pathway exploration
Source: Gigascience. 2025 Apr 4;14:giaf026. doi: 10.1093/gigascience/giaf026 (PMC11970372; doi:10.1093/gigascience/giaf026)
Supplement: giaf026_Supplemental_File [file giaf026_supplemental_file.docx]

**Supplementary Information for the Paper Entitled**

**Phased High-Quality Genome of the Gymnosperm Himalayan yew Assists in Paclitaxel Pathway Exploration**

Zhenzhu Li^1, ‡^, Hang Zong^1, ‡^, Xiaonan Liu^2, 8, ‡^, Xiao Wang^2, 3^, Shimeng Liu^3^, Xi Jiao^3^, Xianqing Chen^3^, Hao Wu^3^, Zhuoya Liu^1^, Zhongkai Wang^1^, Yongqiang Wang^7^, Yi Liu^1^, Botong Zhou^1^, Zihe Li^1^,Qiuhui Du^3^, Jing Li^2^, Jian Cheng^2^, Jie Bai^2^, Xiaoxi Zhu^2^, Yue Yang^4^, Guichun Liu^5^, Li Zhang^6^, Huifeng Jiang^2, *^ and Wen Wang^1,*^

**Table of Contents**

**1. Supplementary Figures for the Main Text (Pages 2 - 5)**

**2. Supplementary Tables for the Main Text (Pages 6 - 43)**

**3. Reference for the Supplementary Information (Pages 44 - 45)**

**1. Supplementary Figures for the Main Text**


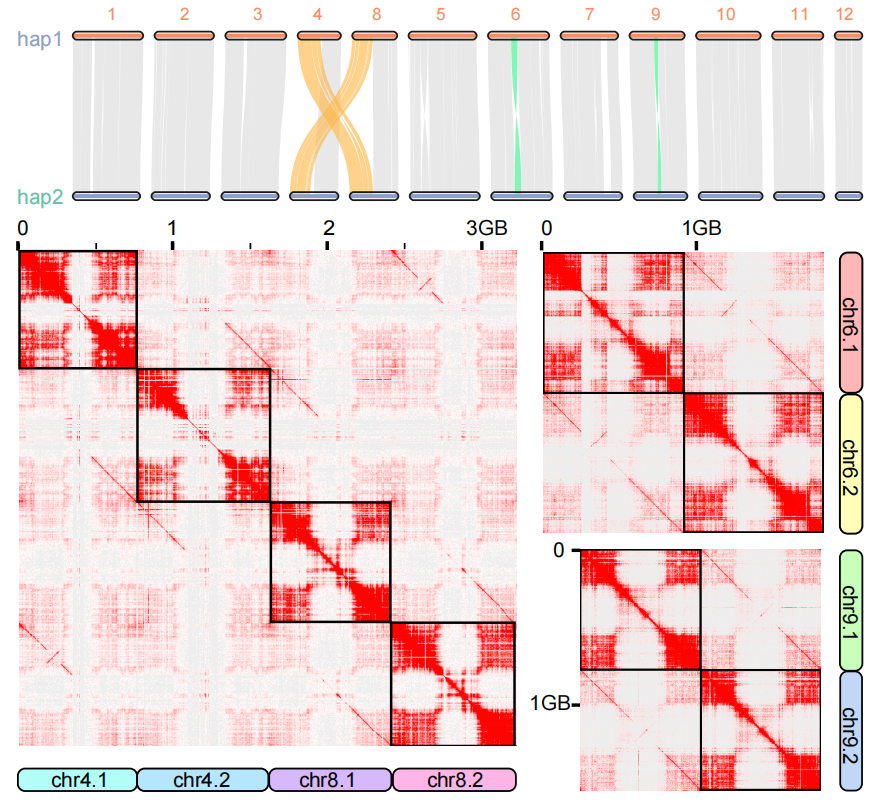


**Figure S1. Collinear Features of TWv1.** (A) Collinearity between TWv1-hap1 and TWv1-hap2. Gray lines represent Collinear regions between chromosomes, orange lines indicate translocations of chromosomal segments, and green lines indicate inversions of chromosomal segments. (B) Hi-C heatmap evidence of Collinearity within the TWv1 genome.


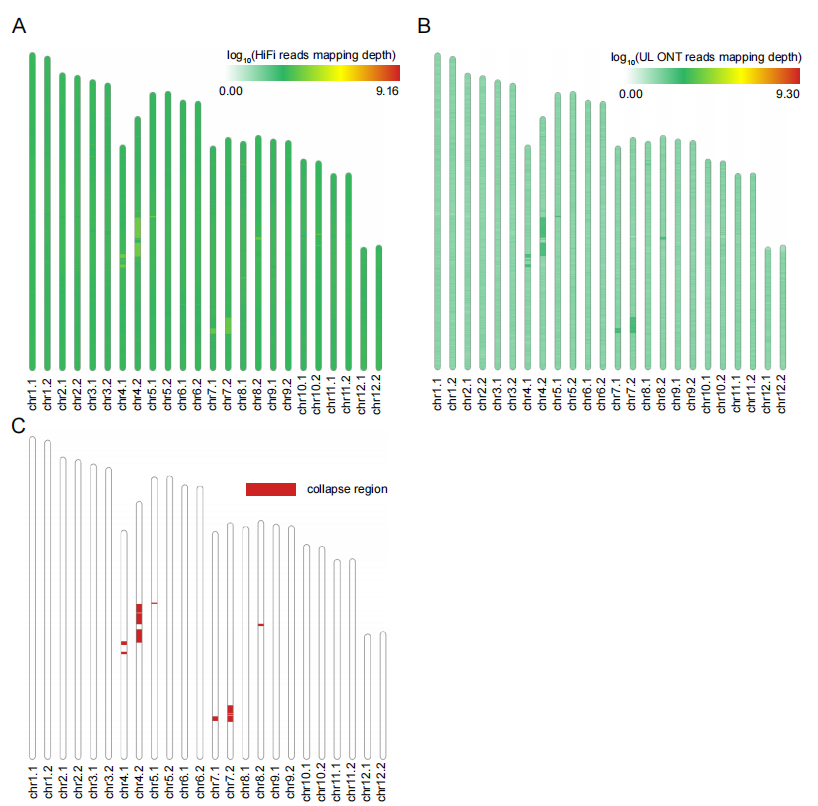


**Figure S2. Genomic Features of TWv1.** (A) Log10-transformed mapping depth of HIFI reads. (B) Log10-transformed mapping depth of UL ONT reads. (C) Collapsed regions in TWv1, highlighted in red.

**
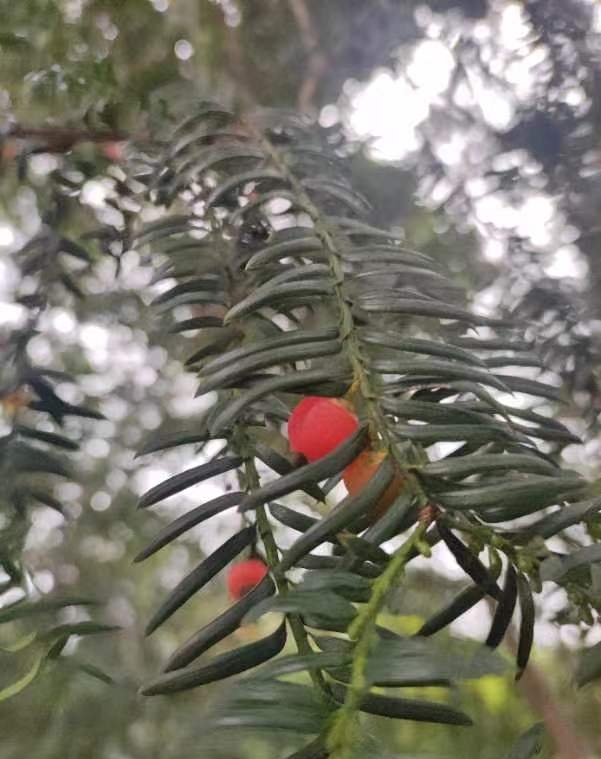
**

**Figure S3. The yew sequenced in our study.**


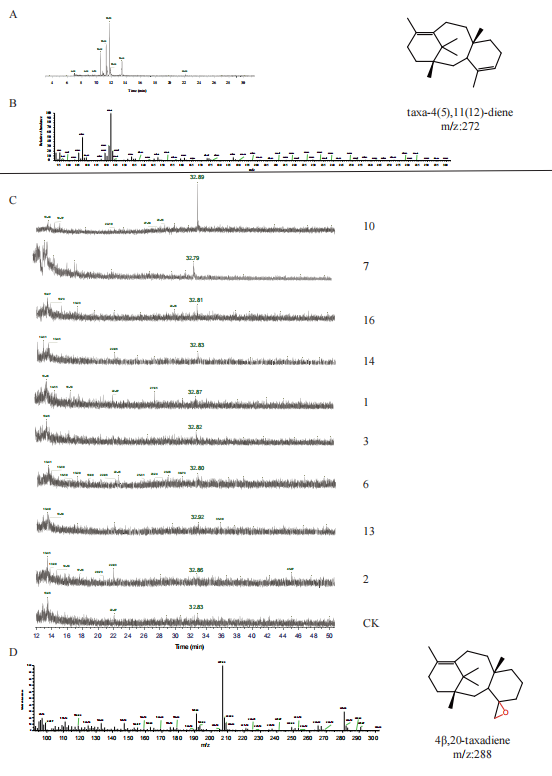


**Figure S4.** **Gas Chromatography-Mass Spectrometry (GC-MS) Results.**

(A) Ion chromatogram of the substrate taxa-4(5),11(12)-diene. (B) Mass spectrum of the substrate taxa-4(5),11(12)-diene. (C) Ion chromatogram of the product 4β,20-taxadiene. (D) Mass spectrum of the product 4β,20-taxadiene.

**2. Supplementary Tables for the Main Text**

**Table S1.** 23 other Gymnospermae genomes used in figure 1C.

| **Species name** | **Reference** |
| --- | --- |
| *Abies_alba* | [1] |
| *Ginkgo_biloba_2* | [2] |
| *Ginkgo_biloba* | [3] |
| *Gnetum_montanum* | [4] |
| *Larix_sibirica* | [5] |
| *Metasequoia_glyptostroboides* | [6] |
| *Picea_abies* | [7] |
| *Picea_engelmannii* | [8] |
| *Picea_glauca* | [9] |
| *Picea_mariana* | [10] |
| *Picea_sitchensis* | [8] |
| *Pinus_lambertiana* | [11] |
| *Pinus_tabuliformis* | [12] |
| *Pinus_taeda* | [13] |
| *Pseudotsuga_menziesii* | [14] |
| *Sequoia_sempervirens* | [15] |
| *TCv0* | [16] |
| *Thuja_plicata* | [17] |
| *Torreya_grandis* | [18] |
| *TWv0* | [19] |
| *TWv1* | this study |
| *TYv0* | [20] |
| *Welwitschia_mirabilis_2* | [21] |
| *Welwitschia_mirabilis* | [22] |

**Table S2.** Summary of sequencing data.

| **Libraries** | **Insert size** | **Read length N50 (bp)** | **Total data (Gb)** | **Depth (x)** |
| --- | --- | --- | --- | --- |
|  |  |  |  |  |
| PacBio HiFi | 15 kb | 16265 | 722 | 72 |
| Nanopore | / | 53976 | 875 | 88 |

**Table S3.** Summary of hifi sequencing data.

| **Library number** | **Total data (bp)** | **Read length N50 (bp)** | **Read number** |
| --- | --- | --- | --- |
| cell1 | 70,025,524,021 | 15,945 | 4,413,964 |
| cell2 | 85,154,916,446 | 19,048 | 4,459,639 |
| cell3 | 41,562,239,177 | 18,298 | 2,274,823 |
| cell4 | 73,873,654,397 | 16,489 | 4,524,868 |
| cell5 | 85,205,629,573 | 16,406 | 6,112,210 |
| cell6 | 84,213,819,745 | 16,522 | 5,060,748 |
| cell7 | 81,902,718,347 | 16,538 | 4,897,318 |
| cell8 | 94,518,058,140 | 14,287 | 6,616,884 |
| cell9 | 106,010,403,455 | 14,514 | 7,329,045 |

**Table S4.** Summary of nanopore sequencing data.

| **Library number** | **Total data (bp)** | **Read length N50 (bp)** | **Read number** |
| --- | --- | --- | --- |
| cell1 | 48,123,372,730 | 59,272 | 1,337,656 |
| cell2 | 60,546,217,439 | 50,041 | 1,885,712 |
| cell3 | 69,951,845,189 | 49,838 | 2,377,694 |
| cell4 | 76,572,772,776 | 50,017 | 2,606,725 |
| cell5 | 69,923,991,668 | 47,316 | 2,497,287 |
| cell6 | 43,711,342,556 | 48,374 | 1,447,026 |
| cell7 | 19,883,945,598 | 53,347 | 585,815 |
| cell8 | 41,749,520,081 | 57,883 | 1,258,319 |
| cell9 | 44,278,426,489 | 62,947 | 1,153,554 |
| cell10 | 42,678,646,570 | 64,285 | 1,089,440 |
| cell11 | 36,228,536,270 | 55,682 | 932,285 |
| cell12 | 42,300,643,634 | 59,277 | 987,264 |
| cell13 | 26,863,032,123 | 60,649 | 622,980 |
| cell14 | 29,490,589,438 | 59,228 | 685,275 |
| cell15 | 37,760,947,624 | 58,949 | 875,273 |
| cell16 | 35,856,168,309 | 52,758 | 987,017 |
| cell17 | 33,184,964,426 | 53,885 | 905,079 |
| cell18 | 35,172,333,651 | 57,965 | 845,750 |
| cell19 | 39,611,564,274 | 41,747 | 2,190,937 |
| cell20 | 41,367,654,493 | 55,611 | 1,032,098 |

**Table S5.** Gap status of Twv1.

| **Chr id** | **Gap start** | **Gap end** | **Closed or not closed** |
| --- | --- | --- | --- |
| chr1.1 | 64,727,929 | 64,728,428 | not closed |
| chr1.1 | 65,307,353 | 65,307,852 | not closed |
| chr1.1 | 419,108,043 | 419,108,542 | not closed |
| chr1.1 | 678,304,514 | 678,305,013 | not closed |
| chr1.1 | 800,661,811 | 800,662,310 | not closed |
| chr1.1 | 965,471,011 | 965,471,510 | not closed |
| chr1.1 | 1,024,206,216 | 1,024,206,715 | not closed |
| chr1.2 | 312,151,531 | 312,152,030 | not closed |
| chr1.2 | 348,147,843 | 348,148,342 | not closed |
| chr1.2 | 514,385,288 | 514,385,787 | not closed |
| chr1.2 | 599,926,837 | 599,927,336 | not closed |
| chr1.2 | 644,710,813 | 644,711,312 | not closed |
| chr1.2 | 656,605,361 | 656,605,860 | not closed |
| chr1.2 | 657,479,187 | 657,479,686 | not closed |
| chr1.2 | 659,537,629 | 659,538,128 | not closed |
| chr1.2 | 755,987,665 | 755,988,164 | not closed |
| chr1.2 | 854,710,770 | 854,711,269 | not closed |
| chr1.2 | 933,350,736 | 933,351,235 | not closed |
| chr1.2 | 957,011,533 | 957,012,032 | not closed |
| chr1.2 | 966,836,227 | 966,836,726 | not closed |
| chr2.2 | 104,857,404 | 104,857,903 | not closed |
| chr2.2 | 336,546,328 | 336,546,827 | not closed |
| chr2.2 | 659,991,920 | 659,992,419 | not closed |
| chr2.2 | 667,133,509 | 667,134,008 | not closed |
| chr2.2 | 667,557,751 | 667,558,250 | not closed |
| chr2.2 | 686,985,867 | 686,986,366 | not closed |
| chr2.2 | 722,138,030 | 722,138,529 | not closed |
| chr2.2 | 727,429,675 | 727,430,174 | not closed |
| chr2.2 | 754,245,154 | 754,245,653 | not closed |
| chr3.1 | 18,012,509 | 18,013,008 | not closed |
| chr3.1 | 92,414,208 | 92,414,707 | not closed |
| chr3.1 | 118,451,472 | 118,451,971 | not closed |
| chr3.1 | 330,849,151 | 330,849,650 | not closed |
| chr3.1 | 478,419,117 | 478,419,616 | not closed |
| chr3.1 | 621,588,873 | 621,589,372 | not closed |
| chr3.1 | 632,047,829 | 632,048,328 | not closed |
| chr3.1 | 901,110,728 | 901,111,227 | not closed |
| chr3.1 | 981,125,026 | 981,125,525 | not closed |
| chr3.2 | 128,475,907 | 128,476,406 | not closed |
| chr3.2 | 201,551,755 | 201,552,254 | not closed |
| chr3.2 | 228,427,847 | 228,428,346 | not closed |
| chr3.2 | 260,962,321 | 260,962,820 | not closed |
| chr3.2 | 352,909,412 | 352,909,911 | not closed |
| chr3.2 | 616,720,458 | 616,720,957 | not closed |
| chr3.2 | 743,494,077 | 743,494,576 | not closed |
| chr3.2 | 763,146,846 | 763,147,345 | not closed |
| chr3.2 | 970,019,926 | 970,020,425 | not closed |
| chr4.1 | 17,849,259 | 17,849,758 | not closed |
| chr4.1 | 325,667,183 | 325,667,682 | not closed |
| chr4.1 | 352,592,921 | 352,593,420 | not closed |
| chr4.1 | 364,072,276 | 364,072,775 | not closed |
| chr4.1 | 369,472,700 | 369,473,199 | not closed |
| chr4.1 | 373,005,914 | 373,006,413 | not closed |
| chr4.1 | 553,635,527 | 553,636,026 | not closed |
| chr4.1 | 554,555,582 | 554,556,081 | not closed |
| chr4.2 | 42,446,002 | 42,446,501 | not closed |
| chr4.2 | 48,037,637 | 48,038,136 | not closed |
| chr4.2 | 69,038,137 | 69,038,636 | not closed |
| chr4.2 | 78,332,584 | 78,333,083 | not closed |
| chr4.2 | 114,220,258 | 114,220,757 | not closed |
| chr4.2 | 477,152,908 | 477,153,407 | not closed |
| chr4.2 | 498,092,191 | 498,092,690 | not closed |
| chr4.2 | 509,509,974 | 509,510,473 | not closed |
| chr4.2 | 514,252,647 | 514,253,146 | not closed |
| chr4.2 | 652,046,445 | 652,046,944 | not closed |
| chr4.2 | 734,413,780 | 734,414,279 | not closed |
| chr4.2 | 796,217,095 | 796,217,594 | not closed |
| chr5.1 | 62,873 | 63,372 | not closed |
| chr5.1 | 19,423,792 | 19,424,291 | not closed |
| chr5.1 | 55,998,008 | 55,998,507 | not closed |
| chr5.1 | 281,281,029 | 281,281,528 | not closed |
| chr5.1 | 284,016,799 | 284,017,298 | not closed |
| chr5.1 | 545,419,870 | 545,420,369 | not closed |
| chr5.1 | 634,046,314 | 634,046,813 | not closed |
| chr5.1 | 641,142,888 | 641,143,387 | not closed |
| chr5.1 | 645,767,102 | 645,767,601 | not closed |
| chr5.1 | 646,713,914 | 646,714,413 | not closed |
| chr5.1 | 653,179,037 | 653,179,536 | not closed |
| chr5.1 | 674,580,237 | 674,580,736 | not closed |
| chr5.1 | 878,131,632 | 878,132,131 | not closed |
| chr5.2 | 34,741,641 | 34,742,140 | not closed |
| chr5.2 | 204,606,596 | 204,607,095 | not closed |
| chr5.2 | 230,070,367 | 230,070,866 | not closed |
| chr5.2 | 274,322,824 | 274,323,323 | not closed |
| chr5.2 | 372,099,020 | 372,099,519 | not closed |
| chr5.2 | 417,294,885 | 417,295,384 | not closed |
| chr5.2 | 491,172,741 | 491,173,240 | not closed |
| chr5.2 | 536,093,998 | 536,094,497 | not closed |
| chr5.2 | 620,218,989 | 620,219,488 | not closed |
| chr5.2 | 730,273,725 | 730,274,224 | not closed |
| chr5.2 | 843,579,597 | 843,580,096 | not closed |
| chr6.1 | 102,708,889 | 102,709,388 | not closed |
| chr6.1 | 194,346,407 | 194,346,906 | not closed |
| chr6.1 | 223,490,612 | 223,491,111 | not closed |
| chr6.1 | 335,374,036 | 335,374,535 | not closed |
| chr6.1 | 361,459,511 | 361,460,010 | not closed |
| chr6.1 | 754,945,278 | 754,945,777 | not closed |
| chr6.2 | 159,220,348 | 159,220,847 | not closed |
| chr6.2 | 218,071,714 | 218,072,213 | not closed |
| chr6.2 | 423,104,828 | 423,105,327 | not closed |
| chr6.2 | 522,136,434 | 522,136,933 | not closed |
| chr6.2 | 542,438,551 | 542,439,050 | not closed |
| chr6.2 | 711,790,286 | 711,790,785 | not closed |
| chr6.2 | 751,553,678 | 751,554,177 | not closed |
| chr6.2 | 757,682,373 | 757,682,872 | not closed |
| chr7.1 | 149,310,915 | 149,311,414 | not closed |
| chr7.1 | 197,958,184 | 197,958,683 | not closed |
| chr7.1 | 412,309,180 | 412,309,679 | not closed |
| chr7.1 | 507,777,090 | 507,777,589 | not closed |
| chr7.1 | 611,187,241 | 611,187,740 | not closed |
| chr7.1 | 617,912,699 | 617,913,198 | not closed |
| chr7.1 | 619,695,049 | 619,695,548 | not closed |
| chr7.1 | 621,431,209 | 621,431,708 | not closed |
| chr7.1 | 638,385,929 | 638,386,428 | not closed |
| chr7.1 | 639,580,004 | 639,580,503 | not closed |
| chr7.1 | 639,620,727 | 639,621,226 | not closed |
| chr7.2 | 169,594,409 | 169,594,908 | not closed |
| chr7.2 | 337,211,208 | 337,211,707 | not closed |
| chr7.2 | 337,248,714 | 337,249,213 | not closed |
| chr7.2 | 429,828,528 | 429,829,027 | not closed |
| chr7.2 | 526,046,074 | 526,046,573 | not closed |
| chr7.2 | 543,649,987 | 543,650,486 | not closed |
| chr7.2 | 646,339,541 | 646,340,040 | not closed |
| chr7.2 | 711,586,016 | 711,586,515 | not closed |
| chr8.1 | 49,197,848 | 49,198,347 | not closed |
| chr8.1 | 67,448,480 | 67,448,979 | not closed |
| chr8.1 | 80,348,980 | 80,349,479 | not closed |
| chr8.1 | 350,105,135 | 350,105,634 | not closed |
| chr8.1 | 501,474,594 | 501,475,093 | not closed |
| chr8.1 | 692,536,335 | 692,536,834 | not closed |
| chr8.2 | 67,380,581 | 67,381,080 | not closed |
| chr8.2 | 224,680,533 | 224,681,032 | not closed |
| chr8.2 | 230,829,966 | 230,830,465 | not closed |
| chr8.2 | 230,863,490 | 230,863,989 | not closed |
| chr8.2 | 265,457,816 | 265,458,315 | not closed |
| chr8.2 | 273,298,233 | 273,298,732 | not closed |
| chr8.2 | 320,217,586 | 320,218,085 | not closed |
| chr8.2 | 346,921,533 | 346,922,032 | not closed |
| chr8.2 | 579,425,746 | 579,426,245 | not closed |
| chr8.2 | 678,680,887 | 678,681,386 | not closed |
| chr8.2 | 733,950,446 | 733,950,945 | not closed |
| chr8.2 | 755,749,496 | 755,749,995 | not closed |
| chr9.1 | 383,658,068 | 383,658,567 | not closed |
| chr9.1 | 609,084,097 | 609,084,596 | not closed |
| chr9.1 | 658,574,721 | 658,575,220 | not closed |
| chr9.1 | 749,966,373 | 749,966,872 | not closed |
| chr9.2 | 52,381,343 | 52,381,842 | not closed |
| chr9.2 | 59,393,180 | 59,393,679 | not closed |
| chr9.2 | 73,499,855 | 73,500,354 | not closed |
| chr9.2 | 139,146,098 | 139,146,597 | not closed |
| chr9.2 | 164,762,223 | 164,762,722 | not closed |
| chr9.2 | 187,363,584 | 187,364,083 | not closed |
| chr9.2 | 555,645,643 | 555,646,142 | not closed |
| chr9.2 | 556,101,774 | 556,102,273 | not closed |
| chr9.2 | 556,160,842 | 556,161,341 | not closed |
| chr9.2 | 602,261,537 | 602,262,036 | not closed |
| chr9.2 | 650,339,228 | 650,339,727 | not closed |
| chr9.2 | 650,586,641 | 650,587,140 | not closed |
| chr9.2 | 702,994,732 | 702,995,231 | not closed |
| chr10.1 | 63,746,282 | 63,746,781 | not closed |
| chr10.1 | 82,813,145 | 82,813,644 | not closed |
| chr10.1 | 144,835,204 | 144,835,703 | not closed |
| chr10.1 | 154,360,917 | 154,361,416 | not closed |
| chr10.1 | 159,340,809 | 159,341,308 | not closed |
| chr10.1 | 248,805,037 | 248,805,536 | not closed |
| chr10.1 | 248,969,605 | 248,970,104 | not closed |
| chr10.1 | 249,070,454 | 249,070,953 | not closed |
| chr10.1 | 333,055,583 | 333,056,082 | not closed |
| chr10.1 | 403,509,566 | 403,510,065 | not closed |
| chr10.1 | 623,295,734 | 623,296,233 | not closed |
| chr10.1 | 660,296,963 | 660,297,462 | not closed |
| chr10.1 | 702,836,035 | 702,836,534 | not closed |
| chr10.2 | 46,263,314 | 46,263,813 | not closed |
| chr10.2 | 66,333,234 | 66,333,733 | not closed |
| chr10.2 | 85,051,872 | 85,052,371 | not closed |
| chr10.2 | 147,903,105 | 147,903,604 | not closed |
| chr10.2 | 251,694,638 | 251,695,137 | not closed |
| chr10.2 | 330,177,182 | 330,177,681 | not closed |
| chr10.2 | 334,138,816 | 334,139,315 | not closed |
| chr10.2 | 372,658,184 | 372,658,683 | not closed |
| chr10.2 | 412,211,615 | 412,212,114 | not closed |
| chr10.2 | 544,403,642 | 544,404,141 | not closed |
| chr10.2 | 681,031,760 | 681,032,259 | not closed |
| chr11.1 | 183,701,263 | 183,701,762 | not closed |
| chr11.1 | 198,626,829 | 198,627,328 | not closed |
| chr11.1 | 433,790,293 | 433,790,792 | not closed |
| chr11.1 | 441,142,206 | 441,142,705 | not closed |
| chr11.1 | 465,703,215 | 465,703,714 | not closed |
| chr11.1 | 666,601,782 | 666,602,281 | not closed |
| chr11.2 | 53,433,599 | 53,434,098 | not closed |
| chr11.2 | 74,527,822 | 74,528,321 | not closed |
| chr11.2 | 92,496,727 | 92,497,226 | not closed |
| chr11.2 | 135,812,868 | 135,813,367 | not closed |
| chr11.2 | 167,015,659 | 167,016,158 | not closed |
| chr11.2 | 402,901,702 | 402,902,201 | not closed |
| chr11.2 | 436,127,716 | 436,128,215 | not closed |
| chr12.1 | 33,073,609 | 33,074,108 | not closed |
| chr12.1 | 253,011,505 | 253,012,004 | not closed |
| chr12.2 | 210,641,132 | 210,641,631 | not closed |
| chr12.2 | 237,222,008 | 237,222,507 | not closed |
| chr12.2 | 327,631,374 | 327,631,873 | not closed |
| chr1.1 | 64,727,929 | 64,728,428 | not closed |
| chr1.1 | 65,307,353 | 65,307,852 | not closed |
| chr1.1 | 419,108,043 | 419,108,542 | not closed |
| chr1.1 | 678,304,514 | 678,305,013 | not closed |
| chr1.1 | 800,661,811 | 800,662,310 | not closed |
| chr1.1 | 965,471,011 | 965,471,510 | not closed |
| chr1.1 | 1,024,206,216 | 1,024,206,715 | not closed |
| chr1.2 | 312,151,531 | 312,152,030 | not closed |
| chr1.2 | 348,147,843 | 348,148,342 | not closed |
| chr1.2 | 514,385,288 | 514,385,787 | not closed |
| chr1.2 | 599,926,837 | 599,927,336 | not closed |
| chr1.2 | 644,710,813 | 644,711,312 | not closed |
| chr1.2 | 656,605,361 | 656,605,860 | not closed |
| chr1.2 | 657,479,187 | 657,479,686 | not closed |
| chr1.2 | 659,537,629 | 659,538,128 | not closed |
| chr1.2 | 755,987,665 | 755,988,164 | not closed |
| chr1.2 | 854,710,770 | 854,711,269 | not closed |
| chr1.2 | 933,350,736 | 933,351,235 | not closed |
| chr1.2 | 957,011,533 | 957,012,032 | not closed |
| chr1.2 | 966,836,227 | 966,836,726 | not closed |
| chr2.2 | 104,857,404 | 104,857,903 | not closed |
| chr2.2 | 336,546,328 | 336,546,827 | not closed |
| chr2.2 | 659,991,920 | 659,992,419 | not closed |
| chr2.2 | 667,133,509 | 667,134,008 | not closed |
| chr2.2 | 667,557,751 | 667,558,250 | not closed |
| chr2.2 | 686,985,867 | 686,986,366 | not closed |
| chr2.2 | 722,138,030 | 722,138,529 | not closed |
| chr2.2 | 727,429,675 | 727,430,174 | not closed |
| chr2.2 | 754,245,154 | 754,245,653 | not closed |
| chr3.1 | 18,012,509 | 18,013,008 | not closed |
| chr3.1 | 92,414,208 | 92,414,707 | not closed |
| chr3.1 | 118,451,472 | 118,451,971 | not closed |
| chr3.1 | 330,849,151 | 330,849,650 | not closed |
| chr3.1 | 478,419,117 | 478,419,616 | not closed |
| chr3.1 | 621,588,873 | 621,589,372 | not closed |
| chr3.1 | 632,047,829 | 632,048,328 | not closed |
| chr3.1 | 901,110,728 | 901,111,227 | not closed |
| chr3.1 | 981,125,026 | 981,125,525 | not closed |
| chr3.2 | 128,475,907 | 128,476,406 | not closed |
| chr3.2 | 201,551,755 | 201,552,254 | not closed |
| chr3.2 | 228,427,847 | 228,428,346 | not closed |
| chr3.2 | 260,962,321 | 260,962,820 | not closed |
| chr3.2 | 352,909,412 | 352,909,911 | not closed |
| chr3.2 | 616,720,458 | 616,720,957 | not closed |
| chr3.2 | 743,494,077 | 743,494,576 | not closed |
| chr3.2 | 763,146,846 | 763,147,345 | not closed |
| chr3.2 | 970,019,926 | 970,020,425 | not closed |
| chr4.1 | 17,849,259 | 17,849,758 | not closed |
| chr4.1 | 325,667,183 | 325,667,682 | not closed |
| chr4.1 | 352,592,921 | 352,593,420 | not closed |
| chr4.1 | 364,072,276 | 364,072,775 | not closed |
| chr4.1 | 369,472,700 | 369,473,199 | not closed |
| chr4.1 | 373,005,914 | 373,006,413 | not closed |
| chr4.1 | 553,635,527 | 553,636,026 | not closed |
| chr4.1 | 554,555,582 | 554,556,081 | not closed |
| chr4.2 | 42,446,002 | 42,446,501 | not closed |
| chr4.2 | 48,037,637 | 48,038,136 | not closed |
| chr4.2 | 69,038,137 | 69,038,636 | not closed |
| chr4.2 | 78,332,584 | 78,333,083 | not closed |
| chr4.2 | 114,220,258 | 114,220,757 | not closed |
| chr4.2 | 477,152,908 | 477,153,407 | not closed |
| chr4.2 | 498,092,191 | 498,092,690 | not closed |
| chr4.2 | 509,509,974 | 509,510,473 | not closed |
| chr4.2 | 514,252,647 | 514,253,146 | not closed |
| chr4.2 | 652,046,445 | 652,046,944 | not closed |
| chr4.2 | 734,413,780 | 734,414,279 | not closed |
| chr4.2 | 796,217,095 | 796,217,594 | not closed |
| chr5.1 | 62,873 | 63,372 | not closed |
| chr5.1 | 19,423,792 | 19,424,291 | not closed |
| chr5.1 | 55,998,008 | 55,998,507 | not closed |
| chr5.1 | 281,281,029 | 281,281,528 | not closed |
| chr5.1 | 284,016,799 | 284,017,298 | not closed |
| chr5.1 | 545,419,870 | 545,420,369 | not closed |
| chr5.1 | 634,046,314 | 634,046,813 | not closed |
| chr5.1 | 641,142,888 | 641,143,387 | not closed |
| chr5.1 | 645,767,102 | 645,767,601 | not closed |
| chr5.1 | 646,713,914 | 646,714,413 | not closed |
| chr5.1 | 653,179,037 | 653,179,536 | not closed |
| chr5.1 | 674,580,237 | 674,580,736 | not closed |
| chr5.1 | 878,131,632 | 878,132,131 | not closed |
| chr5.2 | 34,741,641 | 34,742,140 | not closed |
| chr5.2 | 204,606,596 | 204,607,095 | not closed |
| chr5.2 | 230,070,367 | 230,070,866 | not closed |
| chr5.2 | 274,322,824 | 274,323,323 | not closed |
| chr5.2 | 372,099,020 | 372,099,519 | not closed |
| chr5.2 | 417,294,885 | 417,295,384 | not closed |
| chr5.2 | 491,172,741 | 491,173,240 | not closed |
| chr5.2 | 536,093,998 | 536,094,497 | not closed |
| chr5.2 | 620,218,989 | 620,219,488 | not closed |
| chr5.2 | 730,273,725 | 730,274,224 | not closed |
| chr5.2 | 843,579,597 | 843,580,096 | not closed |
| chr6.1 | 102,708,889 | 102,709,388 | not closed |
| chr6.1 | 194,346,407 | 194,346,906 | not closed |
| chr6.1 | 223,490,612 | 223,491,111 | not closed |
| chr6.1 | 335,374,036 | 335,374,535 | not closed |
| chr6.1 | 361,459,511 | 361,460,010 | not closed |
| chr6.1 | 754,945,278 | 754,945,777 | not closed |
| chr6.2 | 159,220,348 | 159,220,847 | not closed |
| chr6.2 | 218,071,714 | 218,072,213 | not closed |
| chr6.2 | 423,104,828 | 423,105,327 | not closed |
| chr6.2 | 522,136,434 | 522,136,933 | not closed |
| chr6.2 | 542,438,551 | 542,439,050 | not closed |
| chr6.2 | 711,790,286 | 711,790,785 | not closed |
| chr6.2 | 751,553,678 | 751,554,177 | not closed |
| chr6.2 | 757,682,373 | 757,682,872 | not closed |
| chr7.1 | 149,310,915 | 149,311,414 | not closed |
| chr7.1 | 197,958,184 | 197,958,683 | not closed |
| chr7.1 | 412,309,180 | 412,309,679 | not closed |
| chr7.1 | 507,777,090 | 507,777,589 | not closed |
| chr7.1 | 611,187,241 | 611,187,740 | not closed |
| chr7.1 | 617,912,699 | 617,913,198 | not closed |
| chr7.1 | 619,695,049 | 619,695,548 | not closed |
| chr7.1 | 621,431,209 | 621,431,708 | not closed |
| chr7.1 | 638,385,929 | 638,386,428 | not closed |
| chr7.1 | 639,580,004 | 639,580,503 | not closed |
| chr7.1 | 639,620,727 | 639,621,226 | not closed |
| chr7.2 | 169,594,409 | 169,594,908 | not closed |
| chr7.2 | 337,211,208 | 337,211,707 | not closed |
| chr7.2 | 337,248,714 | 337,249,213 | not closed |
| chr7.2 | 429,828,528 | 429,829,027 | not closed |
| chr7.2 | 526,046,074 | 526,046,573 | not closed |
| chr7.2 | 543,649,987 | 543,650,486 | not closed |
| chr7.2 | 646,339,541 | 646,340,040 | not closed |
| chr7.2 | 711,586,016 | 711,586,515 | not closed |
| chr8.1 | 49,197,848 | 49,198,347 | not closed |
| chr8.1 | 67,448,480 | 67,448,979 | not closed |
| chr8.1 | 80,348,980 | 80,349,479 | not closed |
| chr8.1 | 350,105,135 | 350,105,634 | not closed |
| chr8.1 | 501,474,594 | 501,475,093 | not closed |
| chr8.1 | 692,536,335 | 692,536,834 | not closed |
| chr8.2 | 67,380,581 | 67,381,080 | not closed |
| chr8.2 | 224,680,533 | 224,681,032 | not closed |
| chr8.2 | 230,829,966 | 230,830,465 | not closed |
| chr8.2 | 230,863,490 | 230,863,989 | not closed |
| chr8.2 | 265,457,816 | 265,458,315 | not closed |
| chr8.2 | 273,298,233 | 273,298,732 | not closed |
| chr8.2 | 320,217,586 | 320,218,085 | not closed |
| chr8.2 | 346,921,533 | 346,922,032 | not closed |
| chr8.2 | 579,425,746 | 579,426,245 | not closed |
| chr8.2 | 678,680,887 | 678,681,386 | not closed |
| chr8.2 | 733,950,446 | 733,950,945 | not closed |
| chr8.2 | 755,749,496 | 755,749,995 | not closed |
| chr9.1 | 383,658,068 | 383,658,567 | not closed |
| chr9.1 | 609,084,097 | 609,084,596 | not closed |
| chr9.1 | 658,574,721 | 658,575,220 | not closed |
| chr9.1 | 749,966,373 | 749,966,872 | not closed |
| chr9.2 | 52,381,343 | 52,381,842 | not closed |
| chr9.2 | 59,393,180 | 59,393,679 | not closed |
| chr9.2 | 73,499,855 | 73,500,354 | not closed |
| chr9.2 | 139,146,098 | 139,146,597 | not closed |
| chr9.2 | 164,762,223 | 164,762,722 | not closed |
| chr9.2 | 187,363,584 | 187,364,083 | not closed |
| chr9.2 | 555,645,643 | 555,646,142 | not closed |
| chr9.2 | 556,101,774 | 556,102,273 | not closed |
| chr9.2 | 556,160,842 | 556,161,341 | not closed |
| chr9.2 | 602,261,537 | 602,262,036 | not closed |
| chr9.2 | 650,339,228 | 650,339,727 | not closed |
| chr9.2 | 650,586,641 | 650,587,140 | not closed |
| chr9.2 | 702,994,732 | 702,995,231 | not closed |
| chr10.1 | 63,746,282 | 63,746,781 | not closed |
| chr10.1 | 82,813,145 | 82,813,644 | not closed |
| chr10.1 | 144,835,204 | 144,835,703 | not closed |
| chr10.1 | 154,360,917 | 154,361,416 | not closed |
| chr10.1 | 159,340,809 | 159,341,308 | not closed |
| chr10.1 | 248,805,037 | 248,805,536 | not closed |
| chr10.1 | 248,969,605 | 248,970,104 | not closed |
| chr10.1 | 249,070,454 | 249,070,953 | not closed |
| chr10.1 | 333,055,583 | 333,056,082 | not closed |
| chr10.1 | 403,509,566 | 403,510,065 | not closed |
| chr10.1 | 623,295,734 | 623,296,233 | not closed |
| chr10.1 | 660,296,963 | 660,297,462 | not closed |
| chr10.1 | 702,836,035 | 702,836,534 | not closed |
| chr10.2 | 46,263,314 | 46,263,813 | not closed |
| chr10.2 | 66,333,234 | 66,333,733 | not closed |
| chr10.2 | 85,051,872 | 85,052,371 | not closed |
| chr10.2 | 147,903,105 | 147,903,604 | not closed |
| chr10.2 | 251,694,638 | 251,695,137 | not closed |
| chr10.2 | 330,177,182 | 330,177,681 | not closed |
| chr10.2 | 334,138,816 | 334,139,315 | not closed |
| chr10.2 | 372,658,184 | 372,658,683 | not closed |
| chr10.2 | 412,211,615 | 412,212,114 | not closed |
| chr10.2 | 544,403,642 | 544,404,141 | not closed |
| chr10.2 | 681,031,760 | 681,032,259 | not closed |
| chr11.1 | 183,701,263 | 183,701,762 | not closed |
| chr11.1 | 198,626,829 | 198,627,328 | not closed |
| chr11.1 | 433,790,293 | 433,790,792 | not closed |
| chr11.1 | 441,142,206 | 441,142,705 | not closed |
| chr11.1 | 465,703,215 | 465,703,714 | not closed |
| chr11.1 | 666,601,782 | 666,602,281 | not closed |
| chr11.2 | 53,433,599 | 53,434,098 | not closed |
| chr11.2 | 74,527,822 | 74,528,321 | not closed |
| chr11.2 | 92,496,727 | 92,497,226 | not closed |
| chr11.2 | 135,812,868 | 135,813,367 | not closed |
| chr11.2 | 167,015,659 | 167,016,158 | not closed |
| chr11.2 | 402,901,702 | 402,902,201 | not closed |
| chr11.2 | 436,127,716 | 436,128,215 | not closed |
| chr12.1 | 33,073,609 | 33,074,108 | not closed |
| chr12.1 | 253,011,505 | 253,012,004 | not closed |
| chr12.2 | 210,641,132 | 210,641,631 | not closed |
| chr12.2 | 237,222,008 | 237,222,507 | not closed |
| chr12.2 | 327,631,374 | 327,631,873 | not closed |
| chr1.1 | 64,727,929 | 64,728,428 | not closed |
| chr1.1 | 65,307,353 | 65,307,852 | not closed |
| chr1.1 | 419,108,043 | 419,108,542 | not closed |
| chr1.1 | 678,304,514 | 678,305,013 | not closed |
| chr1.1 | 800,661,811 | 800,662,310 | not closed |
| chr1.1 | 965,471,011 | 965,471,510 | not closed |
| chr1.1 | 1,024,206,216 | 1,024,206,715 | not closed |
| chr1.2 | 312,151,531 | 312,152,030 | not closed |
| chr1.2 | 348,147,843 | 348,148,342 | not closed |
| chr1.2 | 514,385,288 | 514,385,787 | not closed |
| chr1.2 | 599,926,837 | 599,927,336 | not closed |
| chr1.2 | 644,710,813 | 644,711,312 | not closed |
| chr1.2 | 656,605,361 | 656,605,860 | not closed |
| chr1.2 | 657,479,187 | 657,479,686 | not closed |
| chr1.2 | 659,537,629 | 659,538,128 | not closed |
| chr1.2 | 755,987,665 | 755,988,164 | not closed |
| chr1.2 | 854,710,770 | 854,711,269 | not closed |
| chr1.2 | 933,350,736 | 933,351,235 | not closed |
| chr1.2 | 957,011,533 | 957,012,032 | not closed |
| chr1.2 | 966,836,227 | 966,836,726 | not closed |
| chr2.2 | 104,857,404 | 104,857,903 | not closed |
| chr2.2 | 336,546,328 | 336,546,827 | not closed |
| chr2.2 | 659,991,920 | 659,992,419 | not closed |
| chr2.2 | 667,133,509 | 667,134,008 | not closed |
| chr2.2 | 667,557,751 | 667,558,250 | not closed |
| chr2.2 | 686,985,867 | 686,986,366 | not closed |
| chr2.2 | 722,138,030 | 722,138,529 | not closed |
| chr2.2 | 727,429,675 | 727,430,174 | not closed |
| chr2.2 | 754,245,154 | 754,245,653 | not closed |
| chr3.1 | 18,012,509 | 18,013,008 | not closed |
| chr3.1 | 92,414,208 | 92,414,707 | not closed |
| chr3.1 | 118,451,472 | 118,451,971 | not closed |
| chr3.1 | 330,849,151 | 330,849,650 | not closed |
| chr3.1 | 478,419,117 | 478,419,616 | not closed |
| chr3.1 | 621,588,873 | 621,589,372 | not closed |
| chr3.1 | 632,047,829 | 632,048,328 | not closed |
| chr3.1 | 901,110,728 | 901,111,227 | not closed |
| chr3.1 | 981,125,026 | 981,125,525 | not closed |
| chr3.2 | 128,475,907 | 128,476,406 | not closed |
| chr3.2 | 201,551,755 | 201,552,254 | not closed |
| chr3.2 | 228,427,847 | 228,428,346 | not closed |
| chr3.2 | 260,962,321 | 260,962,820 | not closed |
| chr3.2 | 352,909,412 | 352,909,911 | not closed |
| chr3.2 | 616,720,458 | 616,720,957 | not closed |
| chr3.2 | 743,494,077 | 743,494,576 | not closed |
| chr3.2 | 763,146,846 | 763,147,345 | not closed |
| chr3.2 | 970,019,926 | 970,020,425 | not closed |
| chr4.1 | 17,849,259 | 17,849,758 | not closed |
| chr4.1 | 325,667,183 | 325,667,682 | not closed |
| chr4.1 | 352,592,921 | 352,593,420 | not closed |
| chr4.1 | 364,072,276 | 364,072,775 | not closed |
| chr4.1 | 369,472,700 | 369,473,199 | not closed |
| chr4.1 | 373,005,914 | 373,006,413 | not closed |
| chr4.1 | 553,635,527 | 553,636,026 | not closed |
| chr4.1 | 554,555,582 | 554,556,081 | not closed |
| chr4.2 | 42,446,002 | 42,446,501 | not closed |
| chr4.2 | 48,037,637 | 48,038,136 | not closed |
| chr4.2 | 69,038,137 | 69,038,636 | not closed |
| chr4.2 | 78,332,584 | 78,333,083 | not closed |
| chr4.2 | 114,220,258 | 114,220,757 | not closed |
| chr4.2 | 477,152,908 | 477,153,407 | not closed |
| chr4.2 | 498,092,191 | 498,092,690 | not closed |
| chr4.2 | 509,509,974 | 509,510,473 | not closed |
| chr4.2 | 514,252,647 | 514,253,146 | not closed |
| chr4.2 | 652,046,445 | 652,046,944 | not closed |
| chr4.2 | 734,413,780 | 734,414,279 | not closed |
| chr4.2 | 796,217,095 | 796,217,594 | not closed |
| chr5.1 | 62,873 | 63,372 | not closed |
| chr5.1 | 19,423,792 | 19,424,291 | not closed |
| chr5.1 | 55,998,008 | 55,998,507 | not closed |
| chr5.1 | 281,281,029 | 281,281,528 | not closed |
| chr5.1 | 284,016,799 | 284,017,298 | not closed |
| chr5.1 | 545,419,870 | 545,420,369 | not closed |
| chr5.1 | 634,046,314 | 634,046,813 | not closed |
| chr5.1 | 641,142,888 | 641,143,387 | not closed |
| chr5.1 | 645,767,102 | 645,767,601 | not closed |
| chr5.1 | 646,713,914 | 646,714,413 | not closed |
| chr5.1 | 653,179,037 | 653,179,536 | not closed |
| chr5.1 | 674,580,237 | 674,580,736 | not closed |
| chr5.1 | 878,131,632 | 878,132,131 | not closed |
| chr5.2 | 34,741,641 | 34,742,140 | not closed |
| chr5.2 | 204,606,596 | 204,607,095 | not closed |
| chr5.2 | 230,070,367 | 230,070,866 | not closed |
| chr5.2 | 274,322,824 | 274,323,323 | not closed |
| chr5.2 | 372,099,020 | 372,099,519 | not closed |
| chr5.2 | 417,294,885 | 417,295,384 | not closed |
| chr5.2 | 491,172,741 | 491,173,240 | not closed |
| chr5.2 | 536,093,998 | 536,094,497 | not closed |

**Table S6.** Assembly quality of TWv1.

| **Chr id** | **QV (consensus quality value)** | **Error rate** |
| --- | --- | --- |
| chr1.1 | 62.7233 | 5.34162E-07 |
| chr2.1 | 62.1906 | 6.0386E-07 |
| chr3.1 | 62.237 | 5.97447E-07 |
| chr4.1 | 62.3359 | 5.8399E-07 |
| chr5.1 | 61.4839 | 7.10572E-07 |
| chr6.1 | 61.8567 | 6.52117E-07 |
| chr7.1 | 59.2436 | 1.19025E-06 |
| chr8.1 | 61.1676 | 7.64261E-07 |
| chr9.1 | 57.8842 | 1.62771E-06 |
| chr10.1 | 59.828 | 1.04039E-06 |
| chr11.1 | 58.4513 | 1.42847E-06 |
| chr12.1 | 58.5009 | 1.41224E-06 |
| chr1.2 | 61.1579 | 7.65968E-07 |
| chr2.2 | 62.7626 | 5.29344E-07 |
| chr3.2 | 62.6943 | 5.37732E-07 |
| chr4.2 | 64.0586 | 3.92768E-07 |
| chr5.2 | 62.5525 | 5.55587E-07 |
| chr6.2 | 58.1381 | 1.53529E-06 |
| chr7.2 | 57.3554 | 1.83849E-06 |
| chr8.2 | 58.1306 | 1.53794E-06 |
| chr9.2 | 62.8869 | 5.14407E-07 |
| chr10.2 | 63.7807 | 4.18722E-07 |
| chr11.2 | 57.2266 | 1.89384E-06 |
| chr12.2 | 63.699 | 4.26674E-07 |

**Table S7.** BUSCOs analysis of TWv1 genome completeness.

| **Description** | **TWv1-hap1** | | **TWv1-hap2** | | **TWv1** | |
| --- | --- | --- | --- | --- | --- | --- |
|  | **embryophyta_odb10** | **Gymnosperm_odb10** | **embryophyta_odb10** | **Gymnosperm_odb10** | **embryophyta_odb10** | **Gymnosperm_odb10** |
| Complete BUSCOs (C) | 89.1% | 95.80% | 90.3% | 97.10% | 92.50% | 97.69% |
| Complete and single-copy BUSCOs (S) | 82.30% | 92.20% | 83.50% | 93.20% | 4.40% | 2.31% |
| Complete and duplicated BUSCOs (D) | 6.80% | 3.60% | 6.80% | 3.90% | 88.10% | 95.38% |
| Fragmented BUSCOs (F) | 4.50% | 0.70% | 4.60% | 0.90% | 4.09% | 0.75% |
| Missing BUSCOs (M) | 6.40% | 3.50% | 5.10% | 2.00% | 3.41% | 1.56% |

**Table S8.** Survey results of telomeric sequence.

| **Chr id** | **Chr length(bp)** | **Status** | Left num* | **Left direction** | Rightnum* | **Right direction** |
| --- | --- | --- | --- | --- | --- | --- |
| chr1.1 | 1,082,079,411 | both | 3,042 | + | 5496 | - |
| chr1.2 | 1,070,229,389 | both | 24 | + | 5,537 | - |
| chr2.1 | 1,013,456,143 | both | 3,537 | + | 955 | - |
| chr2.2 | 1,004,722,113 | both | 1,791 | + | 1,624 | - |
| chr3.1 | 990,347,785 | both | 1,790 | + | 7,181 | - |
| chr3.2 | 978,819,918 | both | 6,135 | + | 6,652 | - |
| chr4.1 | 768,292,334 | both | 1,967 | + | 514 | - |
| chr4.2 | 865,144,497 | both | 34 | - | 1,976 | - |
| chr5.1 | 947,120,266 | both | 16 | - | 6,648 | - |
| chr5.2 | 950,554,962 | both | 22 | + | 5,880 | - |
| chr6.1 | 920,688,933 | both | 1,899 | + | 5,566 | - |
| chr6.2 | 916,822,420 | both | 1,523 | + | 1,336 | - |
| chr7.1 | 764,671,625 | both | 2,337 | + | 2,325 | - |
| chr7.2 | 793,725,128 | both | 1,726 | + | 4,369 | - |
| chr8.1 | 780,722,421 | both | 34 | + | 6,841 | - |
| chr8.2 | 800,879,169 | both | 2,508 | + | 2,869 | - |
| chr9.1 | 788,496,199 | both | 2,698 | + | 2,854 | - |
| chr9.2 | 783,637,138 | both | 849 | + | 9,225 | - |
| chr10.1 | 720,462,218 | both | 571 | - | 1,594 | - |
| chr10.2 | 714,373,249 | both | 403 | - | 2,033 | - |
| chr11.1 | 671,259,350 | both | 1,317 | + | 24 | - |
| chr11.2 | 673,220,661 | both | 2,759 | + | 2,985 | - |
| chr12.1 | 420,516,475 | both | 1,135 | + | 1,353 | - |
| chr12.2 | 427,687,534 | both | 971 | + | 470 | + |

num*: Number of telomeric repeat sequences.

**Table S9.** Gene annotation.

| **Type** | **TWv1-hap1** | **TWv1-hap2** |
| --- | --- | --- |
| gene numbers | 47,721 | 48,455 |
| the average of mRNA length (bp) | 26,750.58 | 26,122.50 |
| the average of cds_length (bp) | 1,118.82 | 1,110.76 |
| the average of exon number | 4.44 | 4.38 |
| the average of exon length (bp) | 252.27 | 253.34 |
| the average of intron length (bp) | 7,461.79 | 7,390.17 |

**Table S10.** Summary of transposable elements.

| **Type** | **TWv1-hap1** | | **TWv1-hap2** | |
| --- | --- | --- | --- | --- |
|  | **Repeat Size(bp)** | **% of genome** | **Repeat Size(bp)** | **% of genome** |
| DNA | 1,872,872,693 | 18.98 | 1,910,419,916 | 19.14 |
| LINE | 29,339,701 | 0.3 | 29,642,964 | 0.3 |
| LTR | 5,692,908,025 | 57.69 | 5,740,633,486 | 57.52 |
| Unknown | 695,289,302 | 7.05 | 704,889,549 | 7.06 |
| Total | 8,290,409,721 | 84.01 | 8,385,585,915 | 84.03 |

**Table S11.** Structural variation(SV) in the Twv1 genome.

| **Chr id** | **Start** | **Stop** | **Chr id** | **Start** | **Stop** | **SV** |
| --- | --- | --- | --- | --- | --- | --- |
| chr1.1 | 79036 | 2085658 | chr1.2 | 657569684 | 659480275 | TRANS |
| chr1.1 | 7536909 | 7676892 | chr1.2 | 4997874 | 5185821 | INV |
| chr1.1 | 31681716 | 31889375 | chr1.2 | 27288259 | 27532757 | INV |
| chr1.1 | 59233888 | 59476355 | chr1.2 | 5218372 | 5464041 | INVTR |
| chr1.1 | 106511521 | 107148730 | chr1.2 | 108879568 | 109541418 | INVDP |
| chr1.1 | 116775112 | 117438520 | chr1.2 | 111303122 | 111952406 | INVDP |
| chr1.1 | 116775112 | 120371313 | chr1.2 | 110107229 | 111140026 | INV |
| chr1.1 | 119845793 | 120180624 | chr1.2 | 109609795 | 109932811 | DUP |
| chr1.1 | 120614659 | 120731151 | chr1.2 | 107414392 | 107530532 | INVTR |
| chr1.1 | 133771744 | 134035607 | chr1.2 | 123684634 | 123922661 | INV |
| chr1.1 | 134509068 | 137100346 | chr1.2 | 124220661 | 126992773 | INV |
| chr1.1 | 146244090 | 146383409 | chr1.2 | 138273857 | 138411599 | DUP |
| chr1.1 | 169830548 | 170321558 | chr1.2 | 160473726 | 161008274 | INV |
| chr1.1 | 185914425 | 186328930 | chr1.2 | 173969991 | 174372904 | INV |
| chr1.1 | 191796639 | 192383420 | chr1.2 | 180308285 | 180816257 | INV |
| chr1.1 | 194926045 | 197143953 | chr1.2 | 182357672 | 184670635 | INV |
| chr1.1 | 213352450 | 213694078 | chr1.2 | 202959104 | 203290720 | TRANS |
| chr1.1 | 288981433 | 289086661 | chr1.2 | 279210564 | 279293167 | TRANS |
| chr1.1 | 302014072 | 302217103 | chr1.2 | 289862420 | 290053092 | INVTR |
| chr1.1 | 303658755 | 303799544 | chr1.2 | 294404813 | 294515395 | INV |
| chr1.1 | 402262955 | 447972238 | chr1.2 | 393371006 | 439093119 | INV |
| chr1.1 | 486438429 | 487540140 | chr1.2 | 482838154 | 483933279 | INVTR |
| chr1.1 | 609101714 | 609977797 | chr1.2 | 656605861 | 657479186 | TRANS |
| chr1.1 | 626603334 | 626854837 | chr1.2 | 616470985 | 616722502 | INV |
| chr1.1 | 627435413 | 628338128 | chr1.2 | 617359465 | 618166952 | INV |
| chr1.1 | 628838806 | 628988068 | chr1.2 | 618449270 | 618598531 | INV |
| chr1.1 | 644347943 | 644622151 | chr1.2 | 634048019 | 634598440 | CPG |
| chr1.1 | 773152792 | 773287465 | chr1.2 | 765505869 | 765615161 | INV |
| chr1.1 | 795614857 | 796000596 | chr1.2 | 788602108 | 790970153 | INV |
| chr1.1 | 816520575 | 816788726 | chr1.2 | 812048043 | 812352403 | INV |
| chr1.1 | 849768224 | 850298679 | chr1.2 | 843995906 | 844282178 | INV |
| chr1.1 | 850509044 | 851820188 | chr1.2 | 844365828 | 845696215 | INV |
| chr1.1 | 869016900 | 871313552 | chr1.2 | 862278347 | 864485366 | INV |
| chr1.1 | 879264586 | 879567185 | chr1.2 | 872698201 | 872974955 | INV |
| chr1.1 | 942982085 | 943109043 | chr1.2 | 933807488 | 933918952 | INV |
| chr1.1 | 966011676 | 966379313 | chr1.2 | 954781979 | 955169147 | INV |
| chr2.1 | 9923184 | 10093747 | chr2.2 | 10084741 | 10253873 | INV |
| chr2.1 | 19899726 | 20062936 | chr2.2 | 19593363 | 19593418 | CPL |
| chr2.1 | 50332704 | 50448384 | chr2.2 | 41225834 | 41324391 | INVTR |
| chr2.1 | 69046281 | 69152712 | chr2.2 | 58981777 | 59087371 | INV |
| chr2.1 | 81133890 | 81344838 | chr2.2 | 70761268 | 70994086 | INV |
| chr2.1 | 83959523 | 84253034 | chr2.2 | 73596058 | 73877698 | INV |
| chr2.1 | 160978520 | 161513577 | chr2.2 | 151784277 | 152338920 | INV |
| chr2.1 | 175988564 | 176524843 | chr2.2 | 164520561 | 165071852 | INV |
| chr2.1 | 216083444 | 217417971 | chr2.2 | 204670624 | 206289293 | TRANS |
| chr2.1 | 216373766 | 217119852 | chr2.2 | 207034009 | 207750744 | DUP |
| chr2.1 | 218345950 | 218949025 | chr2.2 | 208951934 | 209560883 | DUP |
| chr2.1 | 247783377 | 248035588 | chr2.2 | 239075827 | 239319217 | INV |
| chr2.1 | 254466583 | 254874572 | chr2.2 | 246064279 | 246472262 | DUP |
| chr2.1 | 351379058 | 357282397 | chr2.2 | 343894480 | 349795201 | INV |
| chr2.1 | 386347595 | 388062679 | chr2.2 | 378664496 | 380342236 | INV |
| chr2.1 | 402929969 | 403334600 | chr2.2 | 313099592 | 313504629 | DUP |
| chr2.1 | 465754859 | 466075296 | chr2.2 | 458186214 | 458507834 | INV |
| chr2.1 | 617356400 | 622426355 | chr2.2 | 609664967 | 614832305 | INV |
| chr2.1 | 672585762 | 672891800 | chr2.2 | 663970283 | 664288648 | INV |
| chr2.1 | 675824357 | 676248189 | chr2.2 | 667134009 | 667557750 | INV |
| chr2.1 | 733743475 | 733991131 | chr2.2 | 725738072 | 725969112 | TRANS |
| chr2.1 | 747337190 | 747522641 | chr2.2 | 740864782 | 741065110 | INVTR |
| chr2.1 | 750330085 | 750457294 | chr2.2 | 745550872 | 745645692 | TRANS |
| chr2.1 | 750520877 | 750782725 | chr2.2 | 745715390 | 745975672 | TRANS |
| chr2.1 | 752645517 | 753036407 | chr2.2 | 747911276 | 748310965 | INV |
| chr2.1 | 781893657 | 782012423 | chr2.2 | 775391915 | 775510491 | TRANS |
| chr2.1 | 790806668 | 791048408 | chr2.2 | 785202281 | 785521739 | INV |
| chr2.1 | 823492361 | 823629574 | chr2.2 | 820127342 | 820281477 | INV |
| chr2.1 | 824154574 | 824485630 | chr2.2 | 820582284 | 820861986 | INV |
| chr2.1 | 859988030 | 860486693 | chr2.2 | 855980447 | 856487866 | INV |
| chr2.1 | 861037907 | 861211455 | chr2.2 | 856272338 | 856428900 | INVDP |
| chr2.1 | 873191408 | 873359841 | chr2.2 | 867278683 | 867439061 | INV |
| chr2.1 | 882216541 | 882333477 | chr2.2 | 876286158 | 876400476 | INV |
| chr2.1 | 883625528 | 884268116 | chr2.2 | 877751396 | 878508284 | INV |
| chr2.1 | 908210583 | 908373032 | chr2.2 | 900398926 | 900670943 | INV |
| chr2.1 | 913083520 | 913229560 | chr2.2 | 905634242 | 905664295 | CPL |
| chr2.1 | 930626714 | 930755482 | chr2.2 | 923128112 | 923267760 | INV |
| chr2.1 | 1000068602 | 1000283562 | chr2.2 | 991966092 | 992161357 | INV |
| chr3.1 | 7739091 | 7983153 | chr3.2 | 9360865 | 9493164 | INV |
| chr3.1 | 68011725 | 69533180 | chr3.2 | 71361045 | 72948803 | INV |
| chr3.1 | 96744769 | 96946382 | chr3.2 | 100697436 | 100882583 | INV |
| chr3.1 | 124769492 | 124937767 | chr3.2 | 130693224 | 130902475 | INV |
| chr3.1 | 125711549 | 125898696 | chr3.2 | 131810856 | 132036871 | INV |
| chr3.1 | 179678699 | 179931125 | chr3.2 | 183697417 | 183777674 | INV |
| chr3.1 | 184502187 | 184703468 | chr3.2 | 188870987 | 189087970 | TRANS |
| chr3.1 | 185137641 | 185634146 | chr3.2 | 190050952 | 190313811 | INV |
| chr3.1 | 227265209 | 227463043 | chr3.2 | 228733771 | 228931338 | INV |
| chr3.1 | 236286526 | 236420706 | chr3.2 | 237859264 | 237960267 | TRANS |
| chr3.1 | 246648908 | 247681670 | chr3.2 | 246750991 | 246816115 | CPL |
| chr3.1 | 296556977 | 297332531 | chr3.2 | 294611087 | 295453951 | INV |
| chr3.1 | 343046908 | 343499394 | chr3.2 | 342206625 | 342630299 | INV |
| chr3.1 | 409770469 | 436321821 | chr3.2 | 408674573 | 434913825 | INV |
| chr3.1 | 428304917 | 429007805 | chr3.2 | 416367395 | 416022606 | CPL |
| chr3.1 | 438500054 | 438773109 | chr3.2 | 437065765 | 437614251 | CPG |
| chr3.1 | 450577246 | 450809902 | chr3.2 | 449015743 | 449231484 | INV |
| chr3.1 | 452647551 | 453380531 | chr3.2 | 451088691 | 451821413 | INV |
| chr3.1 | 576354104 | 577010054 | chr3.2 | 574300210 | 574951662 | DUP |
| chr3.1 | 577010090 | 577209406 | chr3.2 | 574077170 | 574279268 | DUP |
| chr3.1 | 590042631 | 590227272 | chr3.2 | 588782463 | 588782468 | CPL |
| chr3.1 | 593568759 | 593691585 | chr3.2 | 592345995 | 592467713 | INV |
| chr3.1 | 646120978 | 646959720 | chr3.2 | 644432676 | 644528682 | CPL |
| chr3.1 | 679176895 | 679444782 | chr3.2 | 677213875 | 677481575 | INV |
| chr3.1 | 685954823 | 686941645 | chr3.2 | 683976949 | 684968754 | INV |
| chr3.1 | 702267599 | 702446570 | chr3.2 | 700862798 | 701044267 | TRANS |
| chr3.1 | 702714075 | 702912955 | chr3.2 | 701395347 | 701574409 | TRANS |
| chr3.1 | 726928535 | 727085544 | chr3.2 | 723491424 | 723802755 | INV |
| chr3.1 | 736231187 | 736597638 | chr3.2 | 732780554 | 733264860 | INV |
| chr3.1 | 746660999 | 746874252 | chr3.2 | 741062778 | 741337999 | INV |
| chr3.1 | 749060606 | 749240677 | chr3.2 | 742912193 | 743093525 | INV |
| chr3.1 | 760365108 | 760547541 | chr3.2 | 753785254 | 753948415 | INVTR |
| chr3.1 | 784300372 | 784430048 | chr3.2 | 734535562 | 734682346 | INVTR |
| chr3.1 | 786059174 | 786357518 | chr3.2 | 780126468 | 780389596 | TRANS |
| chr3.1 | 786765757 | 787057710 | chr3.2 | 780849562 | 781081860 | INV |
| chr3.1 | 788285431 | 788495084 | chr3.2 | 782500382 | 782722338 | INV |
| chr3.1 | 788337895 | 788454668 | chr3.2 | 782397377 | 782500351 | DUP |
| chr3.1 | 798362683 | 801559305 | chr3.2 | 792281915 | 794180164 | INV |
| chr3.1 | 799118972 | 799297234 | chr3.2 | 791999867 | 792179785 | DUP |
| chr3.1 | 799592833 | 799761649 | chr3.2 | 795296352 | 795481013 | DUP |
| chr3.1 | 799650358 | 799832471 | chr3.2 | 795367899 | 795602366 | DUP |
| chr3.1 | 801699534 | 802387148 | chr3.2 | 794180501 | 794958656 | INV |
| chr3.1 | 832344095 | 832642793 | chr3.2 | 823438536 | 823773961 | INV |
| chr3.1 | 843362501 | 843640646 | chr3.2 | 835065350 | 835492018 | INV |
| chr3.1 | 847434751 | 847547269 | chr3.2 | 839307437 | 839440444 | DUP |
| chr3.1 | 914099287 | 914623660 | chr3.2 | 906121061 | 906527596 | INV |
| chr3.1 | 929375930 | 931949720 | chr3.2 | 919069228 | 920809507 | INV |
| chr3.1 | 954423280 | 954804756 | chr3.2 | 943498503 | 943752703 | INV |
| chr3.1 | 955560794 | 957479991 | chr3.2 | 944443582 | 945785926 | INV |
| chr3.1 | 987735073 | 988206082 | chr3.2 | 976111488 | 976735788 | INV |
| chr5.1 | 64849 | 8381725 | chr5.2 | 35296233 | 43732823 | INVTR |
| chr5.1 | 18867438 | 19407094 | chr5.2 | 34758266 | 35285270 | INVTR |
| chr5.1 | 25576969 | 25680389 | chr5.2 | 5401856 | 5517883 | TRANS |
| chr5.1 | 25604133 | 25727642 | chr5.2 | 5432285 | 5578484 | TRANS |
| chr5.1 | 27540129 | 27654751 | chr5.2 | 5798212 | 5914564 | DUP |
| chr5.1 | 86675671 | 87834182 | chr5.2 | 74793179 | 75954650 | INV |
| chr5.1 | 101615006 | 102748758 | chr5.2 | 89817576 | 90989525 | INV |
| chr5.1 | 111932725 | 113460405 | chr5.2 | 99964422 | 101585803 | INV |
| chr5.1 | 112590284 | 112767991 | chr5.2 | 101697661 | 101866232 | DUP |
| chr5.1 | 117699685 | 118037436 | chr5.2 | 108857246 | 109332237 | INV |
| chr5.1 | 155815427 | 157257113 | chr5.2 | 225125889 | 226470941 | TRANS |
| chr5.1 | 156149827 | 157538777 | chr5.2 | 225465927 | 226688202 | DUP |
| chr5.1 | 159942324 | 160537769 | chr5.2 | 227567031 | 228207339 | TRANS |
| chr5.1 | 160168956 | 160668127 | chr5.2 | 227778015 | 228268275 | DUP |
| chr5.1 | 160758914 | 161094151 | chr5.2 | 226989355 | 227339933 | TRANS |
| chr5.1 | 162328570 | 235586277 | chr5.2 | 147210647 | 218915648 | INV |
| chr5.1 | 235586276 | 239676346 | chr5.2 | 219239250 | 224843626 | INV |
| chr5.1 | 241996802 | 242114755 | chr5.2 | 108713270 | 108828438 | TRANS |
| chr5.1 | 242063954 | 242171927 | chr5.2 | 108021904 | 108117928 | TRANS |
| chr5.1 | 242110787 | 242233865 | chr5.2 | 108446396 | 108565486 | DUP |
| chr5.1 | 242147073 | 242271279 | chr5.2 | 108467879 | 108595235 | TRANS |
| chr5.1 | 268981447 | 269141923 | chr5.2 | 259735216 | 259871124 | TRANS |
| chr5.1 | 269049328 | 269167230 | chr5.2 | 258615849 | 258733620 | DUP |
| chr5.1 | 269066689 | 269206024 | chr5.2 | 259799096 | 259946548 | TRANS |
| chr5.1 | 269342692 | 269448049 | chr5.2 | 259236133 | 259336757 | DUP |
| chr5.1 | 269358671 | 269469649 | chr5.2 | 257516591 | 257622786 | DUP |
| chr5.1 | 269384314 | 269495182 | chr5.2 | 259273119 | 259383463 | DUP |
| chr5.1 | 269386641 | 269517597 | chr5.2 | 258187634 | 258328114 | DUP |
| chr5.1 | 269390679 | 269524565 | chr5.2 | 256460931 | 256602903 | DUP |
| chr5.1 | 274360855 | 274890148 | chr5.2 | 264425032 | 264952513 | INV |
| chr5.1 | 277823955 | 279547519 | chr5.2 | 267701718 | 269610662 | INV |
| chr5.1 | 281330369 | 281799931 | chr5.2 | 276550351 | 276901807 | INVTR |
| chr5.1 | 281725371 | 283948919 | chr5.2 | 272036715 | 274253873 | INV |
| chr5.1 | 284235125 | 284355806 | chr5.2 | 276429042 | 276528095 | TRANS |
| chr5.1 | 304896389 | 305083412 | chr5.2 | 296953867 | 297141930 | INV |
| chr5.1 | 305145887 | 305417344 | chr5.2 | 297204371 | 297207953 | CPL |
| chr5.1 | 305459235 | 305843589 | chr5.2 | 297772966 | 298135214 | TRANS |
| chr5.1 | 306007819 | 306232033 | chr5.2 | 303372619 | 303625097 | INVDP |
| chr5.1 | 306051361 | 306370777 | chr5.2 | 303182943 | 303573691 | INVTR |
| chr5.1 | 306157146 | 306426275 | chr5.2 | 303139112 | 303447349 | INVDP |
| chr5.1 | 317479698 | 318140677 | chr5.2 | 312121747 | 312809794 | INV |
| chr5.1 | 340972650 | 341139291 | chr5.2 | 333518864 | 333703985 | TRANS |
| chr5.1 | 355396221 | 355503835 | chr5.2 | 351760128 | 351859535 | TRANS |
| chr5.1 | 355436264 | 355545423 | chr5.2 | 351792027 | 351928655 | TRANS |
| chr5.1 | 364475924 | 364605047 | chr5.2 | 360703874 | 360837778 | INV |
| chr5.1 | 376364119 | 376599193 | chr5.2 | 372571068 | 372780127 | INV |
| chr5.1 | 477750886 | 491180175 | chr5.2 | 468461822 | 481847215 | INV |
| chr5.1 | 530961521 | 540040234 | chr5.2 | 521668797 | 530747378 | INV |
| chr5.1 | 605854961 | 606823615 | chr5.2 | 597017367 | 597986098 | INV |
| chr5.1 | 619036658 | 619266791 | chr5.2 | 610131145 | 610381848 | INV |
| chr5.1 | 646749368 | 653179036 | chr5.2 | 638818718 | 645568037 | INV |
| chr5.1 | 679627447 | 679728951 | chr5.2 | 675250401 | 675353488 | TRANS |
| chr5.1 | 681540906 | 686874411 | chr5.2 | 685973515 | 690520008 | TRANS |
| chr5.1 | 684311777 | 687412475 | chr5.2 | 688495107 | 691057088 | DUP |
| chr5.1 | 687888270 | 694835554 | chr5.2 | 678866313 | 685973506 | INV |
| chr5.1 | 701150855 | 701696777 | chr5.2 | 698384338 | 698918072 | DUP |
| chr5.1 | 701697227 | 702287786 | chr5.2 | 697803750 | 698390488 | DUP |
| chr5.1 | 702906316 | 703058609 | chr5.2 | 701765273 | 701897345 | DUP |
| chr5.1 | 702997282 | 703207159 | chr5.2 | 702482858 | 702647261 | DUP |
| chr5.1 | 703624297 | 705665299 | chr5.2 | 699810058 | 704872216 | INV |
| chr5.1 | 706637838 | 706800804 | chr5.2 | 705241014 | 705391011 | INV |
| chr5.1 | 726773326 | 726892476 | chr5.2 | 725199595 | 725325925 | INVTR |
| chr5.1 | 799518301 | 799994628 | chr5.2 | 801969141 | 802454962 | DUP |
| chr5.1 | 800310719 | 802025349 | chr5.2 | 800275534 | 801975951 | TRANS |
| chr5.1 | 803023875 | 803356498 | chr5.2 | 804642226 | 804947059 | INV |
| chr5.1 | 826931904 | 827041612 | chr5.2 | 827836271 | 827947985 | INV |
| chr5.1 | 833251131 | 833418904 | chr5.2 | 833497026 | 833603364 | INV |
| chr5.1 | 894689089 | 894973972 | chr5.2 | 898633765 | 898635580 | CPL |
| chr5.1 | 932284495 | 933043002 | chr5.2 | 935378702 | 936111197 | INV |
| chr6.1 | 7204417 | 7755251 | chr6.2 | 6328368 | 6889005 | INV |
| chr6.1 | 28341617 | 28507372 | chr6.2 | 26642800 | 26822000 | INV |
| chr6.1 | 31198942 | 31423865 | chr6.2 | 29521094 | 29638499 | INV |
| chr6.1 | 58626749 | 61558570 | chr6.2 | 56363799 | 58821894 | INV |
| chr6.1 | 142934425 | 143166917 | chr6.2 | 142181874 | 142427893 | DUP |
| chr6.1 | 143872248 | 143990486 | chr6.2 | 140503244 | 140582046 | TRANS |
| chr6.1 | 144143303 | 144286530 | chr6.2 | 140730430 | 140876668 | INVDP |
| chr6.1 | 177231431 | 178035737 | chr6.2 | 176310172 | 177114701 | INV |
| chr6.1 | 178169070 | 178797371 | chr6.2 | 177589658 | 178269348 | INV |
| chr6.1 | 206946605 | 207411534 | chr6.2 | 205831883 | 206266895 | INV |
| chr6.1 | 207600453 | 208320669 | chr6.2 | 206589978 | 207558385 | INV |
| chr6.1 | 211564237 | 211892942 | chr6.2 | 210940949 | 211280211 | INV |
| chr6.1 | 248196310 | 416915947 | chr6.2 | 247109135 | 414305434 | INV |
| chr6.1 | 451725394 | 454674768 | chr6.2 | 449235246 | 452201559 | INV |
| chr6.1 | 558114983 | 559305569 | chr6.2 | 556949562 | 558159837 | INV |
| chr6.1 | 561700061 | 569249913 | chr6.2 | 560315344 | 567884070 | INV |
| chr6.1 | 578674617 | 579583362 | chr6.2 | 577478430 | 578445697 | INV |
| chr6.1 | 587373387 | 588696526 | chr6.2 | 586164803 | 587481935 | INV |
| chr6.1 | 617166124 | 617641047 | chr6.2 | 615582205 | 616085981 | TRANS |
| chr6.1 | 659279637 | 660991214 | chr6.2 | 657510522 | 659074087 | INV |
| chr6.1 | 681747704 | 682154153 | chr6.2 | 680141395 | 680478977 | INV |
| chr6.1 | 698327561 | 699167604 | chr6.2 | 696083184 | 696895378 | INV |
| chr6.1 | 737466830 | 737581267 | chr6.2 | 737541505 | 737670072 | DUP |
| chr6.1 | 789313947 | 789453680 | chr6.2 | 786673281 | 786807472 | INV |
| chr6.1 | 826572794 | 826979445 | chr6.2 | 824879963 | 825341194 | INV |
| chr6.1 | 839184883 | 839667645 | chr6.2 | 837495000 | 838020084 | INV |
| chr6.1 | 867037754 | 867508016 | chr6.2 | 863601207 | 864091202 | INV |
| chr6.1 | 884881657 | 885001737 | chr6.2 | 881835530 | 881956782 | INV |
| chr6.1 | 895598993 | 896048292 | chr6.2 | 892587070 | 892872894 | INV |
| chr7.1 | 1932186 | 2108566 | chr7.2 | 2332090 | 2457414 | DUP |
| chr7.1 | 174281117 | 174468754 | chr7.2 | 173890697 | 174059475 | TRANS |
| chr7.1 | 183643823 | 183748023 | chr7.2 | 184231551 | 184320284 | TRANS |
| chr7.1 | 183676796 | 183796135 | chr7.2 | 184250280 | 184359301 | TRANS |
| chr7.1 | 193721985 | 196835143 | chr7.2 | 193772539 | 196329473 | INV |
| chr7.1 | 199926073 | 200047495 | chr7.2 | 200589817 | 200678726 | INV |
| chr7.1 | 217139150 | 217545586 | chr7.2 | 216832921 | 217296086 | INV |
| chr7.1 | 275141570 | 275863036 | chr7.2 | 273309598 | 274031671 | INV |
| chr7.1 | 426995195 | 427185264 | chr7.2 | 425003828 | 425195271 | DUP |
| chr7.1 | 426995195 | 427185264 | chr7.2 | 425226318 | 425417679 | DUP |
| chr7.1 | 491863016 | 493351744 | chr7.2 | 488485481 | 489973119 | INV |
| chr7.1 | 504339834 | 504465435 | chr7.2 | 500921110 | 501175583 | CPG |
| chr7.1 | 525998934 | 526314502 | chr7.2 | 522659863 | 522930370 | TRANS |
| chr7.1 | 529891493 | 547387587 | chr7.2 | 526099616 | 543649986 | INV |
| chr7.1 | 563251540 | 564037315 | chr7.2 | 559028708 | 559294675 | CPL |
| chr7.1 | 581468586 | 581605298 | chr7.2 | 583361588 | 583508108 | TRANS |
| chr7.1 | 581533353 | 583573438 | chr7.2 | 583435228 | 585441435 | TRANS |
| chr7.1 | 581536682 | 581873607 | chr7.2 | 581172630 | 581515734 | DUP |
| chr7.1 | 582303162 | 582824890 | chr7.2 | 579702981 | 580227198 | DUP |
| chr7.1 | 582586837 | 583513233 | chr7.2 | 579986303 | 580897218 | DUP |
| chr7.1 | 582825060 | 583778408 | chr7.2 | 580227302 | 581172082 | DUP |
| chr7.1 | 588340814 | 588518800 | chr7.2 | 590118804 | 590259961 | INV |
| chr7.1 | 619695549 | 619838749 | chr7.2 | 198455595 | 198606470 | INVTR |
| chr7.1 | 619772169 | 619941833 | chr7.2 | 198370330 | 198520566 | INVTR |
| chr7.1 | 620765952 | 621365449 | chr7.2 | 197456674 | 198094197 | INVTR |
| chr7.1 | 633072936 | 639412227 | chr7.2 | 652037645 | 652812789 | INV |
| chr7.1 | 691342106 | 691665534 | chr7.2 | 720088503 | 720347222 | INV |
| chr7.1 | 754094553 | 759266863 | chr7.2 | 782827688 | 788018106 | INV |
| chr9.1 | 1750218 | 1882582 | chr9.2 | 2081660 | 2200848 | INV |
| chr9.1 | 3673339 | 3905757 | chr9.2 | 4399049 | 4621093 | DUP |
| chr9.1 | 34510052 | 34636479 | chr9.2 | 34924659 | 35043407 | INV |
| chr9.1 | 45796493 | 46061235 | chr9.2 | 45892357 | 46285095 | INV |
| chr9.1 | 77014201 | 77147254 | chr9.2 | 76227013 | 76349461 | INV |
| chr9.1 | 107155606 | 107308675 | chr9.2 | 104879914 | 105045104 | INV |
| chr9.1 | 147398934 | 147759011 | chr9.2 | 144288630 | 144639884 | INV |
| chr9.1 | 162179646 | 162460554 | chr9.2 | 158663949 | 158701293 | CPL |
| chr9.1 | 174192537 | 174371855 | chr9.2 | 170511138 | 170679004 | DUP |
| chr9.1 | 174504397 | 174625357 | chr9.2 | 171018980 | 171153887 | INV |
| chr9.1 | 206472659 | 206691755 | chr9.2 | 202585749 | 202873191 | INV |
| chr9.1 | 213417096 | 213953426 | chr9.2 | 209325076 | 209808985 | INV |
| chr9.1 | 225337327 | 225711571 | chr9.2 | 221091575 | 221463683 | INV |
| chr9.1 | 229434217 | 229574548 | chr9.2 | 225219771 | 225354984 | INV |
| chr9.1 | 234319908 | 234431642 | chr9.2 | 230252286 | 230364068 | INV |
| chr9.1 | 335830980 | 336404219 | chr9.2 | 331133373 | 331425914 | CPL |
| chr9.1 | 341295555 | 489439614 | chr9.2 | 336277194 | 483054249 | INV |
| chr9.1 | 477593617 | 478537945 | chr9.2 | 347611007 | 347138874 | CPL |
| chr9.1 | 514085184 | 519081795 | chr9.2 | 507650855 | 512669330 | INV |
| chr9.1 | 532735696 | 532929066 | chr9.2 | 526269487 | 526463821 | INV |
| chr9.1 | 603582679 | 604229808 | chr9.2 | 597263305 | 598014806 | INV |
| chr9.1 | 666890476 | 667376789 | chr9.2 | 660486266 | 660707543 | INV |
| chr9.1 | 741098270 | 742019411 | chr9.2 | 735564389 | 736683225 | INV |
| chr9.1 | 758004404 | 758106562 | chr9.2 | 758806443 | 758881308 | DUP |
| chr9.1 | 760680545 | 762030641 | chr9.2 | 755608136 | 756986704 | DUP |
| chr9.1 | 761865058 | 762091624 | chr9.2 | 756798281 | 757045868 | DUP |
| chr9.1 | 763552356 | 763695288 | chr9.2 | 756824168 | 756990569 | DUP |
| chr9.1 | 767319757 | 767489514 | chr9.2 | 762606807 | 762758053 | INV |
| chr10.1 | 18841003 | 19050698 | chr10.2 | 18860277 | 18860320 | CPL |
| chr10.1 | 32627975 | 32764920 | chr10.2 | 34567862 | 34713361 | TRANS |
| chr10.1 | 76289927 | 76467817 | chr10.2 | 78555632 | 78708805 | INV |
| chr10.1 | 93775796 | 94233574 | chr10.2 | 95848468 | 95931399 | CPL |
| chr10.1 | 112369791 | 112645418 | chr10.2 | 115362315 | 115708060 | INV |
| chr10.1 | 126390113 | 138722092 | chr10.2 | 129212068 | 141526693 | INV |
| chr10.1 | 182188298 | 191995985 | chr10.2 | 184489949 | 193623852 | INV |
| chr10.1 | 227968916 | 237335636 | chr10.2 | 230722220 | 240102705 | INV |
| chr10.1 | 245475899 | 254107052 | chr10.2 | 248056759 | 254400562 | INV |
| chr10.1 | 248525615 | 249992695 | chr10.2 | 252130938 | 252044013 | CPL |
| chr10.1 | 255701729 | 256083309 | chr10.2 | 247649882 | 248016383 | TRANS |
| chr10.1 | 260192495 | 260337775 | chr10.2 | 256208111 | 256353104 | INV |
| chr10.1 | 282065390 | 283046743 | chr10.2 | 280090311 | 280998324 | INV |
| chr10.1 | 305202486 | 305353060 | chr10.2 | 301812606 | 301812733 | CPL |
| chr10.1 | 323383449 | 323630123 | chr10.2 | 319895502 | 320180526 | INV |
| chr10.1 | 333308874 | 333538133 | chr10.2 | 330915042 | 333060432 | INV |
| chr10.1 | 360646685 | 361581860 | chr10.2 | 364665818 | 365297869 | INV |
| chr10.1 | 364298666 | 364428732 | chr10.2 | 367047739 | 367184646 | TRANS |
| chr10.1 | 374459992 | 374659874 | chr10.2 | 380789532 | 380992002 | INV |
| chr10.1 | 405190001 | 405293826 | chr10.2 | 411226872 | 411337607 | DUP |
| chr10.1 | 405190001 | 405293826 | chr10.2 | 411917403 | 412019410 | DUP |
| chr10.1 | 407562423 | 407678027 | chr10.2 | 408634510 | 408731919 | TRANS |
| chr10.1 | 408361007 | 408487641 | chr10.2 | 410022792 | 410151752 | TRANS |
| chr10.1 | 411334994 | 411441406 | chr10.2 | 415249583 | 415355460 | INV |
| chr10.1 | 466730417 | 467768049 | chr10.2 | 465315495 | 466380247 | INV |
| chr10.1 | 470196202 | 470381898 | chr10.2 | 468701691 | 468864975 | INV |
| chr10.1 | 472680258 | 472840139 | chr10.2 | 473633527 | 473807724 | TRANS |
| chr10.1 | 483524585 | 488159799 | chr10.2 | 485275959 | 489971767 | INV |
| chr10.1 | 498951080 | 499300977 | chr10.2 | 501910553 | 502245620 | INV |
| chr10.1 | 500118993 | 500368537 | chr10.2 | 503043699 | 503321151 | TRANS |
| chr10.1 | 568093499 | 568226957 | chr10.2 | 574889035 | 575016409 | TRANS |
| chr10.1 | 568269741 | 568392275 | chr10.2 | 575170315 | 575260875 | TRANS |
| chr10.1 | 575266156 | 575677889 | chr10.2 | 584562041 | 584936368 | TRANS |
| chr10.1 | 575444775 | 575944400 | chr10.2 | 584699234 | 585151789 | TRANS |
| chr10.1 | 654153006 | 654598377 | chr10.2 | 655811144 | 656400412 | TRANS |
| chr10.1 | 664487315 | 664995358 | chr10.2 | 665324870 | 666002883 | INV |
| chr10.1 | 690308719 | 690457177 | chr10.2 | 689835915 | 690016691 | DUP |
| chr10.1 | 690343389 | 690522920 | chr10.2 | 689909710 | 690081965 | DUP |
| chr10.1 | 690551030 | 690668903 | chr10.2 | 690159722 | 690278969 | DUP |
| chr10.1 | 692243361 | 692381700 | chr10.2 | 689410484 | 689546855 | TRANS |
| chr11.1 | 16025876 | 16151172 | chr11.2 | 15858127 | 15983916 | INV |
| chr11.1 | 23181148 | 23488705 | chr11.2 | 23149683 | 23461175 | INV |
| chr11.1 | 34006420 | 34230681 | chr11.2 | 33418502 | 33635705 | DUP |
| chr11.1 | 69277123 | 69569852 | chr11.2 | 67529616 | 67773051 | INV |
| chr11.1 | 91408862 | 91629519 | chr11.2 | 89230762 | 89477875 | TRANS |
| chr11.1 | 91678794 | 91891492 | chr11.2 | 89537435 | 89740301 | DUP |
| chr11.1 | 91784682 | 91922693 | chr11.2 | 89643609 | 89777918 | DUP |
| chr11.1 | 105179345 | 106255375 | chr11.2 | 102316557 | 103816052 | INV |
| chr11.1 | 112087174 | 112272085 | chr11.2 | 111630930 | 111630975 | CPL |
| chr11.1 | 121369453 | 121652144 | chr11.2 | 120651679 | 120999186 | INV |
| chr11.1 | 215835990 | 216241136 | chr11.2 | 216330730 | 216735766 | INV |
| chr11.1 | 358557131 | 358672281 | chr11.2 | 359238649 | 359353959 | INV |
| chr11.1 | 360095379 | 362219021 | chr11.2 | 360772575 | 362871487 | INV |
| chr11.1 | 410442851 | 413266331 | chr11.2 | 412415607 | 415151601 | INV |
| chr11.1 | 413390503 | 413517906 | chr11.2 | 412123550 | 412251715 | TRANS |
| chr11.1 | 433790793 | 439931037 | chr11.2 | 434913135 | 441015428 | INV |
| chr11.1 | 520321554 | 520552989 | chr11.2 | 520560020 | 520780525 | INV |
| chr11.1 | 524068659 | 524172557 | chr11.2 | 524162629 | 524271847 | TRANS |
| chr11.1 | 531324624 | 560804571 | chr11.2 | 531616633 | 561174927 | INV |
| chr11.1 | 651190125 | 651311130 | chr11.2 | 654429110 | 654552347 | INV |
| chr11.1 | 666609701 | 671048494 | chr11.2 | 668663442 | 673196280 | INV |
| chr4.2 | 10267746 | 11479988 | chr8.1 | 9735978 | 10833636 | INV |
| chr4.2 | 28059613 | 28280643 | chr8.1 | 28548300 | 28754261 | INV |
| chr4.2 | 65264767 | 65503762 | chr8.1 | 62789188 | 63020306 | DUP |
| chr4.2 | 95322972 | 95456716 | chr8.1 | 96821750 | 96942697 | INV |
| chr4.2 | 109249918 | 109474731 | chr8.1 | 106997171 | 107201966 | INV |
| chr4.2 | 138525731 | 138734369 | chr8.1 | 135525904 | 135730336 | INV |
| chr4.2 | 169325685 | 169735694 | chr8.1 | 167631655 | 168042266 | DUP |
| chr4.2 | 169735918 | 170374336 | chr8.1 | 166994476 | 167631656 | DUP |
| chr4.2 | 172986586 | 173408151 | chr8.1 | 171299134 | 171715447 | INV |
| chr4.2 | 193649186 | 193758343 | chr8.1 | 192015409 | 192107986 | TRANS |
| chr4.2 | 194207049 | 194440509 | chr8.1 | 192594853 | 192828965 | TRANS |
| chr4.2 | 197205236 | 197606338 | chr8.1 | 195636541 | 196030610 | INV |
| chr4.2 | 209270530 | 209572087 | chr8.1 | 207852244 | 208145689 | TRANS |
| chr4.2 | 209396703 | 209733048 | chr8.1 | 207951696 | 208291727 | DUP |
| chr4.2 | 209449687 | 209882305 | chr8.1 | 208022404 | 208470830 | TRANS |
| chr4.2 | 209581692 | 209696480 | chr8.1 | 208687899 | 208772411 | DUP |
| chr4.2 | 209630784 | 209740720 | chr8.1 | 208717031 | 208834728 | DUP |
| chr4.2 | 214896299 | 214997479 | chr8.1 | 214496302 | 214597448 | INV |
| chr4.2 | 241753018 | 241854782 | chr8.1 | 242632843 | 242775735 | INV |
| chr4.2 | 294805420 | 296334592 | chr8.1 | 294244329 | 295821671 | INV |
| chr4.2 | 351138501 | 358528707 | chr8.1 | 336954119 | 398819277 | INV |
| chr4.2 | 360943836 | 398397401 | chr8.1 | 403963349 | 425071020 | INV |
| chr4.2 | 425475883 | 467933364 | chr8.1 | 438677755 | 469015718 | INV |
| chr4.2 | 474716224 | 483059315 | chr8.1 | 469948990 | 475190027 | INV |
| chr4.2 | 546281783 | 617428432 | chr8.1 | 545771104 | 662881768 | INV |
| chr4.2 | 666909428 | 754472379 | chr8.1 | 701727279 | 744957524 | INV |
| chr4.2 | 755428848 | 768078007 | chr8.1 | 750028818 | 751096313 | INV |
| chr4.2 | 797530348 | 805117533 | chr8.1 | 762527779 | 768911245 | INV |
| chr4.2 | 808744451 | 816037154 | chr8.1 | 772265554 | 772653861 | INV |
| chr4.2 | 842313669 | 854609225 | chr8.1 | 778332649 | 779693621 | INV |
| chr8.2 | 5728913 | 5838389 | chr4.1 | 5079605 | 5190685 | INVDP |
| chr8.2 | 32681808 | 33070545 | chr4.1 | 31973126 | 32367395 | INV |
| chr8.2 | 37949322 | 38116316 | chr4.1 | 43184481 | 43351501 | INV |
| chr8.2 | 54262435 | 54645789 | chr4.1 | 59089433 | 59467956 | DUP |
| chr8.2 | 65412477 | 65626999 | chr4.1 | 70570577 | 70893014 | INV |
| chr8.2 | 74504545 | 74708918 | chr4.1 | 79594869 | 79679908 | INV |
| chr8.2 | 98139848 | 98298847 | chr4.1 | 102530258 | 102712150 | INV |
| chr8.2 | 163388839 | 164034290 | chr4.1 | 167807115 | 168413419 | INV |
| chr8.2 | 177538075 | 264170331 | chr4.1 | 182127733 | 269590669 | INV |
| chr8.2 | 264970167 | 265109128 | chr4.1 | 270475333 | 270614271 | INV |
| chr8.2 | 265223498 | 265420347 | chr4.1 | 270728644 | 270925463 | INV |
| chr8.2 | 301999645 | 302707815 | chr4.1 | 306432236 | 307149403 | INV |
| chr8.2 | 302844899 | 303159543 | chr4.1 | 307288223 | 307595744 | INV |
| chr8.2 | 334161150 | 334384629 | chr4.1 | 339801489 | 340025172 | INV |
| chr8.2 | 349926695 | 361796596 | chr4.1 | 354585447 | 355289673 | INV |
| chr8.2 | 367485408 | 405397326 | chr4.1 | 366051437 | 393567986 | INV |
| chr8.2 | 469106819 | 472084761 | chr4.1 | 491195612 | 492416738 | INV |
| chr8.2 | 472288210 | 491342616 | chr4.1 | 492564966 | 493387734 | INV |
| chr8.2 | 506448382 | 513231149 | chr4.1 | 511560809 | 511994621 | INV |
| chr8.2 | 654790543 | 655056182 | chr4.1 | 626408522 | 629629082 | INV |
| chr8.2 | 678634015 | 773440986 | chr4.1 | 643158202 | 737914562 | INV |
| chr8.2 | 779393696 | 787969624 | chr4.1 | 759129009 | 759656980 | INV |
| chr8.2 | 792413491 | 797212072 | chr4.1 | 760359275 | 763081303 | INV |
| chr12.1 | 1757953 | 1880533 | chr12.2 | 20445194 | 20567909 | INVTR |
| chr12.1 | 2913028 | 4571696 | chr12.2 | 16659300 | 19449242 | INVDP |
| chr12.1 | 5717359 | 6667208 | chr12.2 | 10928267 | 14660803 | INV |
| chr12.1 | 5887073 | 6016808 | chr12.2 | 14511954 | 11588174 | CPG |
| chr12.1 | 6068961 | 6217922 | chr12.2 | 6731637 | 6881021 | DUP |
| chr12.1 | 6068961 | 6217922 | chr12.2 | 15338464 | 15487863 | INVDP |
| chr12.1 | 6109994 | 6291525 | chr12.2 | 10746412 | 10928266 | DUP |
| chr12.1 | 6117643 | 6217904 | chr12.2 | 7950304 | 8050502 | DUP |
| chr12.1 | 71374852 | 73019626 | chr12.2 | 84395853 | 85988347 | INV |
| chr12.1 | 73025145 | 73377054 | chr12.2 | 85997957 | 86372495 | INV |
| chr12.1 | 76987716 | 77140728 | chr12.2 | 88235801 | 88370947 | INVTR |
| chr12.1 | 92405668 | 92526770 | chr12.2 | 105738875 | 105866529 | TRANS |
| chr12.1 | 92792072 | 92944089 | chr12.2 | 104709467 | 104856407 | TRANS |
| chr12.1 | 92867536 | 93021709 | chr12.2 | 104774949 | 104960659 | TRANS |
| chr12.1 | 112920625 | 113096000 | chr12.2 | 125961466 | 125961540 | CPL |
| chr12.1 | 170590780 | 170836962 | chr12.2 | 184296670 | 184557245 | INV |
| chr12.1 | 177083641 | 177438061 | chr12.2 | 191196087 | 191510548 | INV |
| chr12.1 | 185097742 | 185236151 | chr12.2 | 199560609 | 199708347 | INV |
| chr12.1 | 206912320 | 207021084 | chr12.2 | 212761760 | 212869863 | TRANS |
| chr12.1 | 207276474 | 207554427 | chr12.2 | 213062110 | 213376855 | TRANS |
| chr12.1 | 207432931 | 207603508 | chr12.2 | 213243600 | 213426111 | TRANS |
| chr12.1 | 207491515 | 207627405 | chr12.2 | 210661212 | 210790329 | DUP |
| chr12.1 | 210130221 | 210328065 | chr12.2 | 215449612 | 215639584 | INV |
| chr12.1 | 220415967 | 220709834 | chr12.2 | 226331166 | 226599998 | INV |
| chr12.1 | 339718394 | 339959936 | chr12.2 | 345947271 | 346188839 | INV |
| chr12.1 | 365459985 | 370584958 | chr12.2 | 371688930 | 376839906 | INV |
| chr12.1 | 393367246 | 393764363 | chr12.2 | 400798118 | 401122920 | INV |
| chr12.1 | 414757402 | 414932236 | chr12.2 | 421407377 | 421572851 | TRANS |

**Table S12.** Structural variation(SV) statistics.

| **SV** | **Number** | **% of genome** |
| --- | --- | --- |
| DUP | 64 | 0.21 |
| INV | 267 | 12.89 |
| TRANS | 66 | 0.26 |
| **SV Total** | 420 | 13.4 |

**Table S13.** Large-scale chromosomal structural variation.

| **Chr id** | **Start** | **End** | **Length** | **Type** |
| --- | --- | --- | --- | --- |
| chr4 | 124,316 | 365,649,670 | 365,525,354 | translocation to chr8 |
| chr8 | 1 | 323,444,112 | 323,444,112 | translocation to chr4 |
| chr6 | 248,196,310 | 416,915,947 | 168,719,637 | inversion |
| chr9 | 341,295,555 | 489,439,614 | 148,144,059 | inversion |

**Table S14.** Collapse status of Twv1.

| **Chr id** | **Chr length(bp)** | **Collapsed length(bp)** | **Predicted length** | **Length difference between haplotypes** | **Percentage length difference between haplotypes** |
| --- | --- | --- | --- | --- | --- |
| chr1.1 | 1,082,079,411 | 0 | 1,082,079,411 | 11,850,022 | 1.10% |
| chr1.2 | 1,070,229,389 | 0 | 1,070,229,389 |  | 1.11% |
| chr2.1 | 1,013,456,143 | 0 | 1,013,456,143 | 8,734,030 | 0.86% |
| chr2.2 | 1,004,722,113 | 0 | 1,004,722,113 |  | 0.87% |
| chr3.1 | 990,347,785 | 0 | 990,347,785 | 11,527,867 | 1.16% |
| chr3.2 | 978,819,918 | 0 | 978,819,918 |  | 1.18% |
| chr4.1 | 768,292,334 | 21,300,000 | - | - | - |
| chr4.2 | 865,144,497 | 112,200,000 | - |  | - |
| chr5.1 | 947,120,266 | 4,800,000 | 947,120,266 | 8,234,696 | 0.87% |
| chr5.2 | 950,554,962 | 0 | 955,354,962 |  | 0.86% |
| chr6.1 | 920,688,933 | 0 | 920,688,933 | 3,866,513 | 0.42% |
| chr6.2 | 916,822,420 | 0 | 916,822,420 |  | 0.42% |
| chr7.1 | 764,671,625 | 15,600,000 | 820,071,625 | 10,746,497 | 1.31% |
| chr7.2 | 793,725,128 | 55,400,000 | 809,325,128 |  | 1.33% |
| chr8.1 | 780,722,421 | 0 | - | - | - |
| chr8.2 | 800,879,169 | 9,000,000 | - |  | - |
| chr9.1 | 788,496,199 | 0 | 788,496,199 | 4,859,061 | 0.62% |
| chr9.2 | 783,637,138 | 0 | 783,637,138 |  | 0.62% |
| chr10.1 | 720,462,218 | 0 | 720,462,218 | 6,088,969 | 0.85% |
| chr10.2 | 714,373,249 | 0 | 714,373,249 |  | 0.85% |
| chr11.1 | 671,259,350 | 0 | 671,259,350 | 1,961,311 | 0.29% |
| chr11.2 | 673,220,661 | 0 | 673,220,661 |  | 0.29% |
| chr12.1 | 420,516,475 | 0 | 420,516,475 | 7,171,059 | 1.71% |
| chr12.2 | 427,687,534 | 0 | 427,687,534 |  | 1.68% |

**Table S15.** Distribution and Quantity of Major ODDs in Twv1.

| **Chr id** | **Start** | **Stop** | **ODD Number** |
| --- | --- | --- | --- |
| chr1 | 110960494 | 112051722 | 7 |
| chr1 | 175413136 | 175761854 | 3 |
| chr10 | 631024261 | 632020444 | 4 |
| chr12 | 79840049 | 80855683 | 3 |
| chr2 | 36947402 | 37055572 | 3 |
| chr3 | 334607011 | 334646047 | 3 |
| chr3 | 760792200 | 762686403 | 4 |
| chr3 | 863454129 | 865155523 | 7 |
| chr3 | 867050751 | 868084684 | 3 |

**Table S16.** 11 Sequences Selected through Co-expression Network Analysis.

| **Gene Number** | **Sequence accession** |
| --- | --- |
| 1 | TW10H1G3395v3.1 |
| 2 | TW05H1G0749v3.1 |
| 3 | TW10H1G3399v3.1 |
| 6 | TW04H1G1863v3.1 |
| 7 | TW02H2G4231v3.1 |
| 9 | TW06H1G1485v3.1 |
| 10 | TW10H1G3397v3.1 |
| 13 | TW10H2G7215v3.1 |
| 14 | TW02H1G0952v3.1 |
| 15 | TW05H1G1961v3.1 |
| 16 | TW10H2G7211v3.1 |

**Table S17.** Statistical analysis of CYP450s in Twv1.

| **Chr id** | **Value** | **Group** |
| --- | --- | --- |
| chr1 | 87 | Other CYP450 |
| chr1 | 1 | CYP725A |
| chr2 | 31 | Other CYP450 |
| chr2 | 2 | CYP725A |
| chr3 | 32 | Other CYP450 |
| chr3 | 3 | CYP725A |
| chr3 | 1 | CYP725B |
| chr4 | 45 | Other CYP450 |
| chr5 | 54 | Other CYP450 |
| chr5 | 1 | CYP725A |
| chr6 | 88 | Other CYP450 |
| chr7 | 28 | Other CYP450 |
| chr7 | 2 | CYP725A |
| chr8 | 68 | Other CYP450 |
| chr8 | 1 | CYP725A |
| chr9 | 32 | Other CYP450 |
| chr9 | 54 | CYP725A |
| chr10 | 51 | Other CYP450 |
| chr11 | 50 | Other CYP450 |
| chr12 | 10 | Other CYP450 |
| chr1 | 87 | Other CYP450 |
| chr1 | 1 | CYP725A |

**Table S18.** Mapping of Characterized Enzymes in the Biosynthetic Pathway of Paclitaxel on the Himalayan yew Genome.

| **Tag** | **Sequence accession** | **Chr id** | **Start** | **Stop** |
| --- | --- | --- | --- | --- |
| GGPPS | TW08H1G0548v3.2 | chr8.1 | 91945396 | 91946577 |
|  | TW12H1G0065v3.1 | chr12.1 | 38910900 | 38912371 |
| TXS | TW09H1G0429v3.1 | chr9.1 | 74313429 | 74317414 |
| T10βOH | TW09H1G0193v3.1 | chr9.1 | 25157525 | 25159183 |
|  | TW09H1G0171v3.1 | chr9.1 | 25770648 | 25772352 |
| T5αOH | TW09H1G0318v3.1 | chr9.1 | 51486779 | 51488563 |
| T13αOH | TW09H1G0129v3.1 | chr9.1 | 19758417 | 19760441 |
|  | TW09H1G0145v3.1 | chr9.1 | 22164666 | 22166305 |
|  | TW09H1G0142v3.1 | chr9.1 | 22032680 | 22034367 |
| T2αOH | TW09H1G0191v3.1 | chr9.1 | 25656975 | 25658738 |
|  | TW09H1G0190v3.1 | chr9.1 | 25627063 | 25628721 |
| T7βOH | TW09H1G0183v3.1 | chr9.1 | 25369213 | 25371044 |
| TBT | TW11H1G1909Ev3.1 | chr11.1 | 500048842 | 500050895 |
| DBAT | TW09H1G0536v3.1 | chr9.1 | 96201554 | 96203098 |
| BAPT | TW01H1G3399v3.1 | chr1.1 | 870835097 | 870836553 |
| DBTNBT | TW10H1G3230v3.1 | chr10.1 | 591255353 | 591256919 |
| PAM | TW07H1G2799v3.1 | chr7.1 | 683280097 | 683282329 |
| PCL | TW11H1G0118v3.1 | chr11.1 | 17172070 | 17176423 |
| T2'αOH (TB506) | TW03H1G1044v3.1 | chr3.1 | 251463331 | 251465009 |
|  | TW03H1G1045v3.1 | chr3.1 | 251475287 | 251476969 |
| epoxidase | TW02H1G1128v3.1 | chr2.1 | 305166376 | 305167503 |
| T9αOH | TW09H1G0189v3.1 | chr9.1 | 25607257 | 25608946 |
| T1βOH | TW09H1G0194v3.2 | chr9.1 | 25867530 | 25869199 |
|  | TW09H1G0195v3.1 | chr9.1 | 25879783 | 25881452 |
| TOT1 | TW09H1G0449v3.1 | chr9.1 | 76893971 | 76895644 |
|  | TW09H1G0450v3.1 | chr9.1 | 77013095 | 77014812 |
|  | TW09H1G0451v3.2 | chr9.1 | 77146248 | 77147963 |

**3. Reference for the Supplementary Information**

1. Mosca E, Cruz F, Gómez-Garrido J, et al. A Reference Genome Sequence for the European Silver Fir (Abies alba Mill.): A Community-Generated Genomic Resource. G3 2019;9(7):2039-49. https://doi.org/10.1534/g3.119.400083.

2. Guan R, Zhao Y, Zhang H, et al. Draft genome of the living fossil *Ginkgo biloba*. GigaScience 2016;5(1). https://doi.org/10.1186/s13742-016-0154-1.

3. Liu H, Wang X, Wang G, et al. The nearly complete genome of *Ginkgo biloba* illuminates gymnosperm evolution. Nat Plants 2021;7(6):748-56. https://doi.org/10.1038/s41477-021-00933-x.

4. Wan T, Liu Z-M, Li L-F, et al. A genome for gnetophytes and early evolution of seed plants. Nat Plants 2018;4(2):82-9. https://doi.org/10.1038/s41477-017-0097-2.

5. Sun C, Xie YH, Li Z, et al. The Larix kaempferi genome reveals new insights into wood properties. J Integr Plant Biol 2022;64(7):1364-73. https://doi.org/10.1111/jipb.13265.

6. Fu F, Song C, Wen C, et al. The Metasequoia genome and evolutionary relationships among redwoods. Plant Communications 2023;4(6). https://doi.org/10.1016/j.xplc.2023.100643.

7. Nystedt B, Street NR, Wetterbom A, et al. The Norway spruce genome sequence and conifer genome evolution. Nature 2013;497(7451):579-84. https://doi.org/10.1038/nature12211.

8. Gagalova KK, Warren RL, Coombe L, et al. Spruce giga‐genomes: structurally similar yet distinctive with differentially expanding gene families and rapidly evolving genes. The Plant Journal 2022;111(5):1469-85. https://doi.org/10.1111/tpj.15889.

9. Warren RL, Keeling CI, Yuen MMS, et al. Improved white spruce (*Picea glauca*) genome assemblies and annotation of large gene families of conifer terpenoid and phenolic defense metabolism. The Plant Journal 2015;83(2):189-212. https://doi.org/10.1111/tpj.12886.

10. Lo T, Coombe L, Gagalova KK, et al. Assembly and annotation of the black spruce genome provide insights on spruce phylogeny and evolution of stress response. G3 2024;14(1):jkad247. https://doi.org/10.1093/g3journal/jkad247.

11. Stevens KA, Wegrzyn JL, Zimin A, et al. Sequence of the sugar pine megagenome. Genetics 2016;204(4):1613-26. https://doi.org/10.1534/genetics.116.193227.

12. Niu S, Li J, Bo W, et al. The *Chinese pine* genome and methylome unveil key features of conifer evolution. Cell 2022;185(1):204-17. e14. https://doi.org/10.1016/j.cell.2021.12.006.

13. Neale DB, Wegrzyn JL, Stevens KA, et al. Decoding the massive genome of loblolly pine using haploid DNA and novel assembly strategies. Genome Biol 2014;151-13. https://doi.org/10.1186/gb-2014-15-3-r59.

14. Neale DB, McGuire PE, Wheeler NC, et al. The Douglas-fir genome sequence reveals specialization of the photosynthetic apparatus in *Pinaceae*. G3 2017;7(9):3157-67. https://doi.org/10.1534/g3.117.300078.

15. Neale DB, Zimin AV, Zaman S, et al. Assembled and annotated 26.5 Gbp coast redwood genome: a resource for estimating evolutionary adaptive potential and investigating hexaploid origin. G3 2022;12(1):jkab380. https://doi.org/10.1093/g3journal/jkab380.

16. Xiong X, Gou J, Liao Q, et al. The Taxus genome provides insights into paclitaxel biosynthesis. Nat Plants 2021;7(8):1026-36. https://doi.org/10.1038/s41477-021-00963-5.

17. Shalev TJ, El-Dien OG, Yuen MM, et al. The western redcedar genome reveals low genetic diversity in a self-compatible conifer. Genome Res 2022;32(10):1952-64. https://doi.org/10.1101/gr.276358.121.

18. Lou H, Song L, Li X, et al. The *Torreya grandis* genome illuminates the origin and evolution of gymnosperm-specific sciadonic acid biosynthesis. Nat Commun 2023;14(1):1315. https://doi.org/10.1038/s41467-023-37038-2.

19. Cheng J, Wang X, Liu X, et al. Chromosome-level genome of Himalayan yew provides insights into the origin and evolution of the paclitaxel biosynthetic pathway. Molecular Plant 2021;14(7):1199-209. https://doi.org/10.1016/j.molp.2021.04.015.

20. Song C, Fu F, Yang L, et al. *Taxus yunnanensis* genome offers insights into gymnosperm phylogeny and taxol production. Commun Biol 2021;4(1):1203. https://doi.org/10.1038/s42003-021-02697-8.

21. Han Y, Zhang W, Zhou B, et al. Chromosome‐level genome assembly of *Welwitschia mirabilis*, a unique Namib Desert species. Mol Ecol Resour 2022;22(1):391-403. https://doi.org/10.1111/1755-0998.13475.

22. Wan T, Liu Z, Leitch IJ, et al. The *Welwitschia* genome reveals a unique biology underpinning extreme longevity in deserts. Nature communications 2021;12(1):4247. https://doi.org/10.1038/s41467-021-24528-4.
