# Supplementary material for: Phased high-quality genome of the gymnosperm Himalayan Yew assists in paclitaxel pathway exploration
Source: Gigascience. 2025 Apr 4;14:giaf026. doi: 10.1093/gigascience/giaf026 (PMC11970372; doi:10.1093/gigascience/giaf026)
Supplement: giaf026_GIGA-D-24-00293_Revision_2 [file giaf026_giga-d-24-00293_revision_2.pdf]

GigaScience

Phased High-Quality Genome of the Gymnosperm Himalayan Yew Assists in Paclitaxel Pathway Exploration

--Manuscript Draft--

Manuscript Number:	GIGA-D-24-00293R2
Full Title:	Phased High-Quality Genome of the Gymnosperm Himalayan Yew Assists in Paclitaxel Pathway Exploration
Article Type:	Research
Funding Information:	The New Cornerstone Science Foundation — Prof. Wen Wang<br>the National Natural Science Foundation of China (No. 32371499) — Dr. Xiaonan Liu<br>the High-Performance Computing Platform of Jiaxing Synbiolab Biotechnology Co., Ltd. — Dr. Xiao Wang
Abstract:	Abstract<br>Background: Taxus wallichiana is an important species for paclitaxel production. Previous genome versions for Taxus spp. have been limited by extensive gaps, hindering the complete annotation and mining of paclitaxel (known as taxol commercially) synthesis pathway-related genes.<br>Results: Here, we present the first phased high-quality reference genome of Taxus wallichiana, which significantly improves assembly quality and corrects large-scale assembly errors present in previous versions. The two haplotypes are 9.87 Gb and 9.98 Gb in length, respectively, and all 24 chromosomes were assembled with telomeres at both ends. Based on this high-quality genome (TWv1), we inferred that the candidate sex chromosome of Taxus wallichiana is chr12, and its sex determination system may follow a ZW model. Particularly, we identified and experimentally validated a batch of 2-oxoglutarate/Fe(II)-dependent dioxygenases (ODDs), which may be key C4β-C20 epoxidases in the paclitaxel synthesis pathway.<br>Conclusions: This study not only provides a valuable data resource for gene mining in the biosynthetic pathways of secondary metabolites such as paclitaxel but also offers the highest-quality reference genome of gymnosperms to date for the identification of sex chromosomes, facilitating comparative genomic studies among gymnosperms.<br><br>Keywords: Gymnosperm, Taxus wallichiana, Phased high-quality genome, Paclitaxel, 2-oxoglutarate/Fe(II)-dependent dioxygenase (ODD)
Corresponding Author:	Wen Wang, Ph.D<br><br>CHINA
Corresponding Author Secondary Information:	
Corresponding Author's Institution:	
Corresponding Author's Secondary Institution:	
First Author:	Zhenzhu Li
First Author Secondary Information:	
Order of Authors:	Zhenzhu Li<br>Hang Zong<br>Xiaonan Liu<br>Xiao Wang<br>Shimeng Liu

Powered by Editorial Manager® and ProduXion Manager® from Aries Systems Corporation

	Xi Jiao
	Xianqing Chen
	Hao Wu
	Zhuoya Liu
	Zhongkai Wang
	Yongqiang Wang
	Yi Liu
	Botong Zhou
	Zihe Li
	Qiuhui Du
	Jing Li
	Jian Cheng
	Jie Bai
	Xiaoxi Zhu
	Yue Yang
	Guichun Liu
	Li Zhang
	Huifeng Jiang
	Wen Wang, Ph.D
**Order of Authors Secondary Information:**	
**Response to Reviewers:**	Dear Hongling,<br><br>Thank you very much for handling our manuscript entitled "Phased High-Quality Genome of the Gymnosperm Himalayan Yew Assists in Paclitaxel Pathway Exploration" (Manuscript_GIGA-D-24-00293R1) and accepting it in principle to publish in GigaScience as a research article. We thank the reviewers and you for providing valuable suggestions and comments.<br><br>The constructive suggestions and comments have helped us to improve the manuscript. Particularly, we follow your suggestions to improve the documentation of all software used, including providing references, identifiers, links, and RRIDs. Additionally, we have cited the DOI for all computational workflows utilized at the relevant locations in the manuscript. In addition, based on the suggestions from Reviewer 2, we have updated the content in Table 1 and revised the corresponding descriptions in the manuscript.<br>Thank you again for handling our manuscript. If further information is needed, please feel free to let me know.<br>Yours sincerely,<br><br>Wen<br>-------------------------<br>Wen Wang PhD.<br>School of Ecology and Environment, Northwestern Polytechnical University, Xi’an 710072, China;<br>Phone: +86 18629418666;<br>Email: wenwang@nwpu.edu.cn<br><br><br>Response to Reviewer<br><br>We would like to thank the reviewers for the helpful comments, all of which have helped to improve this manuscript. Please find our point-by-point responses to the

	comments as follows.<br><br>Reviewer #1:<br>Thank you very much for your review and positive feedback on our work.<br><br>Reviewer #2<br>Thank you very much for your review and comments. We have modified the manuscript according to your suggestions, and our responses are as follows:<br><br>1. In Table 1, the number for the genome size for TWv1-hap2 is missing comma separators. Also, "Error rate" and "QV" are the same thing, just represented differently. I would recommend getting rid of the "Error rate" line or replacing it with "Errors per 1Mbp of sequence", as it may be easier to parse for the readers.<br><br>Response:<br>Thank you very much for your suggestions. Following your suggestions, we have added comma separators to the relevant genome size data in Table 1. Additionally, we have removed the "Error rate" row from Table 1 and described the Errors per Mb at Line 286-287: "The overall completeness is 99.36%, which corresponds to approximately one base error per 1 Mbp (Table 1).".<br><br>2. I also would like to see the numbers listed for completeness evaluations by Merqury for the two haplotypes (TWv1-hap1 and TWv1-hap2) in Table 1.<br><br>Response:<br>Thank you for noting this point. The completeness evaluated by Merqury may not be applicable for haplotype genomes. because the metric is calculated as: (Number of K-mer types in the genome) / (Number of K-mer types in the NGS reads). Therefore, when calculated for the entire genome (TWv1-hap1 + TWv1-hap2), the metric reflects the overall genome quality. However, for haplotype genomes, the denominator remains the "number of K-mer types in the NGS reads," which cannot barely be distinguished between haplotypes. As the differences between haplotypes increase, the K-mer types in a single haplotype genome become less representative of the entire genome, resulting in a lower completeness value for the haplotypes. Therefore, the completeness values evaluated by Merqury are not shown in Table 1.
**Additional Information:**	
**Question**	**Response**
Are you submitting this manuscript to a special series or article collection?	No
**Experimental design and statistics**<br><br>Full details of the experimental design and statistical methods used should be given in the Methods section, as detailed in our Minimum Standards Reporting Checklist. Information essential to interpreting the data presented should be made available in the figure legends.<br><br>Have you included all the information requested in your manuscript?	Yes
**Resources**	Yes

A description of all resources used, including antibodies, cell lines, animals and software tools, with enough information to allow them to be uniquely identified, should be included in the Methods section. Authors are strongly encouraged to cite Research Resource Identifiers (RRIDs) for antibodies, model organisms and tools, where possible.<br><br>Have you included the information requested as detailed in our Minimum Standards Reporting Checklist?	
**Availability of data and materials**<br><br>All datasets and code on which the conclusions of the paper rely must be either included in your submission or deposited in publicly available repositories (where available and ethically appropriate), referencing such data using a unique identifier in the references and in the “Availability of Data and Materials” section of your manuscript.<br><br>Have you have met the above requirement as detailed in our Minimum Standards Reporting Checklist?	Yes

Powered by Editorial Manager® and ProduXion Manager® from Aries Systems Corporation

Manuscript

Click here to access/download;Manuscript;Manuscript_20250226.docx

Phased High-Quality Genome of the Gymnosperm Himalayan Yew Assists in Paclitaxel Pathway Exploration

Zhenzhu Li[1, †], Hang Zong[1, †], Xiaonan Liu[2, 8, †], Xiao Wang[2, 3], Shimeng Liu[3], Xi Jiao[3], Xianqing Chen[3], Hao Wu[3], Zhuoya Liu[1], Zhongkai Wang[1], Yongqiang Wang[7], Yi Liu[1], Botong Zhou[1], Zihe Li[1], Qiuhui Du[3], Jing Li[2], Jian Cheng[2], Jie Bai[2], Xiaoxi Zhu[2], Yue Yang[4], Guichun Liu[5], Li Zhang[6], Huifeng Jiang[2, *] and Wen Wang[1, *]

[1]New Cornerstone Science Laboratory, Shaanxi Key Laboratory of Qinling Ecological Intelligent Monitoring and Protection, School of Ecology and Environment, Northwestern Polytechnical University, Xi'an, Shaanxi 710072, China
[2]Key Laboratory of Systems Microbial Biotechnology, Tianjin Institute of Industrial Biotechnology, Chinese Academy of Sciences, Tianjin 300308, China
[3]Jiaxing Synbiolab Biotechnology Co., Ltd., Jiaxing 314006, China
[4]College of Traditional Chinese Medicine of Jinggangshan University, Ji'an 343009, China
[5]State Key Laboratory of Genetic Resources and Evolution, Kunming Institute of Zoology, Chinese Academy of Sciences, Kunming 650201, China
[6]Chinese Institute for Brain Research (CIBR), Beijing 102206, China
[7]Sanjie Institute of Forage, Yangling, 712100, China
[8]Cooperative Innovation Center of Industrial Fermentation (Ministry of Education & Hubei Province), Key Laboratory of Fermentation Engineering (Ministry of Education), Hubei Key Laboratory of Industrial Microbiology, National "111" Center for Cellular Regulation and Molecular Pharmaceutics, Hubei University of Technology, Wuhan 430068, China
*Correspondence address. Wen Wang, E-mail: wenwang@nwpu.edu.cn; Huifeng Jiang, E-mail: jiang_hf@tib.cas.cn
†These authors contributed equally to this article.

ORCID iDs:

Zhenzhu Li [0009-0000-2060-5822]; Hang Zong [0000-0002-9690-8800]; Xiaonan Liu [0000-0003-2708-0971]; Xiao Wang [0000-0003-1955-3687]; Shimeng Liu [0009-0000-8722-6472]; Xi Jiao [0009-0009-8380-1631]; Xianqing Chen [0000-0001-7889-7290]; Hao Wu [0009-0003-6762-7203]; Zhuoya Liu [0009-0003-5321-3595]; Zhongkai Wang [0000-0003-0578-5735]; Yongqiang Wang [0009-0008-4897-0926]; Yi Liu [0009-0000-5166-9474]; Botong Zhou [0000-0002-1091-5867]; Zihe Li [0000-0003-4085-6850]; Qiuhui Du [0009-0000-1005-748X]; Jing Li [0009-0001-4550-8751]; Jian Cheng

[0000-0002-1709-7676]; Jie Bai [0000-0002-5051-7169]; Xiaoxi Zhu [0009-0007-1784-6757]; Yue Yang [0009-0006-4011-9657]; Guichun Liu [0000-0002-0899-7808]; Li Zhang [0000-0002-0698-0754]; Huifeng Jiang [0000-0002-3757-7896]; Wen Wang [0000-0002-7801-2066];

Abstract

Background: *Taxus wallichiana* is an important species for paclitaxel production. Previous genome versions for *Taxus spp.* have been limited by extensive gaps, hindering the complete annotation and mining of paclitaxel (known as taxol commercially) synthesis pathway-related genes.

Results: Here, we present the first phased high-quality reference genome of *Taxus wallichiana*, which significantly improves assembly quality and corrects large-scale assembly errors present in previous versions. The two haplotypes are 9.87 Gb and 9.98 Gb in length, respectively, and all 24 chromosomes were assembled with telomeres at both ends. Based on this high-quality genome (TWv1), we inferred that the candidate sex chromosome of *Taxus wallichiana* is chr12, and its sex determination system may follow a ZW model. Particularly, we identified and experimentally validated a batch of 2-oxoglutarate/Fe(II)-dependent dioxygenases (ODDs), which may be key C4β-C20 epoxidases in the paclitaxel synthesis pathway.

Conclusions: This study not only provides a valuable data resource for gene mining in the biosynthetic pathways of secondary metabolites such as paclitaxel but also offers the highest-quality reference genome of gymnosperms to date for the identification of sex chromosomes, facilitating comparative genomic studies among gymnosperms.

Keywords: Gymnosperm, *Taxus wallichiana*, Phased high-quality genome, Paclitaxel, 2-oxoglutarate/Fe(II)-dependent dioxygenase (ODD)

Introduction

The Himalayan yew (*Taxus wallichiana*, NCBI:txid147273) is a gymnosperm species

endemic to the regions east of the Himalayas in China. It has long been utilized as a medicinal plant due to its higher paclitaxel (known as taxol commercially) content compared to other yew species [1]. Gymnosperm genomes are notoriously large and complex, characterized by numerous gene families, repetitive sequences, transposons, genome rearrangements, as well as abundant unique non-coding sequences, all contributing to genome expansion and complexity [2, 3]. Previously, three haploid genomes of the genus *Taxus* have been published [4-6], including *Taxus wallichiana* (*T. wallichiana*) from our group [4]. However, these genomes were assembled using either second-generation sequencing or third-generation sequencing technologies with relatively high error rates, resulting in a large number of unfilled gaps (11,130, 8,004, and 12,092 gaps, respectively) and many assembly errors. These assembly issues and unphased assembly pose challenges for the identification of key metabolite biosynthetic enzymes and sex chromosomes.

Gymnosperms typically exhibit dioecy, with sex determination in dioecious taxa relying on heteromorphic chromosomes in male and female plants [7]. However, in most gymnosperms, sex differentiation is at an early stage of formation and is driven by epigenetic control, such as differences in cytosine methylation between the sexes [8]. Dioecy has independently arisen multiple times during the evolution of gymnosperms [9], leading to the development of various sex determination systems. Currently it is still difficult to uncover and explain the exact mechanisms behind the origin of these systems. For instance, the sex chromosomes of the three previously published *Taxus* genomes have yet to be identified. Obtaining a high-quality genome of the Himalayan yew will facilitate more in-depth studies on the sex differentiation of gymnosperms.

Paclitaxel is a renowned drug for treating breast cancer, ovarian cancer, and lung cancer [10-13]. However, the content of paclitaxel in yew is extremely low even in the Himalayan yew (about 0.001%) [14]. In addition, the long growth cycle and scarce resources of yew trees have severely limited the availability of paclitaxel on a large scale from the natural sources. On the other hand, the synthesis pathway of

paclitaxel is assumed to be very complex [15] and comprehensively excavation of synthesis enzymes has been hindered by incomplete yew genome assemblies. Therefore, to obtain a high-quality yew genome and elucidate the paclitaxel synthesis pathway in yew has been a critical step in identifying enzymes for the paclitaxel synthesis through synthetic biology.

For a long period, only 14 enzymatic reactions in the paclitaxel biosynthetic pathway were resolved [16]. Notably, a new epoxidase, 2-oxoglutarate/Fe(II)-dependent dioxygenase (ODD), has been discovered [17]. It is hypothesized to catalyze the first step of taxadiene oxidation, specifically responsible for the C4β-C20 epoxidation. However, due to the inability to isolate any epoxidase products in large quantities for nuclear magnetic resonance structural identification, the catalytic process of this epoxidase remains speculative [17]. In plants, ODDs are non-heme iron proteins that are soluble and localized in the cytoplasm. ODD enzymes are involved in various biological processes, including the biosynthesis of specialized metabolites such as plant hormones and flavonoids [18]. Oxygenation/hydroxylation reactions catalyzed by dioxygenases are particularly important in paclitaxel biosynthesis research. Recently, Zhao et al. discovered that the single enzyme CYP725A4 with C5 hydroxylation function can catalyze two consecutive epoxidation events, leading to the formation of an oxetane ring [19]. Jiang et al. identified a bifunctional cytochrome P450 enzyme TOT1, which can directly convert the olefin part into an epoxide and an oxetane ring, respectively, but this enzyme cannot function as an isomerase to convert the epoxide ring into the oxetane ring [20]. These pieces of evidence suggest that ODDs and CYP450s play key roles in the upstream biosynthesis steps of paclitaxel and indicate the presence of different epoxidases in yews, which may imply the existence of multiple catalytic synthesis pathways for paclitaxel precursors, hence exhibiting substrate promiscuity.

In this study, we obtained the first phased high-quality genome of a gymnosperm species, the Himalayan yew, and assembled the longest T2T chromosome to date. This is not only the highest-quality genome reported for yew so far but also the

highest-quality genome assembled across the entire gymnosperm phylum. Through comprehensive annotation and analysis of this genome, we inferred candidate sex chromosomes in the Himalayan yew and identified a set of crucial ODD enzymes, which exhibit C4β-C20 epoxidase activity as validated by our intensive experiments. The high-quality assembly and in-depth analysis of the *T. wallichiana* genome presented in this study provide valuable resources for identifying key enzymes involved in paclitaxel biosynthesis and offer important references for understanding the complex genomes of gymnosperms.

Materials and Methods

Plant materials

To investigate the genome of *Taxus wallichiana*, fresh leaves were collected from a female Himalayan yew tree, estimated to be at least 50 years old, cultivated at the Kunming Institute of Botany, Chinese Academy of Sciences. For RNA sequencing (RNA-seq), fresh leaves and fruits were collected from the same tree.

Sequencing

Sample collection was based on precise handling protocols aimed at extracting high-molecular-weight genomic DNA from Himalayan yew tissues. Initially, DNA extraction was performed using the CTAB method, followed by purification with the QIAGEN® Genomic kit (catalog number 13343, QIAGEN) to ensure the DNA was suitable for conventional sequencing analysis. Library Preparation and Sequencing: Library preparation followed PacBio's standard protocol for HiFi target libraries, using a 15 kb preparation scheme. Sequencing was performed on a PacBio Sequel II instrument using Sequencing Primer V2 and the Sequel II Binding Kit 2.1 at Haorui Genomics. For Nanopore sequencing, genomic DNA (gDNA) samples were extracted from Himalayan yew young leaves using the QIAGEN® Genomic DNA extraction kit (catalog number 13323, QIAGEN). DNA purity was measured with a NanoDrop™ One UV-Vis spectrophotometer (Thermo Fisher Scientific, USA), with OD260/280 ratios between 1.8 and 2.0 and OD260/230 ratios between 2.0 and 2.2. DNA libraries

were subsequently loaded into the pre-assembled flow cells of the Nanopore PromethION sequencer (RRID:SCR_017987; Oxford Nanopore Technologies, UK) for sequencing.

Genome assembly and quality assessment

Using the hifiasm software (Hifiasm 0.24.0-r702, RRID:SCR_021069) [21, 22], we integrated ONT UL sequencing data, HiFi sequencing data and Hi-C sequencing data to perform the phased assembly of the *T. wallichiana* genome (parameter: hifiasm -l 0). Using BWA software (BWA 0.7.17-r1198-dirty, RRID:SCR_010910) [23, 24], we aligned the Hi-C data to the genome which is merged by two haplotype assemblies. The HapHiC pipeline [25, 26] was then employed to scaffold the contigs to the chromosome level, followed by manual correction of any assembly errors. The completeness of the genome was assessed using BUSCO (BUSCO 5.3.2, RRID:SCR_015008) [27, 28]. Merqury software (Merqury 1.3, RRID:SCR_022964) [29, 30] was used to evaluate the genome's completeness and the error rate of each chromosome and the overall error rate using HiFi reads. HiFi reads and ONT UL reads were aligned to the reference genome using minimap2 software (Minimap2 2.26-r1175, RRID:SCR_018550) [31, 32] to assess depth distribution and to evaluate haplotype depth distribution based on HiFi reads.

Genome annotation

We used EDTA (EDTA 2.0.1, RRID:SCR_022063) [33, 34] to construct a high-quality, non-redundant repeat sequence library. Gene prediction was performed by BRAKER3 (BRAKER3 3.0.8, RRID:SCR_018964) [35-40] using the soft-masked genome. Annotation was performed using protein sequences from 20 gymnosperms and about 300Gb of transcriptome data (Supplementary Table S1). Transcriptome sequence alignment and assembly were performed using HISAT (HISAT 2.2.1, RRID:SCR_015530) [41-43]. The annotation results were evaluated using BUSCO (BUSCO 5.3.2, RRID:SCR_015008) [27, 28] with the Gymnosperm_odb10 and embryophyta_odb10 datasets. All protein-coding genes were retrieved and

functionally annotated by blast searches against databases including UniProtKB/Swiss-Prot, UniProtKB/TrEMBL, NR, KEGG. They were also subjected to GO annotation and protein family annotation by InterProScan (InterProScan 5.45-80.0, RRID:SCR_005829) [44, 45].

Mining ODD enzymes

ODD gene family proteins were identified across the entire genome using tblastn [46]. We downloaded a publicly available dataset containing 40 transcriptome samples, including different cell lines with high and low paclitaxel yields, as well as five tissue types. Transcriptome data alignment and quantification were performed using HISAT (HISAT 2.2.1, RRID:SCR_015530) [43, 47] and StringTie (StringTie 2.1.7, RRID:SCR_016323) [48, 49]. Next, we calculated the expression correlation matrix between ODD and paclitaxel synthesis-related genes. Clustering was applied to this matrix, and ODD genes within the cluster containing the highest number of known paclitaxel synthesis genes were selected as candidate genes for subsequent experimental validation.

ODD enzyme activity assay

The ODD genes to be validated were codon-optimized for *Saccharomyces cerevisiae* (*S. cerevisiae*) and then cloned into the yeast expression vector Ycplac22 using the Gibson Assembly method. This constructed expression vector was subsequently transformed into our pre-engineered *S. cerevisiae* chassis strain [4] for cytoplasmic taxa-4(5),11(12)-diene(taxadiene) production, where it was expressed and functionally analyzed. Using the Gibson Assembly technique, we assembled the YCPlac22 vector, a bidirectional terminator, a bidirectional strong promoter, and the candidate ODD gene sequences together. The recombinant plasmids were sequenced for validation and then transformed into host cells producing taxadiene.

To detect the synthesis of taxadiene and the activity of ODD enzymes, *S. cerevisiae* strain was first cultured in 3 mL of defective medium in test tubes for 48 hours at 30°C and 800 rpm. The seed culture was then inoculated into 40 mL of fresh

medium at a ratio of 1:50. After 10 hours of cultivation, 5 mL of n-dodecane and 2 mL of 40% glucose were added to initiate two-phase fermentation, promoting product separation and accumulation. This cultivation process continued for 4 days at 30°C and 220 rpm. Subsequently, the upper organic phase was collected by centrifugation at 3600 rpm for 10 minutes for GC-MS analysis.

For GC-MS analysis, samples were analyzed using a Thermo Scientific TRACE 1600 coupled with a TSQ 9000 triple quadrupole mass spectrometer. A 2 μL sample was injected into a TG-1MS GC column (30 m × 0.25 mm × 0.25 μm). The initial column temperature was set to 80°C and held for 1 minute, followed by an increase to 200°C over 7 minutes. The injection port and transfer line temperatures were set to 300°C and 290°C, respectively, to ensure efficient sample injection and transfer.

Mechanistic analysis of ODD enzymes

Structure Preparation: All ODD enzyme structures in this study were modeled using OpenFold (OpenFold 2.0) [50, 51] with default parameters. The top-ranked structure based on pLDDT scores was selected for further analysis. Substrate molecule structures were obtained from PubChem (CID: 167825), and the coenzyme alpha-ketoglutarate structure was also sourced from PubChem (CID: 51).

Molecular Docking: All molecular docking procedures were performed using the Watvina method (Watvina, RRID:SCR_026282) [52]. AutoDock Tools (AutoDock Tools 1.5.6, RRID:SCR_012746) [53-55] was used to prepare the substrate molecules and protein receptors. The docking box was defined as a cubic box with a side length of 40 nm. The docking energy range was set to 5 kcal/mol, with an exhaustiveness parameter of 12 and a maximum of 100 output conformations. Reasonable conformations were identified based on the distance between the substrate reactive site and the iron-oxo (FeO) center being within 5 Å, and a negative docking score. From these reasonable conformations, the most appropriate one was selected for further structural analysis based on manual assessment.

Molecular Dynamics Simulation: Molecular dynamics simulations were conducted using Gromacs (Gromacs 2023.2, RRID:SCR_014565) [56-58]. The

AMBERff14SB force field parameters were applied. Small molecules were converted into GROMACS-compatible ITP-format topology files using the AmberTools toolkit (AmberTools 24, RRID:SCR_018497) [59, 60], and the GAFF force field was used to parameterize ligand atoms [61], with parameters generated by the Antechamber tool (Amber 20, RRID:SCR_014230) [59, 62]. The TIP3P model was used for water molecules. The protein-ligand complex was placed in a cubic periodic water box with a minimum boundary distance of 10 Å. Sodium (Na^+) and chloride (Cl^-) ions were added to neutralize the system. Long-range electrostatic interactions were handled using the Ewald method. The system's energy was minimized using the steepest descent method for a maximum of 5000 steps. Subsequently, the system was equilibrated with 100 ps of NVT simulation followed by 100 ps of NPT simulation. The production dynamics simulation was then run at 300 K and 1 bar pressure with periodic boundary conditions, for a duration of 100 ns with a time step of 2 fs. Energy, trajectory, and structural data were collected every 1 ps. RMSD, RMSF, and other analyses were performed using the built-in trajectory analysis modules of Gromacs (Gromacs 2023.2, RRID:SCR_014565) [56-58].

Results

Phased high-quality genome of *Taxus wallichiana* (Twv1)

The PacBio Revio platform and the Oxford Nanopore Technologies (ONT) ultra-long (UL) sequencing technology were employed to conduct further sequencing of the same individual of the Himalayan yew as previously reported by Cheng et al. [4]. This effort yielded a total of approximately 722 Gb (72.2×) of HiFi reads, approximately 875 Gb (87.5×) of ONT UL reads and 1077.1 Gb (~100×) Hi-C reads. Among these, the N50 length of HiFi reads exceeded 16 kb, while the N50 length of ONT reads approached 54 kb (Supplementary Tables S2, S3, S4). Through the integration and assembly of these data using the hifiasm software (Hifiasm 0.19.8-r602, RRID:SCR_021069) [21, 22], we obtained a phased assembly with two haptypes.

Utilizing the Hi-C data, we anchored the contigs of both haplotypes onto 24 chromosomes, achieving an anchoring rate of 97.5%. The continuity of the Hi-C heatmap indicates that the genome has no large-scale assembly errors and that the two haplotypes were correctly phased (Fig. 1A, Table 1). As a result, we obtained a high-quality haplotype-resolved genome, TWv1, with a total genome size of approximately 20.3 Gb, consisting of 24 chromosomes and a contig N50 length of 169.4 Mb (Table 1). Each chromosome contains complete telomeres at both ends, and the TWv1 genome includes only 201 gaps (Fig1B, Supplementary Table S5). The genome sizes of haplotype 1 (TWv1-hap1) and haplotype 2 (TWv1-hap2) are 9.87 Gb and 9.98 Gb, respectively.

Multiple approaches were employed to assess the completeness and continuity of the TWv1 genome. For completeness assessment using *K*-mer based method performed by Merqury software (Merqury 1.3, RRID:SCR_022964) [29,30] revealed a genome quality value (QV) of approximately 59.6, with QV values for individual chromosomes ranging from 57.22 to 64.06 (Table 1; Supplementary Table S6). The overall completeness is 99.36%, which corresponds to approximately one base error per 1 Mbp (Table 1). For the BUSCO assessment of genome completeness, both haplotypes achieved over 95% completeness in the Gymnosperm_odb10 dataset, while in the Embryophyta_odb10 dataset, both haplotypes reached approximately 90%. This difference may be attributed to the lower suitability of the Embryophyta_odb10 dataset for gymnosperms (Supplementary Table S7) [63]. The Hi-C heatmap also demonstrated the continuity of the TWv1 genome assembly, further confirming the high precision of the assembly quality (Fig. 1A). The TWv1 genome assembly corrected 12 large-scale assembly errors present in earlier versions, including 11 intrachromosomal assembly errors in *T. wallichiana* (as TWv0 in this study), where the two arms of the chromosomes exhibited "inversion" assembly errors (Fig. 1D) [4]. Additionally, a potential "inversion" assembly error was identified in the chromosome of *Taxus chinensis var. mairei* (TCv0) [5], with coordinates consistent with those in TWv0. However, due to the different species and the inability to identify telomeric sequences in TCv0, this could also be attributed to interspecies differences

[5]. In conclusion, we have successfully completed the first phasing of the Himalayan yew genome and addressed its haplotype assembly issues. We have also significantly reduced the number of gaps in the Himalayan yew genome. Compared to previous versions (TWv0), the new high-quality haplotype genome TWv1-hap1 has only 85 remaining gaps, and TWv1-hap2 has only 116 gaps, which is a substantial improvement over the TCv0 and *Taxus yunnanensis* (TYv0) genomes [64], which recorded 12,092 and 11,130 gaps, respectively [5, 64]. Comparative analysis of contig N50 lengths showed that the assembly quality of TWv1 far surpasses that of 23 other gymnosperm genomes (Fig. 1C; Supplementary Table S1), with Contig N50 lengths 19.7 times, 69.4 times, and 58.6 times greater than those of TWv0, TCv0, and TYv0, respectively (Table 1). Importantly, the genome of Twv1 has identified all 24 telomeres (Supplementary Table S8). Since chr2 of haplotype 1 has no gaps and the telomeres are complete, its assembly has reached the T2T (telomere-to-telomere) level. This chromosome, with a length of 1.01 Gb, is the longest T2T chromosome reported to date.

We also obtained high-precision annotation for the TWv1 genome. Using EDTA software (EDTA 2.0.1, RRID:SCR_022063) [33, 34], we annotated the composition and precise locations of repetitive sequences in Himalayan yew. In the TWv1 genome, the repetitive sequences of TWv1-hap1 and TWv1-hap2 account for 8.3 and 8.4 Gb, respectively, making up 84% of the total genome. Among these, long terminal repeat (LTR) elements are the most abundant type of repetitive sequence, comprising 69% of all repetitive sequences in both haplotypes, and representing 58% of the total genome in TWv1-hap1 and TWv1-hap2. DNA transposons account for more than 19% of both haplotype genomes (Supplementary Tables S9, S10). Additionally, we predicted 37,766 and 38,579 protein-coding genes in the TWv1-hap1 and TWv1-hap2 genomes, respectively. The average coding sequence length is 1,119 bp and 1,111 bp, with each gene containing an average of 4.44 and 4.38 exons, and average exon lengths of 252.27 bp and 253.34 bp, respectively (Supplementary Tables S9, S10). We performed a detailed analysis of the GC content, gene numbers, LTR-Gypsy/Copia distribution, and TE distribution in the TWv1-hap1 genome using 500 kb windows on

each of the 12 chromosomes. This information was comprehensively plotted into a circos diagram of the TWv1-hap1 genome (Fig. 1E).

Genetic polymorphism analysis and sex differentiation in Himalayan yew

To investigate the genetic polymorphism of Himalayan yew, we identified structural variations (length > 50 bp) between the two haplotype genomes in TWv1 (Fig. 2). The analysis revealed that the total length of all structural variations accounts for 13.4% of the entire genome, including 64 duplications, 66 translocations, and 267 inversions (Supplementary Tables S11, S12). We found four ultra-large structural variations greater than 100 Mb in length between the two haplotypes, including a 366 Mb interchromosomal translocation between chr4 and chr8, and inversions of 169 Mb and 148 Mb on chr6 and chr9, respectively (Fig. 2A, Supplementary Fig. S1, Supplementary Table S13). These events may pose certain obstacles to homologous chromosome recombination during meiosis in this individual. Additionally, chromosome length is a characteristic of variation between haplotypes. To avoid misjudgment of chromosome length due to assembly errors, we evaluated the collapse regions of the genome based on the mapping depth of HiFi reads, using a 100 Kb window (Supplementary Table S14). Statistics indicate that a total of 218 Mb of regions in the genome exhibit collapse and they only distributed in chr4, chr7, chr8, with a collapse rate of 1.07% (Supplementary Fig. S2). The total length of collapsed sequences in TWv1-hap1 is 42 Mb, with a collapse rate of only 0.42%. By summing the collapsed lengths and assembled lengths of each chromosome, we predicted the actual lengths of Himalayan yew chromosomes. Comparisons of lengths between all pairs of homologous chromosomes show that the length differences between haplotypes range from 0.12% to 1.71%. Aside from the chromosomal translocations observed in chr4 and chr8, the largest length difference is found in chr12, with a sequence length difference of 7.2 Mb, accounting for 1.71% and 1.68% of the chr12 of two haplotypes, respectively. This length difference is caused by the 0-28 Mb region of chr12.2 and the 0-16 Mb region of chr12.1 (Fig. 2B, Supplementary Table

S14). According to previous karyotype studies [65], this pair of chromosomes also exhibits length differences, and was considered as the candidate sex chromosomes. Given that the individual assembled in this study is female as indicated by its yield of seeds (Supplementary Fig. S3), this result suggests that the sex determination in Himalayan yew might follow the ZW model, with chr12.1 and chr12.2 representing the candidate Z and W chromosomes, respectively (Fig. 2B). The identification of a Dof zinc finger protein family gene, which has been found being involved in flowering control [66, 67], in the highly variable regions between the two homologous chromosomes further suggests they may the sex chromosomes. However, more direct evidence is needed to validate this hypothesis.

To further investigate sex differentiation in Himalayan yew, we compared the Z and W chromosomes and identified structural variations between homologous chromosomes. We found that the 0-30 Mb region of the W chromosome and the 0-16 Mb region of the Z chromosome contain 10.0 Mb (33.3%) and 4.0 Mb (25.0%) of unaligned regions, respectively. In these non-homologous regions, the Z and W chromosomes have undergone five duplications of approximately 4.0 Mb, and inversions of 3.7 Mb and 1.0 Mb, respectively (Fig. 2B, Supplementary Tables S11, S12). Using existing transcriptome data of roots, leaves, bark, male flowers, and female flowers from the *Taxus spp.* in the database [5], we identified seven genes in these regions that are expressed and exhibit sex-specific expression in flower (Fig. 2C). Interestingly, the TW12H1G0005v3 gene on the W chromosome is highly expressed only in the flower of female individuals but is lowly expressed or not expressed in male individuals (Fig. 2C). The TW12H1G0005v3 gene has been identified as belonging to the Dof zinc finger protein family - CDF2 (cycling Dof factor 2). CDF proteins are a unique class of transcription factors in the plant DOF family. Studies have shown that CDF transcription factors play a crucial role in photoperiod response for flowering control in *Arabidopsis* [66, 67]. These results suggest that TW12H1G0005v3 may be related to sex differentiation.

Mining ODD enzymes in Himalayan Yew

ODDs play a critical role in the biosynthesis of a wide range of specialized metabolites in plants. Based on amino acid sequence similarity, ODDs are typically classified into three main subfamilies: DOXA, DOXB, and DOXC. The DOXA class serves as the prototype of ODDs and is involved in the alkylation and oxidative demethylation of nucleic acids and histones. The DOXB class is conserved across all plant taxa and is involved in the proline 4-hydroxylation in cell wall protein synthesis. The DOXC class ODD enzymes are of particular interest due to their role in the specialized metabolism of various plant chemicals, including phytohormones and flavonoids. Most ODDs in terrestrial plants belong to the DOXC class [18]. In conjunction with previous studies and the powerful and unique functions of ODD enzymes [17], we hypothesize that ODD enzymes may catalyze the epoxidation of taxadiene, the initial taxane substrate in the paclitaxel biosynthesis pathway (Fig. 3A). To this end, we conducted a comprehensive classification and mining of the ODD family genes in the Himalayan yew genome. We identified the DOXC members in the Himalayan yew ODDs and constructed a gene family tree along with arabidopsis, maize, rice, and tobacco (Fig. 3B). The Himalayan yew has 158 genes belonging to the ODD DOXC family, with many genes formed by tandem repeat expansions (Fig. 3C, Supplementary Table S15). The expansion of these genes in the Himalayan yew suggests their potential role in specific phenotypes, such as paclitaxel synthesis.

To further identify candidate genes and validate our hypothesis, we utilized multi-tissue transcriptome data from public databases. This dataset includes multiple cell lines with high and low taxol production, as well as five types of tissues (bark, root, leaf, strobilus, and strobili) from both male and female trees, totaling 40 samples. We calculated the gene expression similarity matrix for ODD DOXC family genes and known paclitaxel biosynthesis-related genes across these samples (Fig. 3D). Notably, the DOXC family genes and paclitaxel synthesis-related genes formed a co-expression module comprising 16 known synthesis genes and 70 ODD family genes. We hypothesize that these genes possess potential taxadiene epoxidase activity. Using multiple sequence alignment and phylogenetic tree analysis, along with

consideration of different evolutionary clades and the chromosomal distribution of these genes, we selected 11 genes as candidates for experimental validation (Fig. 3C, Supplementary Table S16).

Activity validation and analysis of ODD enzymes

To investigate the functional roles of the selected ODD genes in the paclitaxel biosynthesis pathway, we introduced 11 candidate genes into the yeast cytosolic taxadiene production chassis we previously constructed [4]. GC-MS activity validation results showed that 9 out of the 11 genes exhibited potential C4β-C20 epoxidation activity (Fig. 3E, Supplementary Fig. S4), while sequence 9 and sequence 15 did not display any catalytic activity.

To analyze the epoxidation activity of the ODD enzymes, we performed molecular dynamics simulations on the 11 experimentally validated enzymes. Since our previously constructed yeast cytoplasmic taxadiene production chassis primarily produces endotaxadiene (taxa - 4 (5), 11 (12) - diene), we further studied the catalytic distance of endotaxadiene (from C11 to Fe in FeO) within the catalytic cavity. The results indicated that enzymes with catalytic activity generally had shorter catalytic distances, while those without catalytic activity showed longer catalytic distances (Fig. 3F). Subsequently, we performed multiple sequence alignment of the 11 enzymes and analyzed their three-dimensional structures. We found that the catalytic cavities of sequences 9 and 15, which lacked epoxygenase activity, had an additional 17 amino acids (in yellow) compared to the other sequences with catalytic activity (Fig. 3G). This extra sequence might be the reason for the different catalytic activities.

By comparing the catalytic cavities of sequences 10 and 15, we discovered that the insertion of these 17 amino acids in sequence 15 appeared as loop 84-101 protruding into the catalytic cavity in the three-dimensional structure (Fig. 3H). This loop formed hydrogen bonds with other residues in the catalytic cavity, specifically D91-R319 (63.2%), D99-R183 (96.5%), and K101-G200 (86.3%). These hydrogen bonds stabilized the position of the loop, and the loop occupied space within the

catalytic cavity, making it difficult for the substrate to bind with the coenzyme and FeO within the cavity. Additionally, the presence of this loop likely hindered the entry and exit of the substrate from the catalytic cavity. In summary, we hypothesize that the lack of epoxygenase activity in sequences 9 and 15 is likely due to the presence of this loop, which obstructs further interaction between the substrate and ODD. We also measured the catalytic pocket sizes of sequences 10 and 15 (Fig. 3I), finding that the catalytic pocket of sequence 15, which lacked catalytic activity, was only 124Å^3 due to the loop insertion, whereas the catalytic pocket of sequence 10, which did not have the loop, was 182Å^3, further supporting our hypothesis.

Collinearity and P450 analysis of TWv1

Taxus spp. is the only large-scale source of the anti-cancer drug paclitaxel [1]. *Torreya grandis*, a gymnosperm of the Cephalotaxaceae family, is closely related to *Taxus*. Recent research shows that *Torreya grandis* separated from *Taxus wallichiana* around 68.5 million years ago [68], and *Torreya grandis* produces little to no paclitaxel [69]. Comparative studies among closely related species are one method to explore the evolutionary history of genes. To investigate the evolutionary trajectory of the paclitaxel biosynthesis pathway, we examined the collinearity relationship between the positions of CYP450 genes in *T. wallichiana* (TWv1) and *Torreya grandis* (Fig. 4A, 4B). In *T. wallichiana*, the paclitaxel biosynthesis gene cluster is concentrated in the 19.76-27.16 Mb region of chr9. This region shows good collinearity with the 79.38-104.19 Mb region of chr4 in *Torreya grandis*. However, there are no direct homologs of the CYP450 genes within the paclitaxel biosynthesis gene cluster in *Torreya grandis* (Fig. 4C). This suggests that the emergence of the paclitaxel biosynthesis gene cluster occurred after the divergence of these two species, which is after 68.5 million years ago.

The expansion of the CYP725A subfamily may have played a crucial role in the evolution of paclitaxel biosynthesis. Utilizing high-quality genomic data, our research group performed a comprehensive analysis of the classification of all CYP450 genes

in *T. wallichiana* by comparing them to the CYP450 database using standard sequence similarity cutoffs [5] (Fig. 4D), and precisely identified their copy numbers (Fig. 4E, Supplementary Table S17). We found that 84% of the CYP725A subfamily members (54 out of 64) and 10 key paclitaxel biosynthesis genes (such as TXS, T5αOH, T10βOH, T13αOH, T2αOH, T7βOH, DBAT, T9αOH, T1βOH, and TOT1) are predominantly clustered in specific regions on chr9, showing significant aggregation (Fig. 4D, Supplementary Table S18).

Discussion

Gymnosperms, as a unique plant lineage, typically have very large genomes, often exceeding 1 billion base pairs per haploid genome, which poses significant challenges for genome assembly [9]. The large genome size not only increases the data requirements for sequencing but also results in a higher proportion of repetitive sequences and complex structural variations. These factors complicate the assembly process, increasing the error rate and uncertainty. Additionally, large genomes require more computational resources and more complex algorithms to handle the data. Therefore, despite advances in sequencing technology, the assembly of gymnosperm genomes remains a complex and arduous task. For example, the genomes of the gymnosperms *Cycas panzhihuaensis* and *Ginkgo biloba* are both in the range of 10-12 Gb and have over 70% repeat sequences [3, 70]. In this study, the repetitive sequences in *T. wallichiana* (TWv1) account for 84%. We overcame the obstacles of large genome size and high levels of repetitive elements to generate the first phased high-quality gymnosperm genome for *T. wallichiana*, providing valuable genomic resources for future gymnosperm research.

Dioecy is a major characteristic of gymnosperms, present in 667 out of 1033 species (64.6%) [71]. Dioecy has evolved repeatedly from monoecy in gymnosperms, with 10-13 independent evolutions in the *Pinopsida* alone [72, 73]. Sex chromosomes have been studied in 6 species (0.6% of the total) in the genera *Cycas*, *Zamia*, *Stangeria*, *Ephedra*, *Podocarpus*, and *Ginkgo* [74]. In *Cycas revoluta*, males exhibit

significant sex chromosome size differences, with the 22nd median chromosome being much shorter than the 21st submetacentric chromosome, while in females, both chromosomes are submetacentric and of equal length, showing an XX/XY type of sex determination [75]. Early studies on the sex determination of *Ginkgo biloba* reported both XY and ZW sex chromosome systems [76-80]. Therefore, further research is needed to clarify the exact sex determination system in *Ginkgo biloba*. In this study, we inferred the candidate sex chromosomes of *T. wallichiana* based on chromosome length and sex-biased expression data. Although we have made some preliminary progress, further in-depth research is needed to resolve the sex determination mechanism of *T. wallichiana*.

ODDs play an irreplaceable role as oxygenases widely involved in biosynthetic processes in plants [18], but their role in *T. wallichiana* is not yet fully understood. This study analyzed candidate ODDs screened from *T. wallichiana*. The experimental results demonstrated that certain ODD genes in *T. wallichiana* are capable of epoxidizing endotaxadiene. Molecular dynamics simulations further supported the catalytic roles of these genes in forming epoxides within the paclitaxel biosynthetic pathway. Whether ODDs can further promote the formation of the oxetane ring in taxane based on epoxides requires more in-depth research in the future. Cytochrome P450s (CYP450s) play crucial roles in the biosynthesis of the diterpene compound paclitaxel in the *Taxus spp.*. However, the paclitaxel biosynthetic pathway and its enzymes are very complex, and some specific CYP450 enzymes remain unclear. Based on high-quality genomic data, this study conducted an in-depth analysis of the CYP450 gene family in *T. wallichiana*. We successfully identified and annotated several CYP450 genes potentially involved in the paclitaxel biosynthetic pathway. Not only did we clarify the gene clusters related to paclitaxel biosynthesis on the chromosomes, but we also identified potential candidate gene clusters. This achievement provides a solid foundation for subsequent verification of these genes' precise roles in paclitaxel synthesis.

In recent years, many studies have conducted transcriptome-wide identification of CYP450s involved in terpenoid biosynthesis [81], including CYP716A47 related to

ginsenoside biosynthesis [82], CYP76AH1 catalyzing the conversion of miltiradiene in tanshinone biosynthesis [83], and the recently identified CYP725A4, CYP725A37, and CYP725A55, which can catalyze the formation of the oxetane ring in taxane [15, 19, 20]. It is widely recognized that the *Taxus spp.* is the only large-scale source of paclitaxel. However, there has been long-standing controversy regarding whether other closely related species in gymnosperms (such as *Torreya grandis* and *Ginkgo*) can produce paclitaxel. Due to the difficulty of obtaining high-quality gymnosperm genomes, there has been a lack of genetic evidence to support existing experiments or hypotheses. In this study, collinearity analysis between the high-quality genome of *T. wallichiana* (TWv1) and the recently published genome of *Torreya grandis* provided evidence explaining why *Torreya grandis* produces little to no paclitaxel [69]. Furthermore, future comparative studies with other species from the same family and gymnosperms will provide a crucial foundation for understanding the genomic evolution and sex determination mechanisms in gymnosperms.

Data Availability

All sequencing data supporting the findings of this study, as well as the genome assemblies, are available at the National Center for Biotechnology Information (NCBI) under accession number PRJNA1146068. The metabolite detection data in this study is available at the NIH Common Fund's National Metabolomics Data Repository (NMDR) website, the Metabolomics Workbench, where it has been assigned Project ID PR002330. The data can be accessed directly via its Project DOI [84]. All supporting data and materials are available in the *GigaScience* GigaDB database [85].

List of Abbreviations

T2T: telomere to telomere;

TWv1: The high-quality genome assembled in this study;

ZW: A sex-determination model, where females are heterogametic (ZW), and males are homogametic (ZZ);

ODD: 2-oxoglutarate/Fe(II)-dependent dioxygenase;
CYP450s: Cytochrome P450 enzymes;
RNA-seq: RNA sequencing;
ONT UL: Oxford Nanopore Technologies Ultra Long;
PacBio HiFi: Pacific Biosciences high fidelity;
gDNA: genomic DNA
Hi-C: high-throughput chromosome conformation capture;
bp: base pairs;
Mbp/Mb: megabase pairs;
Kb: kilobase pairs;
Gb: gigabase pairs;
BLAST: Basic Local Alignment Search Tool;
BUSCO: Benchmarking Universal Single-Copy Orthologs;
CDS: coding sequence;
ODD: 2-oxoglutarate/Fe(II)-dependent dioxygenase;
GO: Gene Ontology;
UniProtKB/Swiss-Prot: Universal Protein Knowledgebase/Swiss-Prot;
UniProtKB/TrEMBL: Universal Protein Knowledgebase/Translated European Molecular Biology Laboratory;
Nr: NCBI’s nonredundant database;
KEGG: Kyoto Encyclopedia of Genes and Genomes;
QV: quality value;
Gymnosperm_odb10: Gymnosperm OrthoDB v10 (a database of orthologous genes for gymnosperms);
Embryophyta_odb10: Embryophyta OrthoDB v10 (a database of orthologous genes for land plants);
LINE: long interspersed nuclear element;
LTR: long terminal repeat;
Mya: million years ago;
SINE: short interspersed nuclear element;

snRNA: small nuclear RNA;

SNP: single nucleotide polymorphism;

SNR: signal-to-noise ratio;

SV: structural variation;

TE: transposable element;

TRF: tandem repeat;

CDF2: cycling Dof factor 2.

Additional Files

Supplementary Fig S1. Collinear Features of TWv1.

Supplementary Fig S2. Genomic Features of TWv1.

Supplementary Fig S3. The yew sequenced in our study.

Supplementary Fig S4. Gas Chromatography-Mass Spectrometry (GC-MS) Results.

Supplementary Table S1. 23 other Gymnospermae genomes used in figure 1C.

Supplementary Table S2. Summary of sequencing data.

Supplementary Table S3. Summary of hifi sequencing data.

Supplementary Table S4. Summary of nanopore sequencing data.

Supplementary Table S5. Gap status of Twv1.

Supplementary Table S6. Assembly quality of TWv1.

Supplementary Table S7. BUSCOs analysis of TWv1 genome completeness.

Supplementary Table S8. Survey results of telomeric sequence.

Supplementary Table S9. Gene annotation.

Supplementary Table S10. Summary of transposable elements.

Supplementary Table S11. Structural variation(SV) in the Twv1 genome.

Supplementary Table S12. Structural variation(SV) statistics.

Supplementary Table S13. Large-scale chromosomal structural variation.

Supplementary Table S14. Collapse status of Twv1.

Supplementary Table S15. Distribution and Quantity of Major ODDs in Twv1.

Supplementary Table S16. 11 Sequences Selected through Co-Expression Network Analysis.

Supplementary Table S17. Statistical analysis of CYP450s in Twv1.

Supplementary Table S18. Mapping of Characterized Enzymes in the Biosynthetic Pathway of Paclitaxel on the Himalayan Yew Genome.

Competing Interests

A provisional Chinese patent application regarding the epoxidase and the isolation of taxane intermediates has been filed by Jiaxing Synbiolab Biotechnology Co., Ltd (application numbers 2024108232477 and 2024108230556), with X.W., Q.H.D. and X.Q.Ch listed as inventors. All authors declare that they have no other competing interests.

Author Contributions

W.W., H.F.J. and X.W. designed the study. G.C.L., H.Z. and Z.Z.L. prepared materials for genomic and RNA-seq analysis. H.Z., S.M.L., X.J., Y.Q.W., Z.Y.L., Z.K.W., B.T.Zh., Z.H.L., L.Z. and J.C. performed genomic analysis and evolutionary analysis. H.W. and J.B. performed kinetic simulations of ODD enzyme analysis. Z.Z.L., X.N.L, X.Q.Ch., J.L., X.X.Zh, Q.H.D., Y.L., and Y.Y. performed experiments. Z.Z.L., H.Z., X.J., S.M.L. and X.Q.Ch. wrote the manuscript.

Fundings

This research was funded by The New Cornerstone Science Foundation (to WW) and the National Natural Science Foundation of China (Grant No. 32371499). Additionally, computational resources were provided by the High-Performance Computing Platform of Jiaxing Synbiolab Biotechnology Co., Ltd.

Acknowledgments

We thank Ruoping Zhao from Kunming Institute of Botany, Chinese Academy of Sciences for providing plant samples; Botong Zhou, Zihe Li and Wenbo Zhu from

School of Ecology and Environment, Northwestern Polytechnical University for data analysis.

References

1. Das B,Anjani G. Chemical Constituents of the Himalayan Yew, A Review. Nat Prod Sci 1998;4(4):185-202. https://koreascience.kr/article/JAKO199803041122562.pdf
2. De La Torre AR, Birol I, Bousquet J, et al. Insights into conifer giga-genomes. Plant Physiol 2014;166(4):1724-32. https://doi.org/10.1104/pp.114.248708.
3. Liu Y, Wang S, Li L, et al. The Cycas genome and the early evolution of seed plants. Nat Plants 2022;8(4):389-401. https://doi.org/10.1038/s41477-022-01129-7.
4. Cheng J, Wang X, Liu X, et al. Chromosome-level genome of Himalayan yew provides insights into the origin and evolution of the paclitaxel biosynthetic pathway. Mol Plant 2021;14(7):1199-209. https://doi.org/10.1016/j.molp.2021.04.015.
5. Xiong X, Gou J, Liao Q, et al. The Taxus genome provides insights into paclitaxel biosynthesis. Nat Plants 2021;7(8):1026-36. https://doi.org/10.1038/s41477-021-00963-5.
6. Zhang Y, Scossa F,Fernie AR. The genomes of Taxus species unveil novel candidates in the biosynthesis of taxoids. Mol Plant 2021;14(11):1773-5. https://doi.org/10.1016/j.molp.2021.08.017.
7. Ohri D,Rastogi S. Sex determination in gymnosperms. The Nucleus 2020;6375-80. https://doi.org/10.1007/s13237-019-00297-w.
8. Gorelick R. Theory for why dioecious plants have equal length sex chromosomes. Am J Bot 2005;92(6):979-84. https://doi.org/10.3732/ajb.92.6.979.
9. Wan T, Gong Y, Liu Z, et al. Evolution of complex genome architecture in gymnosperms. GigaScience 2022;11giac078. https://doi.org/10.1093/gigascience/giac078.
10. Srinivasan V, Pestchanker L, Moser S, et al. Taxol production in bioreactors: kinetics of biomass accumulation, nutrient uptake, and taxol production by cell suspensions of *Taxus baccata*. Biotechnol Bioeng 1995;47(6):666-76. https://doi.org/10.1002/bit.260470607.
11. Choy H. Taxanes in combined-modality therapy for solid tumors. Oncology (Williston Park, NY) 1999;13(10 Suppl 5):23-38. https://doi.org/10.1016/s1040-8428(00)00112-8.
12. Khayat D, Antoine E-C,Coeffic D. Taxol in the management of cancers of the breast and the ovary. Cancer Invest 2000;18(3):242-60. https://doi.org/10.3109/07357900009031828.
13. Zhang CH, Fevereiro PS, He G, et al. Enhanced paclitaxel productivity and release capacity of *Taxus chinensis* cell suspension cultures adapted to chitosan. Plant Sci 2007;172(1):158-63. https://doi.org/10.1016/j.plantsci.2006.08.002.
14. Nazhand A, Durazzo A, Lucarini M, et al. Rewiring cellular metabolism for heterologous biosynthesis of Taxol. Nat Prod Res 2020;34(1):110-21. https://doi.org/10.1080/14786419.2019.1630122.
15. Yang C, Wang Y, Su Z, et al. Biosynthesis of the highly oxygenated tetracyclic core skeleton of Taxol. Nat Commun 2024;15(1):2339. https://doi.org/10.1038/s41467-024-46583-3.

16. Wang T, Li L, Zhuang W, et al. Recent research progress in taxol biosynthetic pathway and acylation reactions mediated by Taxus acyltransferases. Molecules 2021;26(10):2855. https://doi.org/10.3390/molecules26102855.
17. Zhang Y, Wiese L, Fang H, et al. Synthetic biology identifies the minimal gene set required for paclitaxel biosynthesis in a plant chassis. Mol Plant 2023;16(12):1951-61. https://doi.org/10.1016/j.molp.2023.10.016.
18. Kawai Y, Ono E,Mizutani M. Evolution and diversity of the 2-oxoglutarate-dependent dioxygenase superfamily in plants. The Plant Journal 2014;78(2):328-43. https://doi.org/10.1111/tpj.12479.
19. Zhao Y, Liang F, Xie Y, et al. Oxetane Ring Formation in Taxol Biosynthesis Is Catalyzed by a Bifunctional Cytochrome P450 Enzyme. J Am Chem Soc 2024;146(1):801-10. https://doi.org/10.1021/jacs.3c10864.
20. Jiang B, Gao L, Wang H, et al. Characterization and heterologous reconstitution of Taxus biosynthetic enzymes leading to baccatin III. Science 2024;383(6681):622-9. https://doi.org/10.1126/science.adj3484.
21. Feng X, Cheng H, Portik D, et al. Metagenome assembly of high-fidelity long reads with hifiasm-meta. Nat methods 2022;19:671-674. https://doi.org/10.1038/s41592-022-01478-3.
22. Hifiasm (2024). Hifiasm (Version 0.24.0-r702) https://github.com/chhylp123/hifiasm.
23. Li H. Aligning sequence reads, clone sequences and assembly contigs with BWA-MEM. arXiv 2013. https://doi.org/10.48550/arXiv.1303.3997.
24. BWA (2020). BWA (Version 0.7.17-r1198-dirty) http://bio-bwa.sourceforge.net/.
25. Zeng X, Yi Z, Zhang X, Du Y, Li Y, Zhou Z, et al. Chromosome-level scaffolding of haplotype-resolved assemblies using Hi-C data without reference genomes. Nature Plants. 2024;10 8:1184-200. https://doi.org/10.1038/s41477-024-01755-3.
26. HapHiC (2020). HapHiC (Version 1.0) https://github.com/zengxiaofei/HapHiC.
27. Simão FA, Waterhouse RM, Ioannidis P, et al. BUSCO: assessing genome assembly and annotation completeness with single-copy orthologs. Bioinformatics 2015;31(19):3210-2. https://doi.org/10.1093/bioinformatics/btv351.
28. BUSCO (2021). BUSCO (Version 5.3.2) http://busco.ezlab.org/.
29. Rhie A, Walenz BP, Koren S, et al. Merqury: reference-free quality, completeness, and phasing assessment for genome assemblies. Genome Biol 2020;21(1):245. https://doi.org/10.1186/s13059-020-02134-9.
30. Merqury (2020). Merqury (Version 1.3) https://github.com/marbl/merqury.
31. Li H. Minimap2: pairwise alignment for nucleotide sequences. Bioinformatics 2018;34(18):3094-100. https://doi.org/10.1093/bioinformatics/bty191.
32. Minimap2 (2023). Minimap2 (Version 2.26-r1175) https://github.com/lh3/minimap2.
33. Ou S, Su W, Liao Y, et al. Benchmarking transposable element annotation methods for creation of a streamlined, comprehensive pipeline. Genome Biol 2019;201-18. https://doi.org/10.1186/s13059-019-1905-y.
34. EDTA (2022). EDTA (Version 2.0.1) https://github.com/oushujun/EDTA.
35. Gabriel L, Brůna T, Hoff KJ, et al. BRAKER3: Fully automated genome annotation using RNA-seq and protein evidence with GeneMark-ETP, AUGUSTUS, and TSEBRA. Genome Res 2024. https://doi.org/10.1101/gr.278090.123.

36. Bruna T, Lomsadze A,Borodovsky MG-E. A new gene finding tool GeneMark-ETP significantly improves the accuracy of automatic annotation of large eukaryotic genomes. bioRxiv: 2023. https://doi.org/10.1101/2023.01.13.524024
37. Kovaka S, Zimin AV, Pertea GM, et al. Transcriptome assembly from long-read RNA-seq alignments with StringTie2. Genome Biol 2019;201-13. https://doi.org/10.1186/s13059-019-1910-1.
38. Pertea G,Pertea M. GFF Utilities: GffRead and GffCompare. F1000Research 2020, 9, 304. https://doi.org/10.5256/f1000research.25718.r62867.
39. Quinlan AR. BEDTools: the Swiss-army tool for genome feature analysis. Current protocols in bioinformatics 2014;47(1):11.2. 1-.2. 34. https://doi.org/10.1002/0471250953.bi1112s47.
40. BRAKER3 (2023). BRAKER3 (Version 3.0.8) https://github.com/Gaius-Augustus/BRAKER.
41. Pertea M, Kim D, Pertea GM, et al. Transcript-level expression analysis of RNA-seq experiments with HISAT, StringTie and Ballgown. Nat Protoc 2016;11(9):1650-67. https://doi.org/10.1038/nprot.2016.095.
42. Kim D, Paggi JM, Park C, et al. Graph-based genome alignment and genotyping with HISAT2 and HISAT-genotype. Nat Biotechnol 2019;37(8):907-15. https://doi.org/10.1038/s41587-019-0201-4.
43. HISAT (2020). HISAT (Version 2.2.1) http://ccb.jhu.edu/software/hisat2/index.shtml.
44. Jones P, Binns D, Chang H-Y, et al. InterProScan 5: genome-scale protein function classification. Bioinformatics 2014;30(9):1236-40. https://doi.org/10.1093/bioinformatics/btu031.
45. InterProScan (2020). InterProScan (Version 5.45-80.0) https://github.com/ebi-pf-team/interproscan.
46. Camacho C, Coulouris G, Avagyan V, et al. BLAST+: architecture and applications. BMC Bioinformatics 2009;101-9. https://doi.org/10.1186/1471-2105-10-421.
47. Kim D, Langmead B,Salzberg SL. HISAT: a fast spliced aligner with low memory requirements. Nat Methods 2015;12(4):357-60. https://doi.org/10.1038/nmeth.3317.
48. Pertea M, Pertea GM, Antonescu CM, et al. StringTie enables improved reconstruction of a transcriptome from RNA-seq reads. Nat Biotechnol 2015;33(3):290-5. https://doi.org/10.1038/nbt.3122.
49. StringTie (2020). StringTie (Version 2.1.7) https://ccb.jhu.edu/software/stringtie/.
50. Ahdritz G, Bouatta N, Floristean C, et al. OpenFold: Retraining AlphaFold2 yields new insights into its learning mechanisms and capacity for generalization. Nat Methods 2024;1-11. https://doi.org/10.1038/s41592-024-02272-z.
51. OpenFold (2023). OpenFold (Version 2.0) https://github.com/aqlaboratory/openfold.
52. Watvina (2021). Watvina (Version 1.0) https://github.com/biocheming/watvina.
53. Goodsell DS, Morris GM,Olson AJ. Automated docking of flexible ligands: applications of AutoDock. J Mol Recognit 1996;9(1):1-5. https://doi.org/10.1002/(SICI)1099-1352(199601)9:1<1::AID-JMR241>3.0.CO;2-6.
54. Huey R, Morris GM, Olson AJ, et al. A semiempirical free energy force field with charge-based desolvation. J Comput Chem 2007;28(6):1145-52. https://doi.org/10.1002/jcc.20634.
55. AutoDock Tools (2010). AutoDock Tools (Version 1.5.6) http://autodock.scripps.edu/.
56. Páll S, Abraham MJ, Kutzner C, et al. Tackling exascale software challenges in molecular dynamics simulations with GROMACS. In: Solving Software Challenges for Exascale:

International Conference on Exascale Applications and Software, EASC 2014, Stockholm, Sweden, April 2-3, 2014, Revised Selected Papers 2 2015, pp.3-27. Springer. https://doi.org/10.1007/978-3-319-15976-8_1.

57. Abraham MJ, Murtola T, Schulz R, et al. GROMACS: High performance molecular simulations through multi-level parallelism from laptops to supercomputers. SoftwareX 2015;119-25. https://doi.org/10.1016/j.softx.2015.06.001.

58. Gromacs (2023). Gromacs (Version 2023.2) http://www.gromacs.org.

59. Case DA, Cheatham III TE, Darden T, et al. The Amber biomolecular simulation programs. J Comput Chem 2005;26(16):1668-88. https://doi.org/10.1002/jcc.20290.

60. AmberTools (2023). AmberTools (Version 24) https://ambermd.org/AmberTools.php.

61. Sprenger K, Jaeger VW,Pfaendtner J. The general AMBER force field (GAFF) can accurately predict thermodynamic and transport properties of many ionic liquids. The Journal of Physical Chemistry B 2015;119(18):5882-95. https://doi.org/10.1021/acs.jpcb.5b00689.

62. Amber (2020). Amber (Version 20) https://ambermd.org/.

63. Wu J-J, Han Y-W, Lin C-F, et al. Benchmarking gene set of gymnosperms for assessing genome and annotation completeness in BUSCO. Horticulture Research 2023;10(9):uhad165. https://doi.org/10.1093/hr/uhad165.

64. Song C, Fu F, Yang L, et al. *Taxus yunnanensis* genome offers insights into gymnosperm phylogeny and taxol production. Communications Biology 2021;4(1):1203. https://doi.org/10.1038/s42003-021-02697-8.

65. He Z, Luo X, Lei Y, et al. Five Species of Taxus Karyotype Based on Oligo-FISH for 5S rDNA and (AG3T3) 3. Genes 2022;13(12):2209. https://doi.org/10.3390/genes13122209.

66. Fornara F, Panigrahi KC, Gissot L, et al. *Arabidopsis* DOF transcription factors act redundantly to reduce CONSTANS expression and are essential for a photoperiodic flowering response. Dev Cell 2009;17(1):75-86. https://doi.org/10.1016/j.devcel.2009.06.015.

67. Corrales AR, Carrillo L, Lasierra P, et al. Multifaceted role of cycling DOF factor 3 (CDF3) in the regulation of flowering time and abiotic stress responses in *Arabidopsis*. Plant, Cell Environ 2017;40(5):748-64. https://doi.org/10.1111/pce.12894.

68. Lou H, Song L, Li X, et al. The *Torreya grandis* genome illuminates the origin and evolution of gymnosperm-specific sciadonic acid biosynthesis. Nat Commun 2023;14(1):1315. https://doi.org/10.1038/s41467-023-37038-2.

69. Xu R, Yu D, Yang S, et al. Induction and Maintenance of Callus and Paclitaxel Production in *Torreya grandis*. Curr Top Nutraceut R 2019;17(4). https://doi.org/10.37290/ctnr2641-452X.17:363-371.

70. Liu H, Wang X, Wang G, et al. The nearly complete genome of *Ginkgo biloba* illuminates gymnosperm evolution. Nat Plants 2021;7(6):748-56. https://doi.org/10.1038/s41477-021-00933-x.

71. Walas Ł, Mandryk W, Thomas PA, et al. Sexual systems in gymnosperms: a review. Basic Appl Ecol 2018;311-9. https://doi.org/10.1016/j.baae.2018.05.009.

72. González-Martínez SC, Ersoz E, Brown GR, et al. DNA sequence variation and selection of tag single-nucleotide polymorphisms at candidate genes for drought-stress response in *Pinus taeda L*. Genetics 2006;172(3):1915-26. https://doi.org/10.1534/genetics.105.047126.

73. Leslie AB, Beaulieu JM, Crane PR, et al. Explaining the distribution of breeding and dispersal syndromes in conifers. Proc R Soc B 2013;280(1770):20131812. https://doi.org/10.1098/rspb.2013.1812.

74. Ming R, Bendahmane A,Renner SS. Sex chromosomes in land plants. Annu Rev Plant Biol 2011;62485-514. https://doi.org/10.1146/annurev-arplant-042110-103914.

75. Segawa M, Kishi S,Tatuno S. Sex chromosomes of *Cycas revoluta*. The Japanese Journal of Genetics 1971;46(1):33-9. https://doi.org/10.1266/jjg.46.33.

76. Tanaka N, Takemasa N,Sinoto Y. Karyotype Analysis in Gymnospermae, I Karyotype and chromosome bridge in the young leaf meristem of *Ginkgo biloba* L. Cytologia 1952;17(2):112-23. https://doi.org/10.1508/cytologia.17.112.

77. Lee C. Sex chromosomes in *Ginkgo biloba*. Am J Bot 1954;545-9. https://doi.org/10.2307/2438713.

78. Newcomer EH. The karyotype and possible sex chromosomes of *Ginkgo biloba*. Am J Bot 1954;542-5. https://doi.org/10.2307/2438712.

79. Lan T, Chen R, Li X, et al. Microdissection and painting of the W chromosome in *Ginkgo biloba* showed different labelling patterns. Bot Stud 2008;4933-7. https://doi.org/1817406X-200801-49-1-33-37-a.

80. Zhang H, Zhang R, Yang X, et al. Recent origin of an XX/XY sex-determination system in the ancient plant lineage *Ginkgo biloba*. BioRxiv 2019;517946. https://doi.org/10.1101/517946.

81. Liao W, Zhao S, Zhang M, et al. Transcriptome assembly and systematic identification of novel cytochrome P450s in *Taxus chinensis*. Front Plant Sci 2017;81468. https://doi.org/10.3389/fpls.2017.01468.

82. Li C, Zhu Y, Guo X, et al. Transcriptome analysis reveals ginsenosides biosynthetic genes, microRNAs and simple sequence repeats in *Panax ginseng* CA Meyer. BMC Genomics 2013;141-11. https://doi.org/10.1186/1471-2164-14-245.

83. Guo J, Zhou YJ, Hillwig ML, et al. CYP76AH1 catalyzes turnover of miltiradiene in tanshinones biosynthesis and enables heterologous production of ferruginol in yeasts. P Natl A Sci 2013;110(29):12108-13. https://doi.org/10.1073/pnas.1218061110.

84. Li Z. Supporting data for "Phased High-Quality Genome of the Gymnosperm Himalayan Yew Assists in Paclitaxel Pathway Exploration". The NIH Common Fund's National Metabolomics Data Repository (NMDR) website, the Metabolomics Workbench 2025. http://dx.doi.org/10.21228/M8FV76.

85. Li Z; Zong H; Liu X; Wang X; Liu S; Jiao X; Chen X; Wu H; Liu Z; Wang Z; Wang Y; Liu Y; Zhou B; Li Z; Du Q; Li J; Cheng J; Bai J; Zhu X; Yang Y; Liu G; Zhang L; Jiang H; Wang W. Supporting data for "Phased High-Quality Genome of the Gymnosperm Himalayan Yew Assists in Paclitaxel Pathway Exploration" GigaScience Database 2025. https://doi.org/10.5524/102659

Click here to access/download;Table;Tables.docx

Table 1 | Assembly Statistics of Taxus Genus *TWv0*,*TWv1*,*TCv0* and *TYv0* Genomes.

Assembly	TWv0	TWv1	TWv1-hap1	TWv1-hap2	TCv0	TYv0
QV (consensus quality value)	19.3585	59.5658	60.1364	60.7947	\	\
Completeness	52.39%	99.36%	\	\	\	\
Contig N50 (Mb)	8.6	169.4	213.7	136.6	2.44	2.89
Genome size (bp)	11,119,083,473	20,348,291,365	9,868,113,160	9,979,816,178	10,232,176,133	10,737,203,084
gap numbers	8,004	201	85	116	12,092	11,130

Figure Click here to access/download;Figure;Figures.pdf

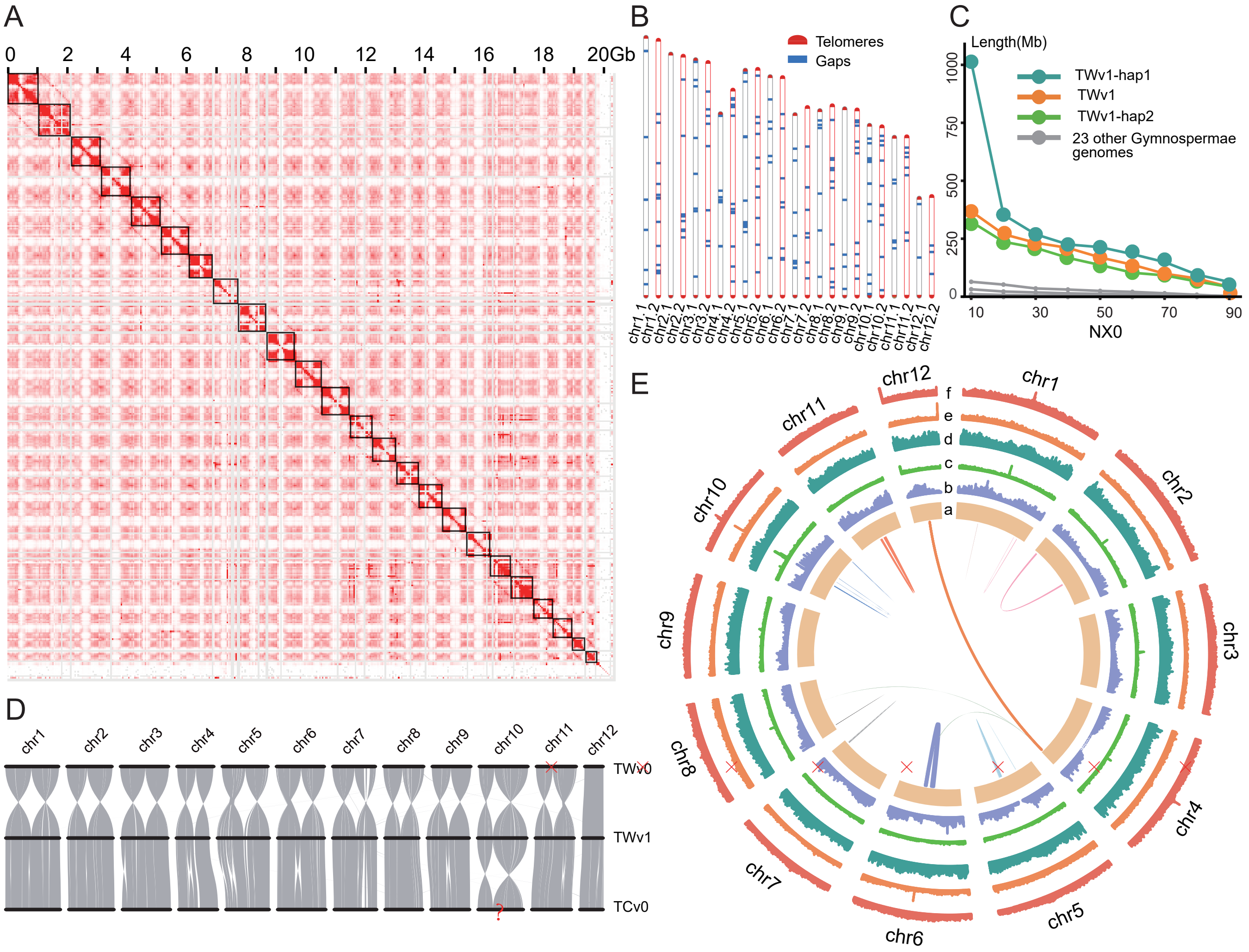

Figure 1. Overview of the Himalayan yew genome TWv1 assembly.

(A) Hi-C chromatin interaction heatmap of the TWv1 assembly. Each black box represents a single chromosome. Chromosome numbers correspond to those in Figure D.(B) Distribution of gaps and telomere sequences across the 12 chromosomes in the TWv1-hap1 genome. Chromosome 2 is assembled to telomere-to-telomere (T2T) completeness. (C) Comparison of NX0 (N10-N90) between the Himalayan yew and 23 other gymnosperm genomes. (D) Collinearity comparison of different versions of the yew reference genomes. Red crosses indicate 11 intrachromosomal assembly errors present in TWv0, while red question marks denote a potential "inversion" assembly error in the TCv0 chromosome. (E) TWv1 Circos graph. From the innermost to the outermost circle (a-f) are: a) chromosome length, b) gene number distribution, c) GC content, d) LTR/Gypsy distribution, e) LTR/Copia distribution, f) DNA transposon distribution. The lines in the center of the circle indicate collinear regions.

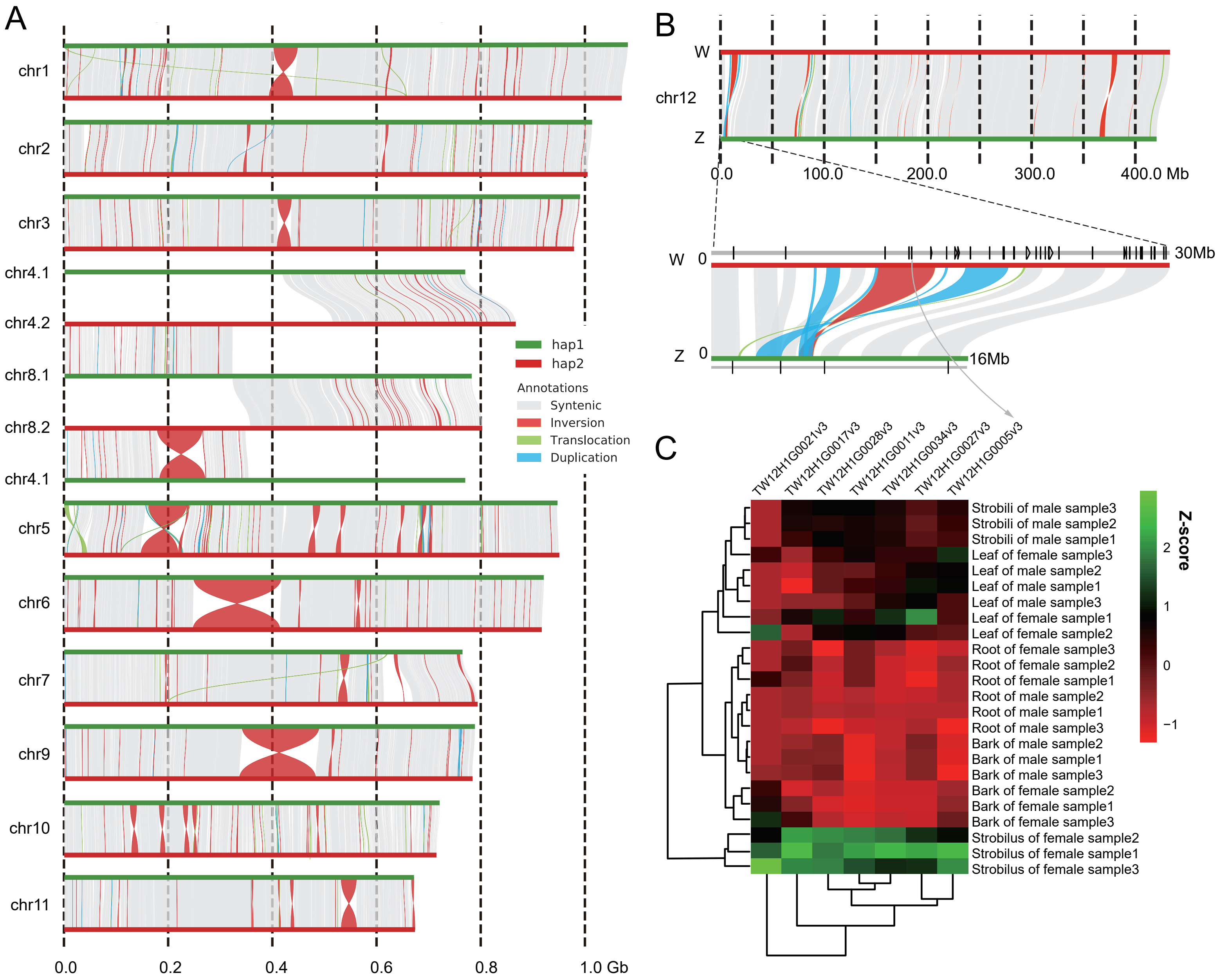

Figure 2. Structural variations in TWv1.
(A) Structural variations between the two haplotypes of chr1-chr11. Gray lines represent collinear regions, red lines represent insertions, green lines represent translocations, and blue lines represent duplications. (B) Structural variations and non-homologous regions between the Z/W haplotypes of the sex chromosome chr12. (C) Heatmap of gene expression differences in the non-homologous regions of the Z/W chromosomes.

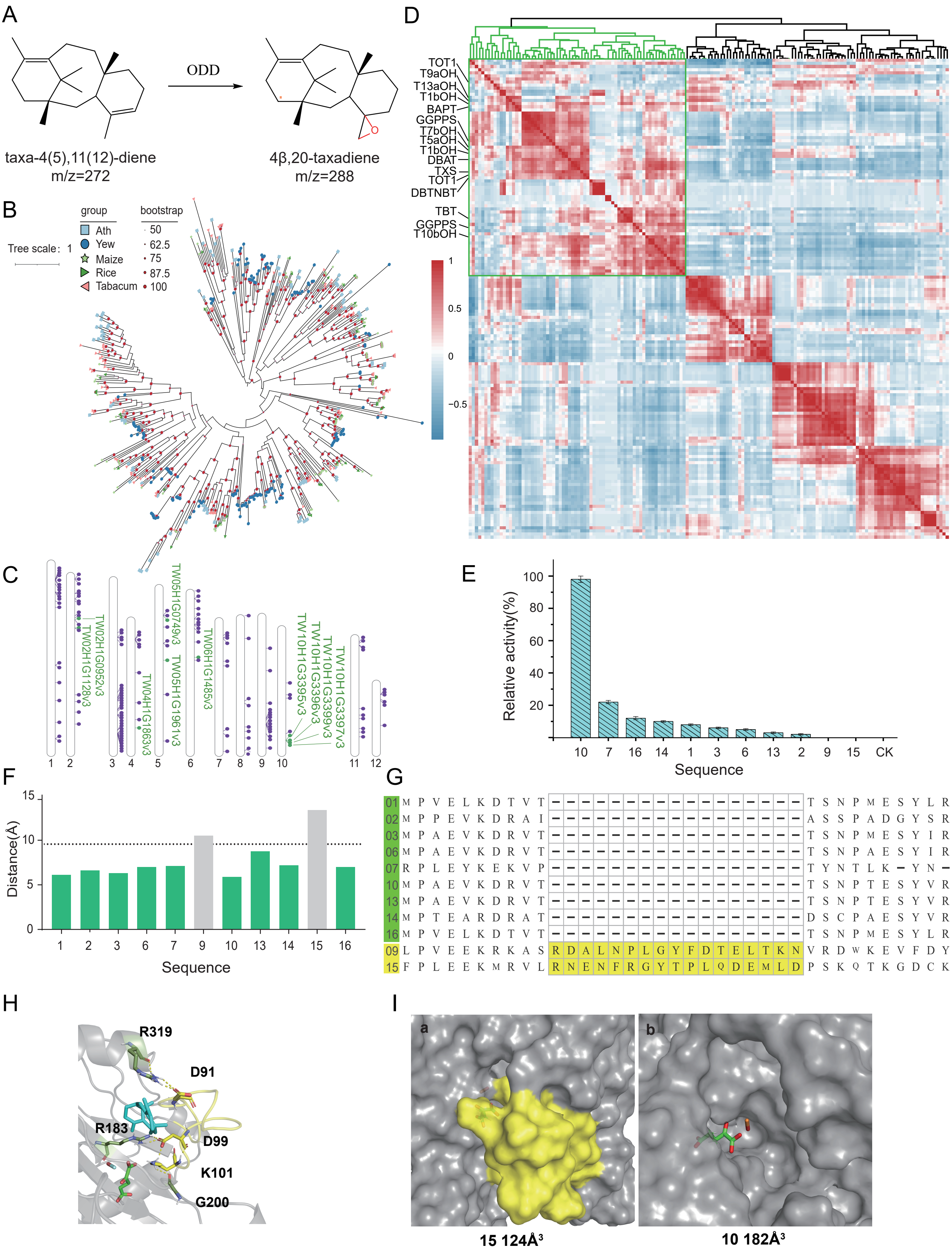
A
ODD
taxa-4(5),11(12)-diene
m/z=272
4β,20-taxadiene
m/z=288
B
group
bootstrap
Ath
Yew
Maize
Rice
Tabacum
50
62.5
75
87.5
100
Tree scale: 1
C
TW02H1G0952v3
TW02H1G1128v3
TW04H1G1863v3
TW05H1G0749v3
TW05H1G1961v3
TW06H1G1485v3
TW10H1G3395v3
TW10H1G3396v3
TW10H1G3399v3
TW10H1G3397v3
1 2 3 4 5 6 7 8 9 10 11 12
D
TOT1
T9aOH
T13aOH
T1bOH
BAPT
GGPPS
T7bOH
T5aOH
T1bOH
DBAT
TXS
TOT1
DBTNBT
TBT
GGPPS
T10bOH
1
0.5
0
−0.5
E
Relative activity(%)
100
80
60
40
20
10 7 16 14 1 3 6 13 2 9 15 CK
Sequence
F
Distance(Å)
15
10
5
0
1 2 3 6 7 9 10 13 14 15 16
Sequence
G
01 M P V E L K D T V T T S N P M E S Y L R
02 M P P E V K D R A I A S S P A D G Y S R
03 M P A E V K D R V T T S N P M E S Y I R
06 M P A E V K D R V T T S N P A E S Y I R
07 R P L E Y K E K V P T Y N T L K − Y N −
10 M P A E V K D R V T T S N P T E S Y V R
13 M P A E V K D R V T T S N P T E S Y V R
14 M P T E A R D R A T D S C P A E S Y V R
16 M P V E L K D T V T T S N P M E S Y L R
09 L P V E E K R K A S R D A L N P L G Y F D T E L T K N V R D W K E V F D Y
15 F P L E E K M R V L R N E N F R G Y T P L Q D E M L D P S K Q T K G D C K
H
R319
D91
R183
D99
K101
G200
I
a
b
15 124Å³
10 182Å³

Figure 3. Discovery and activity analysis of ODD enzymes.

(A) Predicted epoxidation function of ODD. (B) ODD gene family tree (showing the distribution and evolutionary relationships of the identified genes). (C) Chromosomal distribution of paclitaxel synthesis-related ODD enzymes. Purple circles indicate all ODDs in TWv1, and green circles indicate candidate genes screened by co-expression analysis. (D) Heatmap of co-expression of genes related to the paclitaxel synthesis pathway. (E) Relative activity assay of the epoxidation function of the 11 candidate genes.
(F) Comparison of catalytic distance and activity of the 11 candidate genes. Green indicates catalytic activity; gray indicates no catalytic activity. (G) Multiple sequence alignment analysis of the 11 candidate genes. (H) Analysis of hydrogen bond formation in loop 86-101 of the catalytic pocket of sequence 15.
(I) a) Catalytic pocket of sequence 15, b) catalytic pocket of sequence 10.

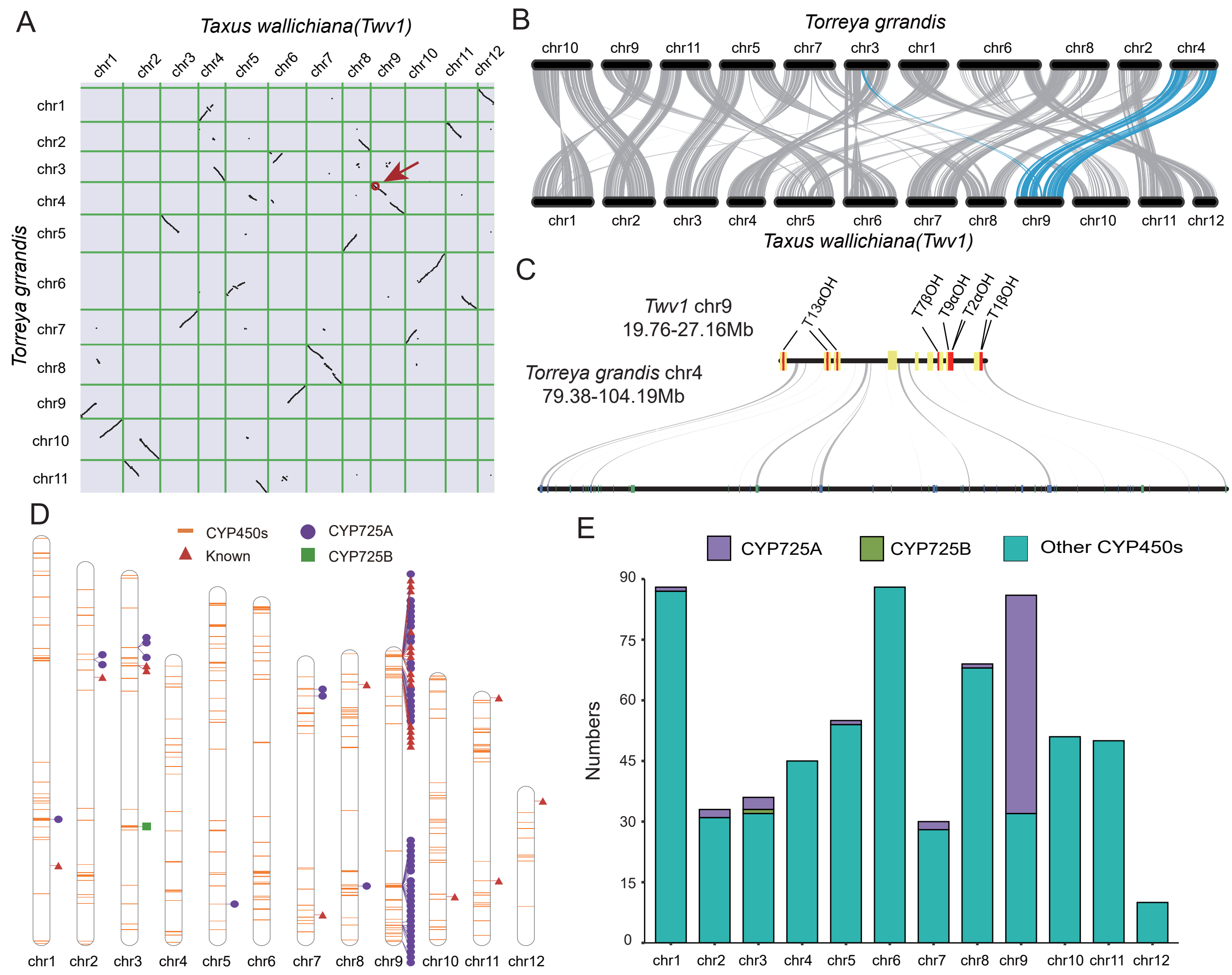

Figure 4. Collinearity and P450 cluster analysis of TWv1.

(A) Chromosomal collinearity between TWv1 and *Torreya grandis*. Collinear regions between TWv1 and *Torreya grandis* are indicated by black dots. The red arrows indicate the locations of the paclitaxel biosynthesis gene clusters. (B) Collinear graph between TWv1 and *Torreya grandis*. Gray lines represent collinear regions between TWv1 and *Torreya grandis*, and blue lines represent collinear regions between chr9 of TWv1 and *Torreya grandis*. (C) Collinear region of the gene cluster on chr9 of TWv1 and chr4 of *Torreya grandis*. Yellow lines on chr9 of TWv1 indicate enzymes belonging to the CYP725A subfamily, and red lines indicate enzymes involved in paclitaxel biosynthesis. (D) Distribution of P450 gene clusters across the 12 chromosomes. Orange lines indicate all P450 enzymes in TWv1, red triangles indicate characterized enzymes, purple circles indicate enzymes belonging to the CYP725A subfamily, and green squares indicate enzymes belonging to the CYP725B subfamily. (E) Histogram of P450 numbers across the 12 chromosomes. Purple bars represent enzymes belonging to the CYP725A subfamily, green bars represent enzymes belonging to the CYP725B subfamily, and blue bars represent all other CYP450 enzymes outside the CYP725 family.

Click here to access/download
Supplementary Material
Supplementary Material.docx
