# Supplementary material for: Phased high-quality genome of the gymnosperm Himalayan Yew assists in paclitaxel pathway exploration
Source: Gigascience. 2025 Apr 4;14:giaf026. doi: 10.1093/gigascience/giaf026 (PMC11970372; doi:10.1093/gigascience/giaf026)
Supplement: giaf026_Response_to_Reviewer_Comments_Original_Submission [file giaf026_response_to_reviewer_comments_original_submission.pdf]

Author's Response To Reviewer Comments

Dear Editors and Reviewers,

Thank you very much for handling our manuscript entitled "Phased High-Quality Genome of the Gymnosperm Himalayan Yew Assists in Paclitaxel Pathway Exploration" (Manuscript_GIGA-D-24-00293) for consideration for publication in Giga science as a research article. We thank the reviewers and you for providing highly valuable suggestions and comments

The constructive suggestions and comments have helped us to significantly improve the manuscript. Point-by-point responses addressing all the questions raised by the reviewers are included in the response letter. Here we report to you the major changes in the revised version of the manuscript.
1) In this revision, we reassembled the genome of Taxus wallichiana using the newest pipelines as the reviewers suggested. We first utilized hifiasm to integrate three types of data: HiFi, Hi-C, and ONT UL, completing the assembly of two haplotype genomes. Subsequently, we used HapHiC to anchor the contigs onto 24 chromosomes and manually checked potential assembly errors, including switch errors. Finally, the total number of gaps across the entire genome was reduced to only 201, with chr2 in haplotype 1 achieving T2T-level assembly. Merqury evaluation showed a completeness of 99.36%, a QV of 59, and a contig N50 of 169.4 Mb, indicating the assembled genome in this revision is now a phased high-quality genome.

2) In this revision, we calculated the length differences between each pair of homologous chromosomes. Among them, chr12 showed the largest proportional length difference (1.7%), and we designated chr12 as the candidate sex chromosome. Based on this, we identified a Dof zinc finger protein family gene in the highly variable regions of the two homologous chromosomes. This gene is involved in flowering control in Arabidopsis thaliana. Nevertheless, our results only provide clues that this pair of chromosomes may be the sex chromosome, and we thus toned down the related sentences.

3) In this revision, we used BRAKER3 to annotate the genome by incorporating protein sequences from 20 gymnosperm species and approximately 300 Gb of transcriptome data. The protein dataset used for annotation was assessed with embryophyta_odb10, achieving a completeness of 99.1%. The final annotation results were evaluated using embryophyta_odb10, with completeness scores of 95.4%, 92.7%, and 92.9% for the whole genome, hap1, and hap2, respectively. When evaluated with Gymnosperm_odb10, the completeness scores were 99.7%, 97.4%, and 97.9% for the whole genome, hap1, and hap2, respectively. The transcriptome data covered approximately 85% of the annotation results. Functional annotation was performed using Swiss-Prot, TrEMBL, NR, KEGG, and GO databases, with more than 85% of the proteins successfully annotated. Therefore, the genome annotation methods and results in this revision are reliable.

4) we evaluated the completeness of our genome and annotations using embryophyta_odb10, and both the assessments exceeded 90%, indicating both the assembled genome and gene annotations are of high quality.

5) We have updated the corresponding methods. We also carefully checked all the figures and tables for references to make sure citing the correct papers.

6) We have updated all the required data and re-uploaded it via the FTP client. The reviewer link for the data uploaded to NCBI is provided here for your reference:
https://dataview.ncbi.nlm.nih.gov/object/PRJNA1146068?reviewer=p1cr7uuq6b7lde22m0os3or5os.

In addition, we added five more authors: Zhuoya Liu, Zhongkai Wang, Yongqiang Wang, Botong Zhou and Zihe Li, because of their significant contributions to the data analysis and statistics in the revised manuscript.
We hope you will see all the revisions and new results have fully addressed all the questions and concerns raised by the reviewers.

Thank you again for your rigorous and helpful efforts in handling our manuscript. If further information is needed, please feel free to let us know.

Yours sincerely,

Wen Wang PhD.
School of Ecology and Environment, Northwestern Polytechnical University, Xi'an 710072, China;
Phone: +86 18629418666;
Email: wenwang@nwpu.edu.cn

Huifeng Jiang PhD.
Key Laboratory of Systems Microbial Biotechnology, Tianjin Institute of Industrial Biotechnology, Chinese Academy of Sciences, Tianjin 300308, China.
Phone: +86 13622110805;
Email: jiang_hf@tib.cas.cn

Response to Reviewer

We would like to thank the reviewers for the helpful comments, all of which have significantly helped to improve this manuscript. Please find our point-by-point responses to the comments as follows.

Reviewer #1:
Thank you very much for your review and comments. We have modified the manuscript according to your suggestions, and our responses are as follows:

1. Li and colleagues successfully assembled a high-quality diploid genome of Taxus wallichiana and conducted data mining for ODDs, key C4β-C20 epoxidases in the paclitaxel synthesis pathway. Overall, this work is solid and engaging, and the data will serve as a valuable resource for future research in the field. However, several concerns about the data are outlined below.

The logic behind haplotype phasing in this study is difficult to follow. The authors used UL reads and hap2 to fill the gaps in hap1, leaving hap1 unresolved, yet they still claim this represents a resolved diploid genome. In my opinion, the most effective approach to achieving a high-quality genome would be to close the gaps in the primary contig assembly, which provides consensus sequences for both haplotypes.

Response:
Thank you for your valuable suggestions. Following your suggestion, we did not use the hap 2 to fill the hap1, and reassembled the genome of Taxus wallichiana using the newest assembly pipeline. We first utilized hifiasm to integrate three types of data: HiFi, Hi-C, and ONT UL, completing the assembly of two haplotype genomes. Subsequently, we used HapHiC to anchor the contigs onto 24 chromosomes and manually checked potential assembly errors, including switch errors. Finally, the total number of gaps across the entire genome was reduced to only 201, with chr2 in haplotype 1 achieving T2T-level assembly. Merqury evaluation showed a completeness of 99.36%, a QV of 59, and a contig N50 of 169.4 Mb, indicating the assembled genome in this revision is now a phased high-quality genome.
The relevant content has been modified in the main text, please see Fig. 1A and Table 1. For the convenience of the reviewer, we have pasted the details below:
Line 141-158:
"Genome assembly and quality assessment: Using the hifiasm software (Hifiasm 0.19.8-r602, https://github.com/chhylp123/hifiasm, RRID:SCR_021069) [21], we integrated ONT UL sequencing data, HiFi sequencing data and Hi-C sequencing data to perform the phased assembly of the T. wallichiana genome (parameter: hifiasm -l 0). Using BWA software (BWA 0.7.17-r1198-dirty, http://bio-bwa.sourceforge.net/, RRID:SCR_010910) [22], we aligned the Hi-C data to the genome which is merged by two haplotype assemblies. The HapHiC pipeline (HapHiC, https://github.com/zengxiaofei/HapHiC) [23]

was then employed to scaffold the contigs to the chromosome level, followed by manual correction of any assembly errors. The completeness of the genome was assessed using BUSCO (BUSCO 5.3.2, http://busco.ezlab.org/, RRID:SCR_015008) [24]. Merqury software (Merqury 1.3, https://github.com/marbl/merqury, RRID:SCR_022964) [25] was used to evaluate the genome's completeness and the error rate of each chromosome and the overall error rate using HiFi reads. HiFi reads and ONT UL reads were aligned to the reference genome using minimap2 software (Minimap2 2.26-r1175, https://github.com/lh3/minimap2, RRID:SCR_018550) [26] to assess depth distribution and to evaluate haplotype depth distribution based on HiFi reads.”.
Line 262-271:
“Utilizing the Hi-C data, we anchored the contigs of both haplotypes onto 24 chromosomes, achieving an anchoring rate of 97.5%. The continuity of the Hi-C heatmap indicates that the genome has no large-scale assembly errors and that the two haplotypes were correctly phased (Fig. 1A, Table 1). As a result, we obtained a high-quality haplotype-resolved genome, TWv1, with a total genome size of approximately 20.3 Gb, consisting of 24 chromosomes and a contig N50 length of 169.4 Mb (Table 1). Each chromosome contains complete telomeres at both ends, and the TWv1 genome includes only 201 gaps (Fig1B, Supplementary Table S5). The genome sizes of haplotype 1 (TWv1-hap1) and haplotype 2 (TWv1-hap2) are 9.87 Gb and 9.98 Gb, respectively.”.

2. I was surprised to see a large chunk of genome sequences translocated between chromosomes in the two haplotypes (Fig. S2). Given the extremely high percentage of TEs in the genome, the possibility of mis-assembly cannot be ruled out based on the current data. It would be important to verify the coverage at the junction sites using PacBio reads to determine if the read coverage is continuous or interrupted.

Response:
Thank you for your valuable suggestions. Since the translocation boundaries between chr4 and chr8 occur at the junctions between contigs, it was challenging for us to verify this using the depth distribution of HiFi or ONT UL reads, as you suggested. Instead, we used strictly filtered Hi-C reads (excluding supplementary alignment reads) to verify the translocation. The strong intra-chromosomal signals provide evidence for the correctness of our scaffold assembly, which also supports the authenticity of the translocation between chr4 and chr8. We have updated the relevant content in the main text. The corresponding results are shown in Supplementary Figure 1, and for your convenience, we also past them below:
Line 327-335:
“To investigate the genetic polymorphism of Himalayan yew, we identified structural variations (length > 50 bp) between the two haplotype genomes in TWv1 (Fig. 2). The analysis revealed that the total length of all structural variations accounts for 13.4% of the entire genome, including 64 duplications, 66 translocations, and 267 inversions (Supplementary Tables S11, S12). We found four ultra-large structural variations greater than 100 Mb in length between the two haplotypes, including a 366 Mb interchromosomal translocation between chr4 and chr8, and inversions of 169 Mb and 148 Mb on chr6 and chr9, respectively (Fig. 2A, Supplementary Fig. S1, Supplementary Table S13).”.

Figure S1. Collinear Features of TWv1. (A) Collinearity between TWv1-hap1 and TWv1-hap2. Gray lines represent Collinear regions between chromosomes, orange lines indicate translocations of chromosomal segments, and green lines indicate inversions of chromosomal segments. (B) Hi-C heatmap evidence of Collinearity within the TWv1 genome.

3. Many chromosomes display length variations between haplotypes, so why do the authors believe that Chr12 is the sex chromosome?

Response:
Thank you for pointing out this problem, which has intrigued us to finely tackle this question and the related statements are toned down. In this revision, we calculated the length differences between each pair of homologous chromosomes. Among them, chr12 showed the largest proportional length difference (1.7%), and we designated chr12 as the candidate sex chromosome. Based on this, we identified a Dof zinc finger protein family gene in the highly variable regions between the two homologous chromosomes. This gene is involved in flowering control in Arabidopsis thaliana. Nevertheless, our results only provide clues that this pair of chromosomes may be the sex chromosome, and we thus toned down the related sentences, which are pasted below:

Line 337-361:
“To avoid misjudgment of chromosome length due to assembly errors, we evaluated the collapsed regions of the genome based on the mapping depth of HiFi reads, using a 100 Kb window (Supplementary Table S14). Statistics indicate that a total of 218 Mb of regions in the genome exhibit collapse, and they only distribute in chr4, chr7, chr8, with a collapse rate of 1.07% (Supplementary Fig. S2). The total length of collapsed sequences in TWv1-hap1 is 42 Mb, with a collapse rate of only 0.42%. By summing the collapsed lengths and assembled lengths of each chromosome, we predicted the actual lengths of Himalayan yew chromosomes. Comparisons of lengths between all pairs of homologous chromosomes show that the length differences between haplotypes range from 0.12% to 1.71%. Aside from the chromosomal translocations observed in chr4 and chr8, the largest length difference is found in chr12, with a sequence length difference of 7.2 Mb, accounting for 1.71% and 1.68% of the chr12 of two haplotypes, respectively. This length difference is caused by the 0-28 Mb region of chr12.2 and the 0-16 Mb region of chr12.1 (Fig. 2B, Supplementary Table S14). According to previous karyotype studies [42], this pair of chromosomes also exhibits length differences, and was considered as the candidate sex chromosomes. Given that the individual assembled in this study is female as indicated by its yield of seeds (Supplementary Fig. S3), this result suggests that the sex determination in Himalayan yew might follow the ZW model, with chr12.1 and chr12.2 representing the candidate Z and W chromosomes, respectively (Fig. 2B). The identification of a Dof zinc finger protein family gene, which has been found being involved in flowering control [43,44], in the highly variable regions between the two homologous chromosomes further suggests they may the sex chromosomes. However, more direct evidence is needed to validate this hypothesis.”.

4. The results section includes several methodological details that should be relocated to the methods section or removed if already described.

Response:
The descriptions related to methodological details have been removed from the main text and included in the Methods section accordingly, as pasted below:
Line 416-421:
“To investigate the functional roles of the selected ODD genes in the paclitaxel biosynthesis pathway, we introduced 11 candidate genes into the yeast cytosolic taxadiene production chassis we previously constructed [4]. GC-MS activity validation results showed that 9 out of the 11 genes exhibited potential C4-C20β epoxidation activity (Fig. 3E, Supplementary Fig. S4), while sequence 9 and sequence 15 did not display any catalytic activity.”.

5. Line 60-61: This statement is inaccurate, as gap regions are primarily composed of repetitive sequences, which often lack functional genes.

Response:
Thank you for your suggestion. The relevant descriptions have been adjusted per your recommendation (Line 55-62):
“Previously, three haploid genomes of the genus Taxus have been published [4-6], including Taxus wallichiana (T. wallichiana) from our group [4]. However, these genomes were assembled using either second-generation sequencing or third-generation sequencing technologies with relatively high error rates, resulting in a large number of unfilled gaps (11,130, 8,004, and 12,092 gaps, respectively) and many assembly errors. These assembly issues and unphased assembly pose challenges for the identification of key metabolite biosynthetic enzymes and sex chromosomes.”.

6. Line 121: It is recommended to specify whether the sequenced tree was male or female.

Response:
Thank you for your reminder. This is a female tree. We have added the description in Line 119-121:
“To investigate the genome of Taxus wallichiana, fresh leaves were collected from a female Himalayan yew tree, estimated to be at least 50 years old, cultivated at the Kunming Institute of Botany, Chinese Academy of Sciences.”.

7. Line 433: Cell lines?

Response:
Sorry for the ambiguity. Here "Cell lines" refers to sample names from the transcriptome database. To make this clearer, we have revised it as:
"We downloaded a publicly available dataset containing 40 transcriptome samples, including different cell lines with high and low paclitaxel yields, as well as five tissue types.". (Line 179-181)

Reviewer #2
Thank you very much for your review and comments. We have modified the manuscript according to your suggestions, and our responses are as follows:

1. Assembly quality: The authors used hifiasm assembler to perform the initial assembly of the genome without integrating the available Hi-C data. This is not the correct use of the software to perform haplotype phased assembly. Phasing post-assembly with 3D-DNA pipeline is not the correct way to go, because assembled contigs may already have phasing errors. Also filling gaps in one haplotype with sequence from another haplotype defeats the purpose of phased assembly and introduces phasing errors. The correct way to assemble the data is to use -h1 and -h2 options to supply the HiC data concurrently with the PacBio HiFi data for proper haplotype resolution within hifiasm. This results in correct haplotype resolution and prevents phasing errors. The phased assemblies can be then scaffolded with 3D-DNA or similar pipeline separately. THis makes me doubt the conclusions on large scale structiural variation between the haplotypes -- the varioation could simply be due to phasing errors.

Response:
Thank you very much for pointing this problem. Following your suggestions, we reassembled the genome of Taxus wallichiana. In the initial assembly, we utilized hifiasm to integrate three types of data: HiFi, Hi-C, and ONT UL, completing the assembly of two haplotype genomes. Then we used HapHiC to anchor the contigs onto 24 chromosomes and carefully corrected potential assembly errors, including switch errors. Finally, the total number of gaps across the entire genome was reduced to only 201, with chr2 in haplotype 1 achieving T2T-level assembly. Merqury evaluation showed a completeness of 99.36%, a QV of 59, and a contig N50 of 169.4 Mb, indicating the assembled genome in this revision is now a phased high-quality genome. The relevant content has been modified in the main text, please see Fig. 1A and Table 1. For the convenience of the reviewer, we have pasted the details below:
Line 141-158:
"Genome assembly and quality assessment: Using the hifiasm software (Hifiasm 0.19.8-r602, https://github.com/chhylp123/hifiasm, RRID:SCR_021069) [21], we integrated ONT UL sequencing data, HiFi sequencing data and Hi-C sequencing data to perform the phased assembly of the T. wallichiana genome (parameter: hifiasm -l 0). Using BWA software (BWA 0.7.17-r1198-dirty, http://bio-bwa.sourceforge.net/, RRID:SCR_010910) [22], we aligned the Hi-C data to the genome which is merged by two haplotype assemblies. The HapHiC pipeline (HapHiC, https://github.com/zengxiaofei/HapHiC) [23]was then employed to scaffold the contigs to the chromosome level, followed by manual correction of any assembly errors. The completeness of the genome was assessed using BUSCO (BUSCO 5.3.2, http://busco.ezlab.org/, RRID:SCR_015008) [24]. Merqury software (Merqury 1.3, https://github.com/marbl/merqury, RRID:SCR_022964) [25] was used to evaluate the genome's completeness and the error rate of each chromosome and the overall error rate using HiFi reads. HiFi reads and ONT UL reads were aligned to the reference genome using minimap2 software (Minimap2 2.26-r1175, https://github.com/lh3/minimap2, RRID:SCR_018550) [26] to assess depth distribution and to evaluate haplotype depth distribution based on HiFi reads.".
Line 262-271:
"Utilizing the Hi-C data, we anchored the contigs of both haplotypes onto 24 chromosomes, achieving an anchoring rate of 97.5%. The continuity of the Hi-C heatmap indicates that the genome has no large-scale assembly errors and that the two haplotypes were correctly phased (Fig. 1A, Table 1). As a result, we obtained a high-quality haplotype-resolved genome, TWv1, with a total genome size of approximately 20.3 Gb, consisting of 24 chromosomes and a contig N50 length of 169.4 Mb (Table 1). Each chromosome contains complete telomeres at both ends, and the TWv1 genome includes only 201 gaps (Fig1B, Supplementary Table S5). The genome sizes of haplotype 1 (TWv1-hap1) and haplotype 2 (TWv1-hap2) are 9.87 Gb and 9.98 Gb, respectively.".

Line 327-335:
"To investigate the genetic polymorphism of Himalayan yew, we identified structural variations (length > 50 bp) between the two haplotype genomes in TWv1 (Fig. 2). The analysis revealed that the total length of all structural variations accounts for 13.4% of the entire genome, including 64 duplications, 66 translocations, and 267 inversions (Supplementary Tables S11, S12). We found four ultra-large structural variations greater than 100 Mb in length between the two haplotypes, including a 366 Mb interchromosomal translocation between chr4 and chr8, and inversions of 169 Mb and 148 Mb on chr6 and chr9, respectively (Fig. 2A, Supplementary Fig. S1, Supplementary Table S13).".

Figure S1. Collinear Features of TWv1. (A) Collinearity between TWv1-hap1 and TWv1-hap2. Gray lines represent Collinear regions between chromosomes, orange lines indicate translocations of chromosomal segments, and green lines indicate inversions of chromosomal segments. (B) Hi-C heatmap evidence of Collinearity within the TWv1 genome.

2. Annotation quality: Annotation is performed by an ad hoc approach, combining BRAKER2 annotations with Genewise predictions and Stringtie transcript assemblies with CDSs predicted by TransDecoder. The results were combined with EvidenceModeler. The first problem with this approach is that TransDecoder performs poorly on identifying correct ORFs in assembled transcripts without additional extrinsic evidence, such as protein homology. The second problem is that by combining various software packages, the authors put together a custom annotation pipeline. No evidence is provided as to the performance of this custom pipeline. BRAKER3 software has been available for over a year now, and it performs better than BRAKER2.

Response:
Thank you very much for these helpful suggestions. In this revision, we used BRAKER3 to annotate the genome by incorporating protein sequences from 20 gymnosperm species and approximately 300 Gb of transcriptome data. The protein dataset used for annotation was assessed with embryophyta_odb10, achieving a completeness of 99.1%. The final annotation results were evaluated using embryophyta_odb10, with completeness scores of 95.4%, 92.7%, and 92.9% for the whole genome, hap1, and hap2, respectively. When evaluated with Gymnosperm_odb10, the completeness scores were 99.7%, 97.4%, and 97.9% for the whole genome, hap1, and hap2, respectively. The transcriptome data covered approximately 85% of the annotation results. Functional annotation was performed using Swiss-Prot, TrEMBL, NR, KEGG, and GO databases, with more than 85% of the proteins successfully annotated. We have updated the related descriptions as follows
"Genome annotation: We used EDTA (EDTA v2.0.1, https://github.com/oushujun/EDTA, RRID:SCR_022063) [27] to construct a high-quality, non-redundant repeat sequence library. Gene prediction was performed by BRAKER3 (version 3.0.8, https://github.com/Gaius-Augustus/BRAKER, RRID:SCR_018964) [28-31] using the soft-masked genome. Annotation was performed using protein sequences from 20 gymnosperms and about 300 Gb of transcriptome data (Supplementary Table S1).Transcriptome sequence alignment and assembly were performed using HISAT (HISAT 2.2.1, http://ccb.jhu.edu/software/hisat2/index.shtml, RRID:SCR_015530) [33, 34]. The annotation results were evaluated using BUSCO (BUSCO 5.3.2, http://busco.ezlab.org/, RRID:SCR_015008) [24] with the Gymnosperm_odb10 and embryophyta_odb10 datasets. All protein-coding genes were retrieved and functionally annotated by blast searches against databases including UniProtKB/Swiss-Prot, UniProtKB/TrEMBL, NR, KEGG. They were also subjected to GO annotation and protein family annotation by InterProScan (version 5.45-80.0, https://github.com/ebi-pf-team/interproscan, RRID:SCR_005829) [35]." (Line 160-175).

Line 272-283:
"Multiple approaches were employed to assess the completeness and continuity of the TWv1 genome. For completeness assessment using K-mer based method performed by Merqury software (Merqury 1.3, https://github.com/marbl/merqury, RRID:SCR_022964) [25] revealed a genome quality value (QV) of approximately 59.6, with QV values for individual chromosomes ranging from 57.22 to 64.06 (Table 1; Supplementary Table S6). The overall completeness is 99.36%, with a base error rate as low as 1.00E-06 (Table 1). For the BUSCO assessment of genome completeness, both haplotypes achieved over 95% completeness in the Gymnosperm_odb10 dataset, while in the Embryophyta_odb10 dataset, both

haplotypes reached approximately 90%. This difference may be attributed to the lower suitability of the Embryophyta_odb10 dataset for gymnosperms (Supplementary Table S7) [40].".

3. BUSCO analysis using Gymnosperm database is circular. The single-copy orthologs used in the database have been derived from mainly ab initio gene predictions on previously assembled gymnosperm genomes. These genomes have generally been assembled from short-read data and thus these annotations contain many errors. I suggest using more general BUSCO database of orthologs, such as the plantae database.

Response:
Thank you for this great suggestion. Following your suggestion, in this revision we evaluated the completeness of our genome and annotations using embryophyta_odb10 The resulted evaluation parameters are higher than those reported for published gymnosperms, and thus the assembled genome and gene annotations is of high quality. We have updated the related descriptions as follows
"Multiple approaches were employed to assess the completeness and continuity of the TWv1 genome. For completeness assessment using K-mer based method performed by Merqury software (Merqury 1.3, https://github.com/marbl/merqury, RRID:SCR_022964) [25] revealed a genome quality value (QV) of approximately 59.6, with QV values for individual chromosomes ranging from 57.22 to 64.06 (Table 1; Supplementary Table S6). The overall completeness is 99.36%, with a base error rate as low as 1.00E-06 (Table 1). For the BUSCO assessment of genome completeness, both haplotypes achieved over 95% completeness in the Gymnosperm_odb10 dataset, while in the Embryophyta_odb10 dataset, both haplotypes reached approximately 90%. This difference may be attributed to the lower suitability of the Embryophyta_odb10 dataset for gymnosperms (Supplementary Table S7)[40]." (Line 272-283).
