# Supplementary material for: Phased high-quality genome of the gymnosperm Himalayan Yew assists in paclitaxel pathway exploration
Source: Gigascience. 2025 Apr 4;14:giaf026. doi: 10.1093/gigascience/giaf026 (PMC11970372; doi:10.1093/gigascience/giaf026)
Supplement: giaf026_Response_to_Reviewer_Comments_Revision_1 [file giaf026_response_to_reviewer_comments_revision_1.pdf]

Author's Response To Reviewer Comments

Dear Hongling,

Thank you very much for handling our manuscript entitled "Phased High-Quality Genome of the Gymnosperm Himalayan Yew Assists in Paclitaxel Pathway Exploration" (Manuscript_GIGA-D-24-00293R1) and accepting it in principle to publish in GigaScience as a research article. We thank the reviewers and you for providing valuable suggestions and comments.

The constructive suggestions and comments have helped us to improve the manuscript. Particularly, we follow your suggestions to improve the documentation of all software used, including providing references, identifiers, links, and RRIDs. Additionally, we have cited the DOI for all computational workflows utilized at the relevant locations in the manuscript. In addition, based on the suggestions from Reviewer 2, we have updated the content in Table 1 and revised the corresponding descriptions in the manuscript.
Thank you again for handling our manuscript. If further information is needed, please feel free to let me know.
Yours sincerely,

Wen

Wen Wang PhD.
School of Ecology and Environment, Northwestern Polytechnical University, Xi’an 710072, China;
Phone: +86 18629418666;
Email: wenwang@nwpu.edu.cn

Response to Reviewer

We would like to thank the reviewers for the helpful comments, all of which have helped to improve this manuscript. Please find our point-by-point responses to the comments as follows.

Reviewer #1:
Thank you very much for your review and positive feedback on our work.

Reviewer #2
Thank you very much for your review and comments. We have modified the manuscript according to your suggestions, and our responses are as follows:

1. In Table 1, the number for the genome size for TWv1-hap2 is missing comma separators. Also, "Error rate" and "QV" are the same thing, just represented differently. I would recommend getting rid of the "Error rate" line or replacing it with "Errors per 1Mbp of sequence", as it may be easier to parse for the readers.

Response:
Thank you very much for your suggestions. Following your suggestions, we have added comma separators to the relevant genome size data in Table 1. Additionally, we have removed the "Error rate" row from Table 1 and described the Errors per Mb at Line 286-287: “The overall completeness is 99.36%, which corresponds to approximately one base error per 1 Mbp (Table 1).”.

2. I also would like to see the numbers listed for completeness evaluations by Merqury for the two haplotypes (TWv1-hap1 and TWv1-hap2) in Table 1.

Response:
Thank you for noting this point. The completeness evaluated by Merqury may not be applicable for haplotype genomes. because the metric is calculated as: (Number of K-mer types in the genome) /

(Number of K-mer types in the NGS reads). Therefore, when calculated for the entire genome (TWv1-hap1 + TWv1-hap2), the metric reflects the overall genome quality. However, for haplotype genomes, the denominator remains the "number of K-mer types in the NGS reads," which cannot barely be distinguished between haplotypes. As the differences between haplotypes increase, the K-mer types in a single haplotype genome become less representative of the entire genome, resulting in a lower completeness value for the haplotypes. Therefore, the completeness values evaluated by Merqury are not shown in Table 1.
