# Supplementary material for: Phased high-quality genome of the gymnosperm Himalayan Yew assists in paclitaxel pathway exploration
Source: Gigascience. 2025 Apr 4;14:giaf026. doi: 10.1093/gigascience/giaf026 (PMC11970372; doi:10.1093/gigascience/giaf026)
Supplement: giaf026_Reviewer_1_Report_Original_Submission — Xuepeng Sun -- 9/4/2024 [file giaf026_reviewer_1_report_original_submission.pdf]

Reviewer Report

Title: Phased High-Quality Genome of the Gymnosperm Himalayan Yew Assists in Paclitaxel Pathway Exploration

Version: Original Submission **Date:** 9/4/2024

Reviewer name: Xuepeng Sun

Reviewer Comments to Author:

Li and colleagues successfully assembled a high-quality diploid genome of Taxus wallichiana and conducted data mining for ODDs, key C4Î²-C20 epoxidases in the paclitaxel synthesis pathway. Overall, this work is solid and engaging, and the data will serve as a valuable resource for future research in the field. However, several concerns about the data are outlined below.
The logic behind haplotype phasing in this study is difficult to follow. The authors used UL reads and hap2 to fill the gaps in hap1, leaving hap1 unresolved, yet they still claim this represents a resolved diploid genome. In my opinion, the most effective approach to achieving a high-quality genome would be to close the gaps in the primary contig assembly, which provides consensus sequences for both haplotypes.
I was surprised to see a large chunk of genome sequences translocated between chromosomes in the two haplotypes (Fig. S2). Given the extremely high percentage of TEs in the genome, the possibility of mis-assembly cannot be ruled out based on the current data. It would be important to verify the coverage at the junction sites using PacBio reads to determine if the read coverage is continuous or interrupted.
Many chromosomes display length variations between haplotypes, so why do the authors believe that Chr12 is the sex chromosome?
The results section includes several methodological details that should be relocated to the methods section or removed if already described.
Line 60-61: This statement is inaccurate, as gap regions are primarily composed of repetitive sequences, which often lack functional genes.
Line 121: It is recommended to specify whether the sequenced tree was male or female.
Line 433: Cell lines?

Methods

Are the methods appropriate to the aims of the study, are they well described, and are necessary controls included? Choose an item.

Conclusions

Are the conclusions adequately supported by the data shown? Choose an item.

Reporting Standards

Does the manuscript adhere to the journal's guidelines on minimum standards of reporting? Choose an item.

Choose an item.

Statistics

Are you able to assess all statistics in the manuscript, including the appropriateness of statistical tests used? Choose an item.

Quality of Written English

Please indicate the quality of language in the manuscript: Choose an item.

Declaration of Competing Interests

Please complete a declaration of competing interests, considering the following questions:

- Have you in the past five years received reimbursements, fees, funding, or salary from an organisation that may in any way gain or lose financially from the publication of this manuscript, either now or in the future?
- Do you hold any stocks or shares in an organisation that may in any way gain or lose financially from the publication of this manuscript, either now or in the future?
- Do you hold or are you currently applying for any patents relating to the content of the manuscript?
- Have you received reimbursements, fees, funding, or salary from an organization that holds or has applied for patents relating to the content of the manuscript?
- Do you have any other financial competing interests?
- Do you have any non-financial competing interests in relation to this paper?

If you can answer no to all of the above, write 'I declare that I have no competing interests' below. If your reply is yes to any, please give details below.

I declare that I have no competing interests

I agree to the open peer review policy of the journal. I understand that my name will be included on my report to the authors and, if the manuscript is accepted for publication, my named report including any attachments I upload will be posted on the website along with the authors' responses. I agree for my report to be made available under an Open Access Creative Commons CC-BY license (http://creativecommons.org/licenses/by/4.0/). I understand that any comments which I do not wish to be included in my named report can be included as confidential comments to the editors, which will not be published.

Choose an item.

To further support our reviewers, we have joined with Publons, where you can gain additional credit to further highlight your hard work (see: https://publons.com/journal/530/gigascience). On publication of this paper, your review will be automatically added to Publons, you can then choose whether or not to claim your Publons credit. I understand this statement.

Yes Choose an item.
