# Supplementary material for: Phased high-quality genome of the gymnosperm Himalayan Yew assists in paclitaxel pathway exploration
Source: Gigascience. 2025 Apr 4;14:giaf026. doi: 10.1093/gigascience/giaf026 (PMC11970372; doi:10.1093/gigascience/giaf026)
Supplement: giaf026_Reviewer_2_Report_Original_Submission — Aleksey Zimin -- 9/11/2024 [file giaf026_reviewer_2_report_original_submission.pdf]

Reviewer Report

Title: Phased High-Quality Genome of the Gymnosperm Himalayan Yew Assists in Paclitaxel Pathway Exploration

Version: Original Submission **Date:** 9/11/2024

Reviewer name: Aleksey Zimin

Reviewer Comments to Author:

The manuscript describes a novel assembly of Himalayan Yew, which is an important source of a widely used cancer drug Paclitaxel. The study is important to further our understanding of how the tree produces the chemical with the eventual aim of synthesizing the drug.
The authors claim that their assembly is the highest quality gymnosperm assembly to date. While the assembly is indeed very contiguous, I have major concerns as to the quality of the assembly and annotation, especially the quality of resolution of haplotypes, and the quality of the annotation.
Major issues:
1. Assembly quality: The authors used hifiasm assembler to perform the initial assembly of the genome without integrating the available Hi-C data. This is not the correct use of the software to perform haplotype phased assembly. Phasing post-assembly with 3D-DNA pipeline is not the correct way to go, because assembled contigs may already have phasing errors. Also filling gaps in one haplotype with sequence from another haplotype defeats the purpose of phased assembly and introduces phasing errors. The correct way to assemble the data is to use -h1 and -h2 options to supply the HiC data concurrently with the PacBio HiFi data for proper haplotype resolution within hifiasm. This results in correct haplotype resolution and prevents phasing errors. The phased assemblies can be then scaffolded with 3D-DNA or similar pipeline separately. THis makes me doubt the conclusions on large scale structiural variation between the haplotypes -- the varioation could simply be due to phasing errors.
2. Annotation quality: Annotation is performed by an ad hoc approach, combining BRAKER2 annotations with Genewise predictions and Stringtie transcript assemblies with CDSs predicted by TransDecoder. The results were combined with EvidenceModeler. The first problem with this approach is that TransDecoder performs poorly on identifying correct ORFs in assembled transcripts without additional extrinsic evidence, such as protein homology. The second problem is that by combining various software packages, the authors put together a custom annotation pipeline. No evidence is provided as to the performance of this custom pipeline. BRAKER3 software has been available for over a year now, and it performs better than BRAKER2.
3. BUSCO analysis using Gymnosperm database is circular. The single-copy orthologs used in the database have been derived from mainly ab initio gene predictions on previously assembled gymnosperm genomes. These genomes have generally been assembled from short-read data and thus these annotations contain many errors. I suggest using more general BUSCO database of orthologs, such as the plantae database.
In conclusion, the claim that this is the highest quality assembly of a gymnosperm genome is not

substantiated by the methods used in the paper. The annotation is performed in an ad hoc way, and I doubt that its results are reliable. Thus my recommendation is to reject the paper.

Methods

Are the methods appropriate to the aims of the study, are they well described, and are necessary controls included? Choose an item.

Conclusions

Are the conclusions adequately supported by the data shown? Choose an item.

Reporting Standards

Does the manuscript adhere to the journal's guidelines on minimum standards of reporting? Choose an item.

Choose an item.

Statistics

Are you able to assess all statistics in the manuscript, including the appropriateness of statistical tests used? Choose an item.

Quality of Written English

Please indicate the quality of language in the manuscript: Choose an item.

Declaration of Competing Interests

Please complete a declaration of competing interests, considering the following questions:

- Have you in the past five years received reimbursements, fees, funding, or salary from an organisation that may in any way gain or lose financially from the publication of this manuscript, either now or in the future?
- Do you hold any stocks or shares in an organisation that may in any way gain or lose financially from the publication of this manuscript, either now or in the future?
- Do you hold or are you currently applying for any patents relating to the content of the manuscript?
- Have you received reimbursements, fees, funding, or salary from an organization that holds or has applied for patents relating to the content of the manuscript?
- Do you have any other financial competing interests?
- Do you have any non-financial competing interests in relation to this paper?

If you can answer no to all of the above, write 'I declare that I have no competing interests' below. If your reply is yes to any, please give details below.

I declare that I have no competing interests

I agree to the open peer review policy of the journal. I understand that my name will be included on my report to the authors and, if the manuscript is accepted for publication, my named report including any attachments I upload will be posted on the website along with the authors' responses. I agree for my report to be made available under an Open Access Creative Commons CC-BY license (http://creativecommons.org/licenses/by/4.0/). I understand that any comments which I do not wish to be included in my named report can be included as confidential comments to the editors, which will not be published.

Choose an item.

To further support our reviewers, we have joined with Publons, where you can gain additional credit to further highlight your hard work (see: https://publons.com/journal/530/gigascience). On publication of this paper, your review will be automatically added to Publons, you can then choose whether or not to claim your Publons credit. I understand this statement.

Yes Choose an item.
